# Efficacy of Cancer Immunotherapy: An Umbrella Review of Meta-Analyses of Randomized Controlled Trials

**DOI:** 10.3390/cancers11111801

**Published:** 2019-11-15

**Authors:** Jong Yeob Kim, Keum Hwa Lee, Michael Eisenhut, Hans J. van der Vliet, Andreas Kronbichler, Gwang Hun Jeong, Jae Il Shin, Gabriele Gamerith

**Affiliations:** 1 Yonsei University College of Medicine, Seoul 03722, Korea; crossing96@yonsei.ac.kr; 2 Department of Pediatrics, Yonsei University College of Medicine, Yonsei-ro 50, Seodaemun-gu, C.P.O. Box 8044, Seoul 03722, Korea; AZSAGM@yuhs.ac; 3 Children’s & Adolescent Services, Luton & Dunstable University Hospital NHS Foundation Trust, Lewsey Road, Luton LU4 ODZ, UK; michael_eisenhut@yahoo.com; 4 Department of Medical Oncology, VU University Medical Center, Cancer Center Amsterdam, 1081 HV Amsterdam, The Netherlands; JJ.vanderVliet@vumc.nl; 5 Department of Internal Medicine IV (Nephrology and Hypertension), Medical University Innsbruck, Anichstraße 35, 6020 Innsbruck, Austria; andreas.kronbichler@i-med.ac.at; 6 College of Medicine, Gyeongsang National University, Jinju 52727, Korea; 7 Department of Internal Medicine V (Hematology & Oncology), Medical University Innsbruck, Anichstraße 35, 6020 Innsbruck, Austria; gabriele.gamerith@i-med.ac.at

**Keywords:** immunotherapy, cancer, umbrella review, meta-analysis

## Abstract

We conducted a systematic review for evidence of the clinical efficacy of cancer immunotherapies. We searched PubMed from inception to 14 February 2018 for meta-analyses of randomized controlled trials (RCTs) of cancer immunotherapies. Re-analyses were performed to estimate the summary effect size under random-effects, the 95% confidence interval (CI), heterogeneity, and the 95% prediction interval, and we determined the strength of the evidence. We examined publication bias and excess significance bias. 63 articles corresponding to 247 meta-analyses were eligible. Nine meta-analyses were classified to have convincing evidence, and 75 were classified as suggestive evidence. The clinical benefit of immunotherapy was supported by convincing evidence in the following settings: anti-PD-1/PD-L1 monoclonal antibody (mAb) therapy for treating advanced melanoma and non-small cell lung cancer (NSCLC), the combination of rituximab and chemotherapy for treating chronic lymphocytic leukemia and B-cell non-Hodgkin’s lymphoma, adoptive cell immunotherapy for NSCLC, and the combination of interferon α and chemotherapy for metastatic melanoma. A further meta-analysis of 16 RCTs showed that anti-PD-1/PD-L1 mAb therapy had a benefit in patients with solid tumors (overall survival; hazard ratio = 0.73, 95% CI: 0.68–0.79; *p* < 0.001), supported by convincing evidence. In the future, rigorous approaches are needed when interpreting meta-analyses to gain better insight into the true efficacy of cancer immunotherapy.

## 1. Introduction

Cancer immunotherapy was first introduced in the late 19th century. Dr. William Coley, after observing cases in which a patient’s sarcoma regressed after being infected with *Streptococcus pyogenes* [1], administered a mixture of inactivated *S. pyogenes* and *Serratia marcescens* known as “Coley’s toxins” to patients with various carcinomas, which resulted in activation of the immune system against the tumor cells [2]. For the last three decades, progress in our understanding of the immune system, cancer etiology, oncogenes, tumor microenvironment, and advances in molecular biology has opened up a new paradigm of cancer immunotherapy and targeted treatments [2,3].

The principal goal of cancer immunotherapy is to enhance the patient’s existing immune response to launch a sustained attack on cancer cells [2,3]. Anti-CD20 monoclonal antibodies (mAbs) bind to CD20 expressed by neoplastic B cells which results in their lysis [3]. Programmed cell death protein 1 (PD-1)/Programmed cell death protein ligand 1 (PD-L1) mAbs abrogate the inhibitory interaction between PD-L1 expressed by tumor cells and immunosuppressive cells in the tumor microenvironment and PD-1 expressed by effector T-cells, thereby enhancing the effector T-cell’s antineoplastic activity [4]. Adoptive cell transfer immunotherapy involves (1) collection of tumor-infiltrating lymphocytes or circulating lymphocytes, (2) their culture/selection/modification/expansion ex vivo, (3) and their (re-)administration to patients [3]. For example, cytokine-induced killer cells (CIK) are mononuclear cells incubated with various cytokines [5], and are sometimes co-cultured with dendritic cells (DCs) to enhance cytocidal effects [6]. Cytokines such as interferon alpha (IFN-α) and interleukin 2 (IL-2) are administered to cancer patients as an immunomodulatory agent to promote various anticancer activities. Tumor antigen vaccines such as DC-based vaccine Sipuleucel-T is applied to promote tumor-specific immune responses [6,7].

Numerous efforts have been made to assess the clinical efficacy of a diverse range of cancer immunotherapies. However, to the best of our knowledge, there has been no effort to summarize and examine the statistical validity of these immunotherapeutic approaches in terms of their potential limitations such as the presence of various biases. For this reason, we performed an umbrella review of all available meta-analyses of randomized controlled trials (RCTs) reporting on the efficacy of cancer immunotherapy to provide an insight into which cancer immunotherapy is truly an effective therapeutic approach.

## 2. Results

A total of 424 articles from a pre-defined PubMed search were screened (Figure S1), and 63 articles were considered eligible, corresponding to 222 original meta-analyses, which were conducted only on RCTs. The eligible meta-analyses also reported 25 original meta-analyses containing non-RCT(s), and re-analysis on only RCTs were reported as the primary outcome. Re-analyses of 247 original meta-analyses corresponding to 1,306 individual study outcomes (excluding non-RCTs) and 324,856 patient data were reported as the primary outcome. It is worth noting that, because there were often several meta-analyses studying a similar topic performed independently by different groups, there were some overlapping RCTs among the meta-analyses of a similar topic. We did not track and count all the RCTs excluding the overlapping ones included in every meta-analysis (with the exception of PD-1/PD-L1 inhibitor OS, PD-1/PD-L1 inhibitor PFS, and DC/CIK OS, as described below), as this was beyond the scope of our umbrella review. Rather, we focused on performing the re-analysis of eligible meta-analyses to verify the statistical validity of each meta-analysis.

The types of immunotherapy were classifiable into five main categories: (1) a mAb group, which included anti-PD-1/PD-L1 mAb and rituximab (15 articles studying the treatment), (2) an adoptive cell immunotherapy group, which included DC/CIK and CIK (15 articles), (3) an immunomodulatory cytokine group, which included IFN-α and IL-2 (25 articles), (4) a vaccine group, which included the DC vaccine Sipuleucel-T (9 articles), and (5) another immunotherapy group in which the types of immunotherapy were inconsistent or not specified in the original study (7 articles). The median number of studies per meta-analysis was 4 (interquartile range (IQR) 3–7), and the median number of total participants per meta-analysis was 885 (IQR 410–1,542). Under the random-effects model, 71 (29%) of the meta-analyses were statistically significant with a *p* < 0.001, while 75 (30%) were statistically significant, with 0.001 < *p* < 0.05. Only 9 (4%) outcomes were determined to have convincing evidence of cancer immunotherapy (6 in the mAb group, 1 in the adoptive cell immunotherapy group, and 2 in the cytokine group) as an effective therapeutic agent compared to the control intervention. Seventy-five (30%) outcomes showed suggestive evidence, 58 (23%) showed weak evidence in favor of immunotherapy, 4 (2%) showed weak evidence in favor of control therapy, and 101 (41%) were not statistically significant. More detailed descriptive statistics according to immunotherapy groups is shown in Table S1 of the Supplementary Materials. Major results of the re-analysis are presented in Table 1, Table 2 and Table 3, and statistically significant results among them are shown in Figures S2–S4 of the Supplementary Materials. All results of the re-analysis are presented in Tables S2–S7 of the Supplementary Materials.

### 2.1. Monoclonal Antibodies

Articles of meta-analyses of anti-PD-1/PD-L1 mAb therapy have been published since 2016, reaching six until 2017 (Table 1 and Table S2, Figure S2). Anti-PD-1/PD-L1 mAbs showed a benefit over chemotherapy in improving progression-free survival (PFS) and the overall response rate (ORR) of advanced melanoma patients with a relatively large effect size, supported by convincing evidence. The anti-PD-l mAb nivolumab improved PFS of melanoma patients compared to ipilimumab. Anti-PD-1/PD-L1 mAbs also showed benefit over chemotherapy in overall survival (OS) and PFS of patients with non-small cell lung cancer (NSCLC), supported by convincing evidence and suggestive evidence, respectively. Wang et al. incorporated data from 10 available trials studying anti-PD-1/PD-L1 mAb therapy in patients with melanoma, NSCLC, and renal cell cancer (RCC) [8]. The study showed that anti-PD-1/PD-L1 mAb therapy was effective at improving PFS, OS, ORR, and stable disease rate in patients with solid cancers with a relatively large study effect, although high in-between heterogeneity existed.

Concerning the efficacy of anti-PD-1/PD-L1 mAb therapy, no study sufficiently contained all available RCTs of interest in one meta-analysis, so we performed new meta-analyses with all eligible RCTs that we found (Figures S5 and S6), including RCTs from the most recent meta-analysis available [9]. Our meta-analysis of 16 RCTs (8,263 patients) of anti-PD-1/PD-L1 mAbs (9 studies with nivolumab, 5 with pembrolizumab, and 2 with atezolizumab) regarding the OS of patients with gastric or gastro-esophageal junction cancer (2 studies), head-and-neck squamous cell carcinoma (1 study), melanoma (4 studies), NSCLC (7 studies), RCC (1 study), and urothelial cancer (1 study) demonstrated a beneficial effect of anti-PD-1/PD-L1 mAbs over conventional therapies (hazard ratio (HR) = 0.73, 95% confidence interval (CI): 0.68 to 0.79; *p* < 0.001), supported by convincing evidence, despite moderate heterogeneity (I^2^ = 39%). Meta-analysis of 18 trials (9,748 patients) regarding an effect on PFS of anti-PD-1/PD-L1 mAbs (10 trials with nivolumab, 6 with pembrolizumab, and 2 with atezolizumab) in patients with gastric or gastro-esophageal junction cancer (2 studies), head-and-neck squamous cell carcinoma (1 study), melanoma (6 studies), NSCLC (7 studies), RCC (1 study), and urothelial cancer (1 study) showed improved PFS (HR = 0.73, 95% CI 0.65 to 0.84; *p* < 0.001). However, unlike the analysis of OS, the meta-analysis of PFS showed high heterogeneity (I^2^ = 87%) and 95% prediction interval (PI), which included the null. Reasons for the high heterogeneity could have been due to the difference in cancer type and the difference in medication. Differences in patient characteristics such as sex or PD-L1 expression level may have also accounted for the high in-between study heterogeneity [9,10].

Nine studies corresponding to 28 meta-analyses assessed the efficacy of anti-CD20 mAb therapy, all of which were performed with rituximab (Table 1 and Table S3, Figure S2). The combination of rituximab and chemotherapy showed a higher response rate and a better PFS compared to chemotherapy alone in the management of chronic lymphocytic leukemia, supported by suggestive evidence, and an improved complete remission rate, supported by convincing evidence. Three studies assessed the induction therapy of non-Hodgkin’s lymphoma. Gao et al. is an updated study of Schulz et al. and contains all its component studies, while Schulz et al. performed subgroup analyses of trials according to lymphoma types [11,12]. Re-analysis of the study of Gao et al. showed that the combination of rituximab and chemotherapy was more effective than chemotherapy alone when considering the improvement in OS and disease control rate (DCR) of B-cell non-Hodgkin’s lymphoma, supported by convincing evidence. In 2011, Hou et al. analyzed trials held in China that did not overlap with the studies analyzed by Gao et al. [13]. Re-analysis of Hou et al. demonstrated the benefit of the combination of rituximab and chemotherapy compared to chemotherapy alone on improving the complete response rate of patients with B-cell non-Hodgkin’s lymphoma. Rituximab maintenance therapy showed an improvement in event-free survival and PFS in patients with diffuse large B-cell lymphoma and showed an improvement in OS of patients with follicular lymphoma.

### 2.2. Adoptive Cell Immunotherapy

The efficacy of adjuvant DC/CIK in treating patients with hepatocellular carcinoma (HCC) after transcatheter arterial chemoembolization (TACE) treatment was shown by the improvement in DCR and 1-year OS (Table 2 and Table S4, Figure S3). In patients with NSCLC, the addition of DC/CIK to standard therapy improved PFS and DCR. Additionally, we performed a meta-analysis of all available RCTs studying the effect of DC/CIK on OS in patients with NSCLC (risk ratio (RR) = 0.82, 95% CI 0.75 to 0.89; *p* < 0.001), which was classified as suggestive evidence because the total number of participants did not exceed 1,000 subjects (Figure S7). The meta-analysis on trials of DC/CIK for various solid cancers was classified as suggestive evidence for the improvement of OS and the objective response rate but was based on a relatively small number of participants (<500) and lacked individual trials addressing each type of cancer. Meta-analyses dealing with the efficacy of adjuvant CIK were performed on trials of HCC patients and showed benefit on OS and recurrence-free survival. In all meta-analyses of DC/CIK or CIK, 95% PI included the null, and the number of total participants was less than 1000.

In seven of 18 articles dealing with adoptive cell immunotherapy, meta-analyses were conducted regardless of the type, such as DC/CIK, CIK, and tumor-infiltrating lymphocytes (Figure S3). Adjuvant adoptive cell immunotherapy showed benefit on the 3-year recurrence rate and 3-year mortality rate of HCC. The benefit of adoptive cell immunotherapy compared to control therapy on 2-year OS of patients with NSCLC was supported by convincing evidence. The addition of postoperative adoptive cell immunotherapy to conventional therapy for treating patients with resected NSCLC resulted in an improvement of OS. adoptive cell immunotherapy treatment for RCC showed a benefit on 1-year OS, supported by suggestive evidence, but this was based on a relatively small number of participants (<500).

### 2.3. Immunomodulatory Cytokines

The clinical efficacy of immunomodulatory cytokines, mostly IFN- α and IL-2, was studied for various cancers types (Table 3 and Table S5, Figure S4). The addition of IFN-α to 5-fluorouracil therapy for colorectal cancer did not show a clinical benefit. IFN-α maintenance therapy showed a clinical benefit on PFS in patients with follicular lymphoma and on the OS of patients with NSCLC. Adjuvant therapy with IFN-α resulted in an improvement in survival after surgical resection or TACE treatment of HCC and showed a decrease in tumor recurrence rate.

Meta-analyses of adjuvant IFN-α therapy for patients with melanoma included a large number of participants (>5000). Adjuvant treatment with IFN-α therapy resulted in an improvement in OS, supported by suggestive evidence, as well as event-free survival, supported by weak evidence because there was no statistically significant component study. In comparison to controls, the addition of adjuvant IFN-α decreased the recurrence rate of melanoma after surgical resection. A benefit of IFN-α on inoperable metastatic RCC as assessed by the mortality and response rate was supported by suggestive evidence. However, it is worth mentioning that targeted therapy (temsirolimus or sunitinib) showed a benefit over IFN-α monotherapy in patients with RCC.

With respect to regimens used for the treatment of advanced melanoma, meta-analyses were performed comparing a combination therapy of cytokines and chemotherapy (“biochemotherapy”) with chemotherapy alone. IFN-α plus chemotherapy showed a benefit over chemotherapy alone on the ORR and complete response rate in patients with metastatic melanoma, supported by convincing evidence. A comparison between IFN-α with dacarbazine and dacarbazine alone also showed an improvement in the ORR and the complete response rate. The addition of IL-2 and IFN-α to standard chemotherapy showed no beneficial effect on the survival rate, though it did improve the response rate in patients with metastatic melanoma (Figure S4).

Adding IL-2 to chemotherapy for the treatment of metastatic colorectal cancer improved the objective response rate. IL-2 monotherapy did not show clinically meaningful benefit for patients with acute myeloid leukemia. IL-2 based regimens resulted in a reduction in 1-year mortality in patients with metastatic RCC. IFN-γ maintenance therapy did not result in a survival benefit in patients with small-cell lung cancer.

### 2.4. Cancer Vaccine

The benefit of vaccine therapy in patients with cancers is best supported by suggestive evidence (Table 3 and Table S6, Figure S4). The meta-analysis of trials of Sipuleucel-T showed improved survival in patients with castration-resistant prostate cancer. DC vaccine therapy showed efficacy in patients with glioma. Other meta-analyses of the efficacy of cancer vaccines were conducted regardless of the specific type of cancer vaccines that were used. Meta-analyses showed that cancer vaccines had a benefit on OS and PFS of patients with NSCLC, compared to a placebo or chemotherapy. No evidence indicated the benefit of a cancer vaccination on RCC outcome.

## 3. Discussion

We systematically analyzed all eligible meta-analyses studying the efficacy of cancer immunotherapy. We assessed 247 meta-analyses by performing re-analysis, including tests for heterogeneity and various biases, and by classifying each re-analysis according to its level of evidence determined by applying criteria to the results of re-analysis. Although more than half of the meta-analyses (142) were statistically significant (*p* < 0.05) in favor of immunotherapy, only 84 were classified as suggestive or convincing evidence due to high heterogeneity (28), not containing any statistically significant studies despite the absence of biases and low heterogeneity (9), implication of publication bias (26), 95% PI including the null (81), or Egger *p*-value and 95% PI being unavailable because analysis was performed on only two component studies (18). Evidence for excess significance (ES) was found in only one study. Convincing evidence was found in the following nine comparisons: anti-PD-1/PD-L1 mAb therapy versus chemotherapy on PFS and ORR in patients with advanced melanoma, anti-PD-1/PD-L1 mAb therapy versus chemotherapy on the OS of patients with NSCLC, rituximab plus chemotherapy versus chemotherapy on the complete remission rate in patients with chronic lymphocytic leukemia, rituximab plus chemotherapy versus chemotherapy on the OS and DCR of patients with B-cell non-Hodgkin’s lymphoma, adoptive cell immunotherapy versus conventional therapy on 2-year OS of patients with NSCLC, and IFN-α plus chemotherapy versus chemotherapy on ORR and complete response rate of patients with metastatic melanoma.

For 25 meta-analyses including non-RCTs in their component study, two types of re-analyses were performed: (1) with RCTs only and (2) with RCTs and non-RCTs altogether. We reported the former as the main outcome to minimize the selection bias and effect of possible confounding factors within trials [14]. While 15 out of 25 re-analyses including only RCTs showed a level of evidence consistent with their RCT plus non-RCT counterpart, the other 10 showed a change in their level of evidence (eight being downgraded, two being upgraded). Details are provided in Table S8.

In our study, 195 (79%) meta-analyses showed low or moderate heterogeneity (I^2^ < 50%) between individual studies, and 52 (21%) meta-analyses showed large heterogeneity (I^2^ > 50%). Twenty-six (11%) meta-analyses showed discrepant results between the fixed and random model, 15 of which had large heterogeneity between trials. Of 52 meta-analyses with large heterogeneity, 22 were performed with a fixed model in their original paper. Using a fixed-effects model in high heterogeneity without fair justification may produce overly precise summary effects whose CI is too narrow, and may give the wrong impression that common treatment effects exist when there may be a real difference in the treatment effect between trials [15]. The random-effects model is an appropriate choice for high between-study heterogeneity since it can account for a genuine difference in treatment effects such as those caused by differences in study population, differences in the intervention such as different doses, or differences in follow-up duration.

While the summary effects of random-effects meta-analysis represent the average effect of included studies, 95% PI estimates the treatment effect of individual studies in future settings [15]. A 95% PI encompasses the full distribution of effects in a random-effects meta-analysis [16] and further accounts for between-study heterogeneity in evaluating the uncertainty of expected effects in the future [17], covering a wider range than CI in the case of high in-between study heterogeneity. 95% PI including the null value suggests that there may be settings where the intervention effect is null or even in the opposite direction, and requires further study for the identification of the causes of heterogeneity. 95% PI excluding the null suggests that the treatment effect is beneficial in at least 95% of the future studies, and gives the conclusion that the results of treatment effects are fairly consistent, even when significant between-study heterogeneity is present [15]. It should be noted that out of our 146 re-analyses showing statistical significance, 95% PI excluded the null in only 43 (29%) of the cases. PI is assessed as potentially the most relevant and complete statistical inference to be drawn from random-effects meta-analyses [18] but was not represented in any of the original meta-analyses included in this review. Future studies need to represent the PI in their meta-analyses of cancer immunotherapy for a better prediction of the true treatment effect [19].

Egger’s test of funnel plot assumes that the accuracy in estimating the true treatment effect increases as the sample size of the component studies increases, and assesses a specific type of heterogeneity compared to the overall test of heterogeneity [20]. The presence of publication bias may also indicate the presence of reporting bias, selective outcome reporting, and selective analysis reporting. However, other factors such as genuine heterogeneity or chance may also contribute to publication bias. A cautious approach is needed when examining publication bias, especially when between-study heterogeneity is large [21]. Among the original meta-analyses examined in our study, the Egger *p*-value was represented in only 18 (7%) of the meta-analyses. In many cases, publication bias was measured by mere visual examination of the funnel plot or the Egger *p*-value was calculated on only some of the outcomes in a study. A thorough examination of Egger *p*-values is needed when one assesses publication bias [20].

Precautions should be taken in interpreting the results of our study. First, the type of metrics used in the analysis should be appreciated. In our study, the odds ratio (OR) was applied in 53 (22%) of the meta-analyses and RR or HR was applied in the residual 194 (78%). Mistaking OR as RR or HR may result in unwanted exaggeration of the effect size [22]. Second, one should note that the level of evidence and effect size represents the efficacy of the intervention relative to the control arm regimen rather than absolute efficacy of the intervention. For example, while one re-analysis showed that IFN-α therapy was beneficial on the OS of RCC patients compared to conventional therapy, supported by suggestive evidence, another re-analysis showed targeted therapy was more beneficial on the OS of RCC patients compared to IFN-α therapy, supported by weak evidence (Table 2). When choosing an optimal therapeutic intervention, all possible treatments should be taken into consideration.

In this review, we have attempted to assess the true efficacy of several cancer immunotherapeutic approaches, but the study is not without limitations. First, we focused on meta-analyses on the subject, and therefore individual trials not included in meta-analyses would have been overlooked in our study. Second, we did not include meta-analyses if they did not provide sufficient individual study data necessary for re-analysis. Third, although we prioritized RCTs over other studies as the subject of re-analysis, we did not apply criteria for assessing qualities of individual component RCTs. Fourth, some statistical limitations exist. ES and 95% PI could not be obtained in the meta-analyses of two studies, so these meta-analyses were at best classified as weak evidence. There are reports that the power of Egger’s test for detecting publication bias is too low when there are fewer than ten studies [21], and the test for ES also typically has low power when there are few studies with a significant effect [23]. Finally, we did not assess the possible differences in the immunotherapy effects resulting from different biomarker expression levels. For example, higher PD-L1 expression on the tumor or immunosuppressive myeloid cells in the tumor microenvironment may correlate with a higher response rate to anti-PD-1/PD-L1 mAb therapy [10]. In one meta-analysis of anti-PD-1/PD-L1 mAb, one component trial only accepted patients with tumors having at least 1% of tumor cells positive for PD-L1, while the other trials accepted patients regardless of PD-L1 expression [24]. Such circumstances may contribute to significant between-study heterogeneity, which leads to difficulties in interpreting the potential presence of biases.

## 4. Materials and Methods 

This systematic review was performed according to a pre-specified protocol registered at the International Prospective Register of Systematic Reviews [25] (registration ID: CRD42018096274). The reporting was done according to the Preferred Reporting Items for Systematic Reviews and Meta-Analyses (PRISMA) guidelines (Table S9) [26].

We identified meta-analyses studying the efficacy of cancer immunotherapy and performed re-analyses including statistical tests for various biases to evaluate its power of evidence. We also performed additional meta-analyses of RCTs in cases where no single meta-analysis effectively covered all trials on a subject, by combining component trials from meta-analyses of interest while excluding overlapping trials. We set criteria based on our statistical tests and determined how reliable meta-analyses could be trusted as evidence for assessing the true clinical effect of the treatment.

### 4.1. Search Strategy and Selection Criteria

Two investigators (JI Shin and JY Kim) independently searched PubMed from inception to 14 February 2018 for any meta-analyses of RCTs that reported efficacy endpoints of cancer immunotherapy. The search was limited to articles written in English. We used the following search strategy: (randomized OR randomized controlled trial) AND (meta OR meta-analysis) AND cancer AND (immunotherapy OR programmed cell death protein OR PD-1 OR PD-L1 OR CD20 OR rituximab OR cytokine-induced killer cell OR CIK OR dendritic cells co-cultured with cytokine-induced killer cells OR DC/CIK OR TIL OR Interferon-alpha OR IFN-α OR interleukin-2 OR IL-2 OR cancer vaccine OR dendritic cell). We then selected eligible articles by first examining the title, then the abstract, and then the full text. Any discrepancies between the two investigators (JI Shin and JY Kim) were resolved by face-to-face discussion. A search of the EMBASE database did not result in additional included meta-analyses in this analysis, as all additional identified articles did not meet the inclusion criteria. They were either conference publications or did not provide enough data for re-analyses.

Articles were considered eligible if they included meta-analyses conducted with systematic methods. Meta-analyses conducted on RCTs were deemed eligible. Meta-analyses of observational studies and retrospective studies were not included. Only meta-analyses containing endpoints regarding the efficacy of cancer immunotherapy treatment, such as OS, PFS, ORR, and DCR were included. Outcomes of individual trials had to be reported in metrics such as HR, RR, or OR. Articles of meta-analyses on trials of various cancer immunotherapies were included regardless of the immunotherapy setting, such as induction therapy, maintenance/salvage therapy, and pre/postsurgical adjuvant therapy. Articles where immunotherapy trials were included in a subgroup of meta-analyses were also included. When all component studies of a meta-analysis were included in another meta-analysis, the smaller meta-analysis was excluded when the results of both were comparable.

From eligible articles, we extracted the title, first author, and year of publication. For each meta-analysis, we extracted the following: intervention therapy, control therapy, cancer of interest, number of patients, number of studies, outcome of interest, type of outcome metrics, analysis model, effect size and 95% CI of each RCT, summary effect size, *p*-value, I^2^ for evaluation of heterogeneity, and Egger *p*-value. 

### 4.2. Data Analysis

From each article, we calculated the summary effect size and its 95% CI and *p*-Value with both fixed and random-effects models. The effect size under a random-effects model was assessed for determining the level of evidence. Results were obtained with identical type of metrics as in the original analyses, and were calculated from the raw data of individual component trials. All reported *p*-Values were two-sided. Analyses used Comprehensive Meta-Analysis v3.3.070 and Microsoft Excel v16.0.

We performed a Q-test for the evaluation of heterogeneity and calculated the I^2^ metrics [27]. I^2^ ranged between 0% and 100% and described the percentage of variability in a study estimate that was due to between-study heterogeneity. An I^2^ value below 50% represented low or moderate heterogeneity while a value above 50% represented large heterogeneity [28]. The 95% PI, which estimates the range where a true effect of the intervention is to be expected for 95% of similar studies in the future [18], was also estimated for further exploration. PIs including the null value suggested that the measured effect might not be reproducible in future trials. Conversely, a PI of 95% excluding the null suggested that the effect would persist in at least 95% of the future studies, and therefore we concluded that the effect of the intervention was consistent. 

We also assessed whether the summary outcome of a random-effects meta-analysis and outcome of its largest component study showed concordance in terms of statistical significance (*p* < 0.05). We also assessed the presence of publication bias, measured by a regression asymmetry test proposed by Egger et al. [20]. Publication bias was claimed at Egger *p*-Value < 0.10.

We performed a test for ES to evaluate whether the number of studies reporting nominally significant results (*p* < 0.05) was greater compared to the expected number of studies reporting statistically significant results [29]. The expected number of statistically significant studies was calculated as the sum of the power of individual component studies [23]. In two cases where the power of all individual studies was not available (Vidal et al. 2017, Rituximab maintenance versus observation or treatment only at relapse, follicular lymphoma; and Mocellin et al. 2013, adjuvant IFN-α versus conventional therapy, metastatic melanoma; see Table 1 and Table 2), we assumed that the power of each study could be replaced by the power of the study with the largest number of participants [30]. The power of individual studies was estimated in terms of non-central t distribution, with the aid of G*Power ver. 3.0.10 [31]. Statistic A was calculated in terms of χ2 distribution, and ES was claimed at *p* < 0.1 with the number of observed statistically significant studies larger than the expected number of significant studies [23]. The presence of ES under low or moderate heterogeneity (I^2^ < 50%) suggested that the meta-analysis was affected by some kind of bias, such as publication bias, selective analysis, or outcome reporting bias [30]. It is worth noting that we performed post-hoc power analysis to assess excess significance bias within the meta-analyses, rather than to distinguish between the true negative effects and false-negative effects in negative associations, which is a controversial statistical method that may provide misleading information.

If an original meta-analysis was based on one or more non-RCT(s), re-analysis was conducted on both conditions: (1) with RCTs only and (2) with all component studies including non-RCT(s), although the former was reported as the main outcome. We also obtained the quality of the individual studies in the eligible meta-analyses using the Cochrane risk of bias tool. Its results are reported in Table S10. 

We applied the following criteria for each meta-analysis to determine the strength of its evidence as a proof of the efficacy of immunotherapy compared to control therapy. The respective algorithm is shown in Figure S8.

**Weak evidence**: statistically significant result (random-effects *p*-Value < 0.05)

**Suggestive evidence**: (1) statistically significant result (random-effects *p*-Value < 0.05), (2) low or moderate heterogeneity (I^2^ < 50), (3) no evidence of publication bias (Egger *p*-Value > 0.1), and (4) no evidence of ES (ES *p*-Value > 0.1)

**Convincing evidence**: (1) highly statistically significant result (random-effects *p*-Value < 0.001), (2) low or moderate heterogeneity (I^2^ < 50), (3) no evidence of publication bias (Egger *p*-Value > 0.1), (4) no evidence of ES (ES *p*-Value > 0.1), (5) 1,000 or more participants, (6) summary outcome shows concordant result with the largest study, and (7) 95% PI excludes the null hypothesis. 

When a meta-analysis had no evidence of publication bias or ES but had high in-between study heterogeneity (I^2^ > 50), we rechecked the results of its component studies to find out whether the high heterogeneity was due to the differences in the direction of effects or due to the differences in the size of the associations. When the number of statistically significant component studies was the same or greater than the number of studies, which were not significant or significant in the opposite direction, the comparison was classified as suggestive evidence, or as convincing evidence if further criteria were also met. When no statistically significant component study was observed in a meta-analysis, the analysis was at best classified as weak evidence. 

## 5. Conclusions

We comprehensively re-analyzed meta-analyses of various cancer immunotherapeutic approaches by assessing the presence of various potential biases affecting the efficacy of immunotherapy compared to control therapy, and determined how reliable the analysis could be trusted by defining the level of evidence for each comparison. Even though more than half of the 247 meta-analyses reporting on the efficacy of immunotherapy showed statistically significant results, only nine provided convincing evidence while 75 highlighted suggestive evidence. One cannot state that treatments supported by a low level of evidence are not effective, but there is still some uncertainty in them that should be resolved. Further studies with accurate assessment of potential biases and appropriate interpretation of heterogeneity are needed to confirm the true effect of cancer immunotherapy.

## Figures and Tables

**Table 1:** Main findings and level of evidence reported in meta-analyses of cancer therapeutic anti-PD-1/PD-L1 and anti-CD20 monoclonal antibodies.

Author, Year	Comparison	Cancer Type	RCT *n*.	Intervention /Control	Outcome	Metrics	R/N/S †	R *p*-Value	R SE (95% CI)	I2(%)	95% Prediction interval	Egger *p*-Value	Excess Significance	Level of Evidence
**Anti-PD-1/PD-L1 mAb**														
Guan et al. 2016	Anti-PD-1/PD-L1 vs CTx	Advanced melanoma	3	843/699	PFS	HR	0/1/3	<0.001	0.50 (0.43–0.59)	18	0.32–0.80	0.24	*p* > 0.1	Convincing
Guan et al. 2016	Anti-PD-1/PD-L1 vs CTx	Advanced melanoma	3	843/699	Overall response	RR	0/0/4	<0.001	3.23 (2.37–4.41)	0	1.64–6.39	0.19	*p* > 0.1	Convincing
Hao et al. 2017	Nivolumab vs ipilimumab	Metastatic advanced melanoma	2	871/593	PFS	HR	0/0/2	<0.001	0.58 (0.48–0.69)	0	-	-	*p* > 0.1	Weak
Hao et al. 2017	Nivolumab + ipilimumab vs ipilimumab	Metastatic advanced melanoma	2	410/362	PFS	HR	0/0/2	<0.001	0.40 (0.31–0.52)	0	-	-	NA	Weak
Hao et al. 2017	Nivolumab or pembrolizumab vs CTx	Metastatic advanced melanoma	3	843/520	PFS	HR	0/1/2	<0.001	0.43 (0.37–0.50)	0	0.16–1.14	0.09	NA	Weak
Wang et al. 2017	Anti-PD-1/PD-L1 vs CT	Melanoma	6	1198/974	Objective response	RR	0/0/6	<0.001	2.89 (2.03–4.11)	70	0.98–8.52	0.51	*p* > 0.1	Suggestive
Yun et al. 2016	Anti-PD-1 or Anti-CTLA-4 vs CTx or VAX	Metastatic, unresectable, cutaneous melanoma	4	1328/923	OS	RR	0/0/4	0.001	0.72 (0.60–0.88)	83	0.30–1.75	0.25	*p* > 0.1	Suggestive
Yun et al. 2016	Anti-PD-1 or Anti-CTLA-4 vs CTx or VAX	Metastatic, unresectable, cutaneous melanoma	6	1961/1235	PFS	RR	0/2/4	<0.001	0.84 (0.77–0.92)	84	0.61–1.16	0.17	*p* > 0.1	Suggestive
Zhuansun et al. 2017	Anti-PD-1/PD-L1 vs CTx	Pretreated advanced NSCLC	4	1261/913	OS	HR	0/1/3	<0.001	0.68 (0.61–0.75)	0	0.53–0.86	0.99	*p* > 0.1	Convincing
Zhuansun et al. 2017	Anti-PD-1/PD-L1 vs CTx	Pretreated advanced NSCLC	4	1261/913	PFS	HR	0/2/2	0.009	0.81 (0.70–0.95)	57	0.45–1.49	0.89	*p* > 0.1	Suggestive
Wang et al. 2017	Anti-PD-1/PD-L1 vs CT	Melanoma or NSCLC or RCC	10	3105/2141	PFS	HR	0/4/6	<0.001	0.65 (0.53–0.79)	81	0.33–1.26	0.23	*p* > 0.1	Suggestive
Wang et al. 2017	Anti-PD-1/PD-L1 vs CT	Melanoma or NSCLC or RCC	9	2035/1812	Objective response	RR	0/0/9	<0.001	2.92 (2.07–4.12)	80	0.93–9.19	0.55	*p* > 0.1	Suggestive
Wang et al. 2017	Anti-PD-1/PD-L1 vs CT	Melanoma or NSCLC or RCC	9	2035/1812	Stable disease rate	RR	0/4/5	<0.001	0.58 (0.45–0.75)	81	0.24–1.36	0.59	*p* > 0.1	Suggestive
Anti-PD-1/PD-L1 solid tumor OS||	Anti-PD-1/PD-L1 vs CT	Gastric or gastro-esophageal junction cancer or head-and-neck squamous cell carcinoma or melanoma or NSCLC or RCC or urothelial cancer	16	4681/3582	OS	HR	0/3/13	<0.001	0.73 (0.68–0.79)	39	0.59–0.92	0.72	*p* > 0.1	Convincing
Anti-PD-1/PD-L1 solid tumor PFS||	Anti-PD-1/PD-L1 vs CT	Gastric or gastro-oesophageal junction cancer or head-and-neck squamous cell carcinoma or melanoma or NSCLC or RCC or urothelial cancer	18	5672/4076	PFS	HR	0/9/10	<0.001	0.73 (0.65–0.84)	87	0.41–1.30	0.25	p > 0.1	Suggestive
**Anti-CD20 mAb**														
Bauer et al. 2012	Rituximab + CTx vs CTx	Chronic lymphocytic leukemia	3	710/711	PFS	HR	0/1/2	<0.001	0.65 (0.52–0.83)	50	0.06–7.00	0.75	*p* > 0.1	Suggestive
Bauer et al. 2012	Rituximab + CTx vs CTx	Chronic lymphocytic leukemia	3	710/711	Overall response	RR	0/1/2	<0.001	1.14 (1.08–1.20)	0	0.81–1.61	0.63	*p* > 0.1	Suggestive
Nunes et al. 2015	Rituximab + CTx vs CTx	Chronic lymphocytic leukemia	4	1231/1202	Complete remission	OR	0/1/3	<0.001	2.59 (2.14–3.14)	0	1.70–3.96	0.13	*p* > 0.1	Convincing
Gao et al. 2010	Rituximab + CTx vs CTx	B-cell non-Hodgkin’s lymphoma	11	2486/2447	OS	RR	0/6/5	<0.001	1.08 (1.05–1.11)	20	1.02–1.14	0.28	*p* > 0.1	Convincing
Gao et al. 2010	Rituximab + CTx vs CTx	B-cell non-Hodgkin’s lymphoma	11	2470/2333	DCR	RR	0/4/7	<0.001	1.36 (1.26–1.46)	51	1.11–1.67	0.21	*p* > 0.1	Convincing
Hou et al. 2011	Rituximab + CTx vs CTx	B-cell non-Hodgkin’s lymphoma	7	178/179	Complete response	OR	0/5/2	<0.001	2.99 (1.90–4.71)	0	1.65–5.43	0.54	*p* > 0.1	Suggestive
Schulz et al. 2007	Rituximab + CTx vs CTx	Indolent or mantle cell lymphoma	7	994/949	OS	HR	0/4/3	0.001	0.70 (0.57–0.87)	19	0.46–1.08	0.82	*p* > 0.1	Suggestive
Schulz et al. 2007	Rituximab + CTx vs CTx	Indolent or mantle cell lymphoma	7	978/935	DCR	HR	0/1/6	<0.001	0.70 (0.59–0.84)	72	0.40–1.24	0.51	*p* > 0.1	Suggestive
Vidal et al. 2017	Rituximab maintenance vs obs or treatment only at relapse	Follicular lymphoma	9	1145/1170	OS	HR	0/8/1	0.007	0.79 (0.66–0.94)	0	0.64–0.97	0.91	*p* > 0.1	Suggestive
Zhou et al. 2017	Rituximab maintenance vs obs	Diffuse large B-Cell lymphoma	3	658/661	PFS	HR	0/1/2	0.017	0.71 (0.54–0.94)	43	0.05–10.68	0.67	*p* > 0.1	Suggestive
Zhou et al. 2017	Rituximab maintenance vs obs	Diffuse large B-Cell lymphoma	4	735/686	Event-free survival	HR	0/3/1	0.004	0.80 (0.69–0.93)	0	0.58–1.11	0.78	*p* > 0.1	Suggestive

Abbreviations: RCT, randomized controlled trial; *n*., number; SE, standard effect; CI, confidence interval; M, model; F, fixed effect; R, random effect; NA, not available; C, concordance with largest study; Y, concordant with largest study; N, not concordant with largest study; OS, overall survival; PFS progression-free survival; RR, risk ratio; HR, hazard ratio; OR, odds ratio; CTx, chemotherapy; CT, conventional therapy; VAX, vaccine; obs; NSCLC, non-small cell lung cancer; RCC, renal cell cancer. † Number of individual studies of effect size with statistically significant direction in favor of control therapy/not statistically significant/statistically significant in favor of immunotherapy. ||Re-analysis was performed on all eligible RCTs we found. References are provided in the Reference Appendix of the Supplementary Materials. All *p*-values are two-sided.

**Table 2:** Main findings and level of evidence reported in meta-analyses of adoptive cell immunotherapy for cancer.

Author, Year	Comparison	Cancer Type	RCT *n*.	Intervention /Control	Outcome	Metrics	R/N/S †	R *p*-Value	R SE (95% CI)	I^2^(%)	95% Prediction Interval	Egger *p*-Value	Excess Significance	Level of Evidence
**Dendritic cells with cytokine-induced killer cells**														
Su et al. 2016	DC/CIK vs obs with backbone TACE	HCC	5	250/245	DCR	OR	0/3/2	0.033	1.84 (1.05–3.24)	18	0.35–9.60	0.55	*p* > 0.1	Suggestive
Su et al. 2016	DC/CIK vs obs with backbone TACE	HCC	3	104/106	1-year OS	OR	0/3/0	0.027	2.00 (1.08–3.70)	0	0.04–107.12	0.12	*p* > 0.1	Weak
Wang et al. 2015	DC/CIK + CT vs CT	NSCLC	5	191/192	PFS	HR	0/2/3	<0.001	0.53 (0.39–0.71)	0	0.32–0.86	0.76	*p* > 0.1	Suggestive
Zheng et al. 2015	DC/CIK + CTx vs CTx	NSCLC	3	141/141	DCR	RR	0/1/2	0.007	1.26 (1.07–1.50)	10	0.36–4.47	0.22	*p* > 0.1	Suggestive
DC/CIK NSCLC OS||	DC/CIK + CT vs CT	NSCLC	10	387/427	Longest OS reported	RR	0/7/3	<0.001	0.82 (0.75–0.89)	0	0.74–0.90	0.19	*p* > 0.1	Suggestive
Lan et al. 2015	DC/CIK + CTx vs CTx	Solid tumor, NSCLC or rectal cancer or colorectal cancer or colon cancer or breast cancer or gastric cancer	5	195/202	3-year OS	OR	0/4/1	0.007	0.37 (0.18–0.77)	35	0.06–2.51	0.28	*p* > 0.1	Suggestive
Lan et al. 2015	DC/CIK + CTx vs CTx	Solid tumor, NSCLC or rectal cancer or colorectal cancer or colon cancer or breast cancer or gastric cancer	6	207/224	Overall response	OR	0/5/1	0.005	0.54 (0.35–0.83)	0	0.30–0.99	0.36	*p* > 0.1	Suggestive
**Cytokine-induced killer cells**														
Li et al. 2016	Adjuvant CIK vs no AT	Treated HCC, Barcelona-clinic liver cancer B or earlier stage	7	460/405	PFS	RR	0/5/2	0.004	0.76 (0.63–0.91)	66	0.45–1.29	0.02	*p* > 0.1	Weak
Li et al. 2016	Adjuvant CIK vs no AT	Treated HCC, Barcelona-clinic liver cancer B or earlier stage	5	380/335	OS	RR	0/3/2	0.021	0.78 (0.64–0.96)	42	0.44–1.39	0.04	*p* > 0.1	Weak
Wang et al. 2016	CIK vs no AT after resection	Resected HCC	5	402/357	3-year OS	RR	0/5/0	0.010	1.15 (1.03–1.28)	0	0.97–1.36	0.91	*p* > 0.1	Weak
Wang et al. 2016	CIK vs no AT after resection	Resected HCC	5	402/357	3-year recurrence-free survival	RR	0/4/1	0.007	1.33 (1.08–1.64)	7	0.90–1.98	0.87	*p* > 0.1	Suggestive
Yu et al. 2017	Adjuvant CIK + CT vs CT	HCC	7	451/422	OS	HR	0/3/4	<0.001	0.64 (0.51–0.82)	50	0.34–1.23	0.25	*p* > 0.1	Suggestive
**Other adoptive cellular immunotherapies**														
Yuan et al. 2017	Postoperative ACI (CIK or LAK + IL-2 or lymphocytes) vs no AT	Pretreated HCC, not advanced	6	407/362	3-year mortality	RR	0/5/1	0.009	0.71 (0.55–0.92)	0	0.49–1.02	0.81	*p* > 0.1	Suggestive
Yuan et al. 2017	Postoperative ACI (CIK or LAK + IL-2 or lymphocytes) vs no AT	Pretreated HCC, not advanced	6	407/362	3-year recurrence rate	RR	0/5/1	0.001	0.81 (0.72–0.92)	0	0.68–0.97	0.30	*p* > 0.1	Suggestive
Zeng et al. 2016	Postoperative ACI (AKT-DC or DC/CIK or LAK + IL-2 or TIL + rIL-2) + CT vs CT	Resected NSCLC	4	234/238	OS	HR	0/3/1	0.013	0.59 (0.39–0.89)	40	0.14–2.56	0.16	*p* > 0.1	Suggestive
Zhao et al. 2017	ACI (LAK + IL-2 or DC/CIK or CIK or AKT or TIL) vs CT	NSCLC, operated or non-operated	11	669/755	2-year OS	RR	0/5/6	<0.001	1.43 (1.30–1.58)	0	1.28–1.61	0.24	*p* > 0.1	Convincing
Zhao et al. 2017	ACI (LAK + IL-2 or DC/CIK or CIK or AKT or TIL) vs CT	NSCLC, operated or non-operated	8	529/613	3-year OS	RR	0/5/3	<0.001	1.45 (1.24–1.69)	0	1.19–1.76	0.31	*p* > 0.1	Suggestive
Zhao et al. 2017	ACI (LAK + IL-2 or DC/CIK or CIK or AKT or TIL) vs CT	NSCLC, operated or non-operated	4	187/229	1-year PFS	RR	0/2/2	0.031	1.46 (1.24–1.72)	0	1.02–2.09	0.16	*p* > 0.1	Suggestive
Tang et al. 2013	ACI (autolymphocyte or LAK or CIK) vs no ACI	Metastatic RCC	4	235/224	1-year OS	RR	0/3/1	<0.001	1.33 (1.15–1.54)	0	0.97–1.83	0.205	*p* > 0.1	Suggestive

Abbreviations: RCT, randomized controlled trial; *n*., number; SE, standard effect; CI, confidence interval; M, model; F, fixed effect; R, random effect; NA, not available; C, concordance with largest study; Y, concordant with largest study; N, not concordant with largest study; OS, overall survival; PFS progression-free survival; RR, risk ratio; HR, hazard ratio; OR, odds ratio; TACE, Transcatheter arterial chemoembolization; CT, conventional therapy; CTx, chemotherapy; AT, adjuvant therapy; ACI, adoptive cell immunotherapy; CIK, cytokine-induced killer cells; LAK, lymphokine-activated killer cells; IL-2, Interleukin-2; DC/CIK, dendritic cells with cytokine-induced killer cells; AKT, activated killer T-cells; TIL, tumor-infiltrating lymphocytes; HCC, hepatocellular carcinoma; NSCLC, non-small cell lung cancer; RCC, renal cell cancer. † Number of individual studies of effect size with statistically significant direction in favor of control therapy/not statistically significant/statistically significant in favor of immunotherapy. ||Re-analysis was performed on RCTs from all eligible meta-analyses we found. References are provided in the Reference Appendix of the Supplementary Materials. All *p*-Values are two-sided.

**Table 3:** Main findings and level of evidence reported in meta-analyses of cancer therapeutic immunomodulatory cytokines and cancer vaccines.

Author, Year	Comparison	Cancer Type	RCT *n*.	Intervention /Control	Outcome	Metrics	R/N/S †	R *p*-Value	R SE (95% CI)	I^2^(%)	95% Prediction Interval	Egger *p*-Value	Excess Significance	Level of Evidence
**Interferon-α**														
Thirion, et al. 2000	IFN-α + 5FU vs leucovorin + 5FU	Colorectal cancer	7	744/744	OS	HR	0/7/0	0.403	1.02 (0.98–1.05)	0	0.96–1.08	0.38	*p* > 0.1	No association
Thirion, et al. 2000	IFN-α + 5FU vs 5FU, with or without leucovorin in both arms	Colorectal cancer	12	879/887	OS	HR	0/12/0	0.203	1.02 (0.99–1.05)	0	0.99–1.06	0.97	*p* > 0.1	No association
Jiang, et al. 2013	Post-surgical adjuvant IFN (mostly IFN-α) vs *p* after surgical resection or TACE	Pretreated HCC, viral hepatitis related	9	498/451	Mortality	OR	0/6/3	<0.001	0.43 (0.32–0.56)	0	0.31–0.59	0.91	*p* > 0.1	Suggestive
Jiang, et al. 2013	Post-surgical adjuvant IFN (mostly IFN-α) vs *p* after surgical resection or TACE	Pretreated HCC, viral hepatitis related	9	499/476	Recurrence rate	OR	0/8/1	0.003	0.66 (0.51–0.87)	0	0.48–0.92	0.08	*p* > 0.1	Weak
Ives, et al. 2007	IFN-α + CTx vs CTx	Metastatic melanoma	11	683/607	Overall response	OR	0/8/3	<0.001	0.58 (0.44–0.77)	0	0.40–0.84	0.49	*p* > 0.1	Convincing
Ives, et al. 2007	IFN-α + CTx vs CTx	Metastatic melanoma	10	662/583	Complete response	OR	0/8/2	<0.001	0.33 (0.19–0.57)	0	0.17–0.64	0.54	*p* > 0.1	Convincing
Ives, et al. 2017	Adjuvant IFN-α vs obs	High-risk, malignant melanoma	18	4520/3179	OS	HR	0/17/1	0.017	0.91 (0.84–0.98)	0	0.83–0.99	0.22	*p* > 0.1	Suggestive
Ives, et al. 2017	Adjuvant IFN-α vs obs	High-risk, malignant melanoma	18	4520/3177	Event-free survival	HR	0/18/0	<0.001	0.86 (0.80–0.92)	0	0.79–0.93	0.23	*p* > 0.1	Weak
Mocellin, et al. 2013	Adjuvant IFN-α vs CT	Metastatic melanoma	15	5412/3771	OS	HR	1/10/4	0.004	0.91 (0.85–0.97)	5	0.82–1.00	0.06	*p* > 0.1	Weak
Mocellin, et al. 2013	Adjuvant IFN-α vs CT	Metastatic melanoma	17	5638/3963	Disease-free survival	HR	0/10/7	<0.001	0.82 (0.77–0.88)	13	0.73–0.93	0.03	*p* > 0.1	Weak
Pirard, et al. 2004	Postsurgical adjuvant IFN-α vs control	Postsurgical Melanoma, stage IV unincluded	10	1483/1508	Recurrence rate	OR	0/7/3	<0.001	0.74 (0.62–0.88)	17	0.53–1.03	0.68	*p* > 0.1	Suggestive
Wheatley, et al. 2003	IFN-α vs obs	Metastatic, high-risk melanoma	14	3144/2037	Recurrence-free survival	HR	0/11/3	<0.001	0.92 (0.88–0.97)	5	0.86–0.99	0.07	NA	Weak
Xin, et al. 2016	IFN-α + dacarbazine vs dacarbazine	Cutaneous malignant melanoma	8	438/357	Overall response	RR	0/6/2	<0.001	1.59 (1.21–2.09)	0	1.13–2.24	0.40	*p* > 0.1	Suggestive
Xin, et al. 2016	IFN-α + dacarbazine vs dacarbazine	Cutaneous malignant melanoma	8	438/357	Complete response	RR	0/6/2	<0.001	3.12 (1.75–5.56)	0	1.46–6.66	0.52	*p* > 0.1	Suggestive
Baldo, et al. 2010	IFN-α maintenance therapy + CT vs CT or obs	Follicular lymphoma	6	686/671	PFS	HR	0/3/3	<0.001	0.65 (0.54–0.79)	31	0.42–1.02	0.14	*p* > 0.1	Suggestive
Rossi, et al. 2010	IFN-α maintenance or consolidation therapy vs *p* or obs	NSCLC	4	253/235	OS	HR	0/3/1	0.016	0.78 (0.64–0.96)	0	0.50–1.22	0.51	*p* > 0.1	Suggestive
Canil, et al. 2010	IFN-α vs CT	Inoperable RCC, metastatic or advanced	6	472/475	Mortality	HR	0/4/2	0.001	0.79 (0.69–0.91)	4	0.64–0.99	0.65	*p* > 0.1	Suggestive
Canil, et al. 2010	IFN-α vs CT	Inoperable RCC, metastatic or advanced	7	493/496	Response	OR	0/3/4	<0.001	6.87 (3.29–14.35)	0	2.61–18.05	0.47	*p* > 0.1	Suggestive
Unverzagt, et al. 2017	IFN-α monotherapy vs standard targeted therapy	Metastatic RCC	2	582/584	OS	HR	1/1/0	0.001	1.28 (1.10–1.49) ‡	0	-	-	*p* > 0.1	Weak
Unverzagt, et al. 2017	IFN-α monotherapy vs standard targeted therapy	Metastatic RCC	2	582/584	PFS	HR	2/0/0	<0.001	2.23 (1.79–2.76) ‡	0	-	-	*p* > 0.1	Weak
**Interleukin-2 and others**														
Roviello, et al. 2017	IL-2 + CTx vs CTx	Colorectal cancer	4	153/150	Objective response	RR	0/3/1	0.003	1.65 (1.19–2.28)	0	0.81–3.36	0.60	*p* > 0.1	Suggestive
Buyse, et al. 2011	IL-2 remission maintenance monotherapy vs obs	Acute myeloid leukemia	6	725/730	OS	HR	0/6/0	0.843	1.01 (0.95–1.06)	0	0.93–1.09	0.39	*p* > 0.1	No association
Hamm, et al. 2008	IL-2 + IFN-α + CTx vs CTx	Metastatic malignant melanoma	5	364/365	OS	HR	0/5/0	0.642	0.95 (0.78–1.16)	26	0.59–1.55	0.09	NA	No association
Hamm, et al. 2008	IL-2 + IFN-α + CTx vs CTx	Metastatic malignant melanoma	6	568/566	Overall response	RR	0/5/1	<0.001	1.52 (1.24–1.87)	0	1.14–2.04	0.19	*p* > 0.1	Suggestive
Ives, et al. 2007	IL-2 + IFN-α + CTx vs CTx	Metastatic melanoma	7	607/599	OS	OR	0/7/0	0.729	1.07 (0.74–1.55)	36	0.46–2.50	0.96	*p* > 0.1	No association
Ives, et al. 2007	IL-2 + IFN-α + CTx vs CTx	Metastatic melanoma	7	527/564	Overall response	OR	0/6/1	<0.001	0.58 (0.44–0.77)	33	0.40–0.84	0.17	*p* > 0.1	Suggestive
Hotte, et al. 2007	IL-2 based regimen + CTx vs CT or IFN	Unresectable or metastatic RCC	2	319/100	1-year mortality	RR	0/0/2	0.003	0.56 (0.38–0.82)	19	-	-	*p* > 0.1	Weak
Rossi, et al. 2010	IFN-γ maintenance or consolidation therapy vs *p* or obs	Small-cell lung cancer	2	116/111	OS	HR	0/2/0	0.538	1.09 (0.82–1.46)	0	-	-	*p* > 0.1	No association
**Cancer vaccine**														
Cao, et al. 2014	DC vs non-DC	High-grade glioma	3	44/42	2-year OS	OR	0/3/0	0.038	3.41 (1.07–10.81)	0	-	-	*p* > 0.1	Weak
Kawalec, et al. 2012	Sipuleucel-T vs *p*	Castration-resistant prostate cancer	3	488/249	OS	HR	0/1/2	<0.001	0.73 (0.60–0.88)	0	0.22–2.44	0.71	*p* > 0.1	Suggestive
Ding, et al. 2014	VAX (Tecemotide or EGF vaccine or SRL172 or TG4010 or MAGE-A3 or L-BLP25) vs CTx or obs or α-tocopherol	NSCLC	6	1363/876	OS	OR	0/4/2	0.001	0.56 (0.39–0.79)	65	0.34–0.92	0.99	*p* > 0.1	Weak
Yu, et al. 2017	VAX vs CTx or *p*	Advanced NSCLC	3	1014/595	PFS	OR	0/1/2	0.015	1.31 (1.05–1.63)	49	0.32–5.33	0.62	*p* > 0.1	Suggestive
Zhou, et al. 2016	VAX (EGF vaccine or BLP-25 or TG4010, etc) vs P	Advanced NSCLC	5	1314/884	OS	HR	0/4/1	0.002	0.83 (0.74–0.93)	17	0.64–1.07	0.20	*p* > 0.1	Suggestive
Zhou, et al. 2016	VAX (EGF vaccine or BLP-25 or TG4010, etc) vs CTx	Advanced NSCLC	4	300/302	OS	HR	0/4/0	0.012	0.77 (0.63–0.94)	0	0.50–1.20	0.55	*p* > 0.1	Weak
Bai, et al. 2017	Postsurgical AIT (IL-2 + IFN-α or IFN-α2b or IFN-α, etc) vs no AT	Locally advanced RCC	5	941/902	OS	HR	0/5/0	0.345	1.08 (0.92–1.28)	0	0.83–1.42	0.32	NA	No association

Abbreviations: RCT, randomized controlled trial; *N*., number; SE, standard effect; CI, confidence interval; M, model; F, fixed effect; R, random effect; NA, not available; C, concordance with largest study; Y, concordant with largest study; *n*, not concordant with largest study; OS, overall survival; PFS progression-free survival; RR, risk ratio; HR, hazard ratio; OR, odds ratio; IFN, interferon; 5FU, fluorouracil; CTx, chemotherapy; CT, conventional therapy; obs, observation; IL-2, Interleukin-2; DC, dendritic cell based vaccine; *p*, placebo; VAX, vaccine; EGF, Epidermal Growth Factor; HCC, hepatocellular carcinoma; NSCLC, non-small cell lung cancer; RCC, renal cell cancer. † Number of individual studies of effect size with statistically significant in direction in favor of control therapy/not statistically significant/statistically significant in favor of immunotherapy. ‡ The control therapy was favored over the experimental therapy.

## Supplementary Materials

The following are available online at https://www.mdpi.com/2072-6694/11/11/1801/s1. Figure S1: Flow chart of literature search, Figure S2: (A) Effect size and level of evidence reported in meta-analyses of cancer therapeutic anti-PD-1/PD-L1 monoclonal antibodies. (B) Effect size and level of evidence reported in meta-analyses of cancer therapeutic anti-CD20 monoclonal antibodies. Solid horizontal lines represent risk ratio or hazard ratio; dotted horizontal lines represent odds ratio, Figure S3: (A) Effect size and level of evidence reported in meta-analyses of cancer therapeutic DC/CIK. (B) Effect size and level of evidence reported in meta-analyses of cancer therapeutic CIK. (C) Effect size and level of evidence reported in meta-analyses of adoptive cell immunotherapy for cancer. Solid horizontal lines represent risk ratio or hazard ratio; dotted horizontal lines represent odds ratio, Figure S4: (A) Effect size and level of evidence reported in meta-analyses of cancer therapeutic interferon-α. (B) Effect size and level of evidence reported in meta-analyses of cancer therapeutic interleukin-2. (C) Effect size and level of evidence reported in meta-analyses of cancer vaccines. Solid horizontal lines represent risk ratio or hazard ratio; dotted horizontal lines represent odds ratio, Figure S5: Anti-PD-1/PD-L1 mAb treatment on solid tumor, OS, Figure S6: Anti-PD-1/PD-L1 mAb treatment on solid tumor, PFS, Figure S7: Treatment with DC/CIK on NSCLC, OS, Figure S8: Level of evidence algorithm, Table S1: Descriptive statistics of meta-analyses according to immunotherapy category, Table S2: Umbrella review summary and level of evidence reported in meta-analyses of cancer therapeutic anti-PD-1/PD-L1 monoclonal antibodies, Table S3: Umbrella review summary and level of evidence reported in meta-analyses of cancer therapeutic anti-CD20 monoclonal antibodies, Table S4: Umbrella review summary and level of evidence reported in meta-analyses of adoptive cell immunotherapy for cancer, Table S5: Umbrella review summary and level of evidence reported in meta-analyses of cancer therapeutic immunomodulatory cytokines, Table S6: Umbrella review summary and level of evidence reported in meta-analyses of cancer therapeutic vaccines, Table S7: Umbrella review summary and level of evidence reported in meta-analyses of uncategorized immunotherapy, Table S8: Comparison between re-analyses of RCTs and re-analyses of RCTs plus non-RCT(s), Table S9: PRISMA Checklist; Table S10: Quality of the individual studies in the eligible meta-analyses using the Cochrane risk of bias tool; Reference list of the eligible articles.
