# Supplementary material for: Efficacy of Cancer Immunotherapy: An Umbrella Review of Meta-Analyses of Randomized Controlled Trials
Source: Cancers (Basel). 2019 Nov 15;11(11):1801. doi: 10.3390/cancers11111801 (PMC6895783; doi:10.3390/cancers11111801)
Supplement: Supplementary file 1 [file cancers-11-01801-s001.pdf]

Supplementary Materials

Efficacy of Cancer Immunotherapy: An Umbrella Review of Meta-analyses of Randomized Controlled Trials

Jong Yeob Kim, Keum Hwa Lee, Michael Eisenhut, Hans J. van der Vliet, Andreas Kronbichler, Gwang Hun Jeong, Jae Il Shin and Gabriele Gamerith

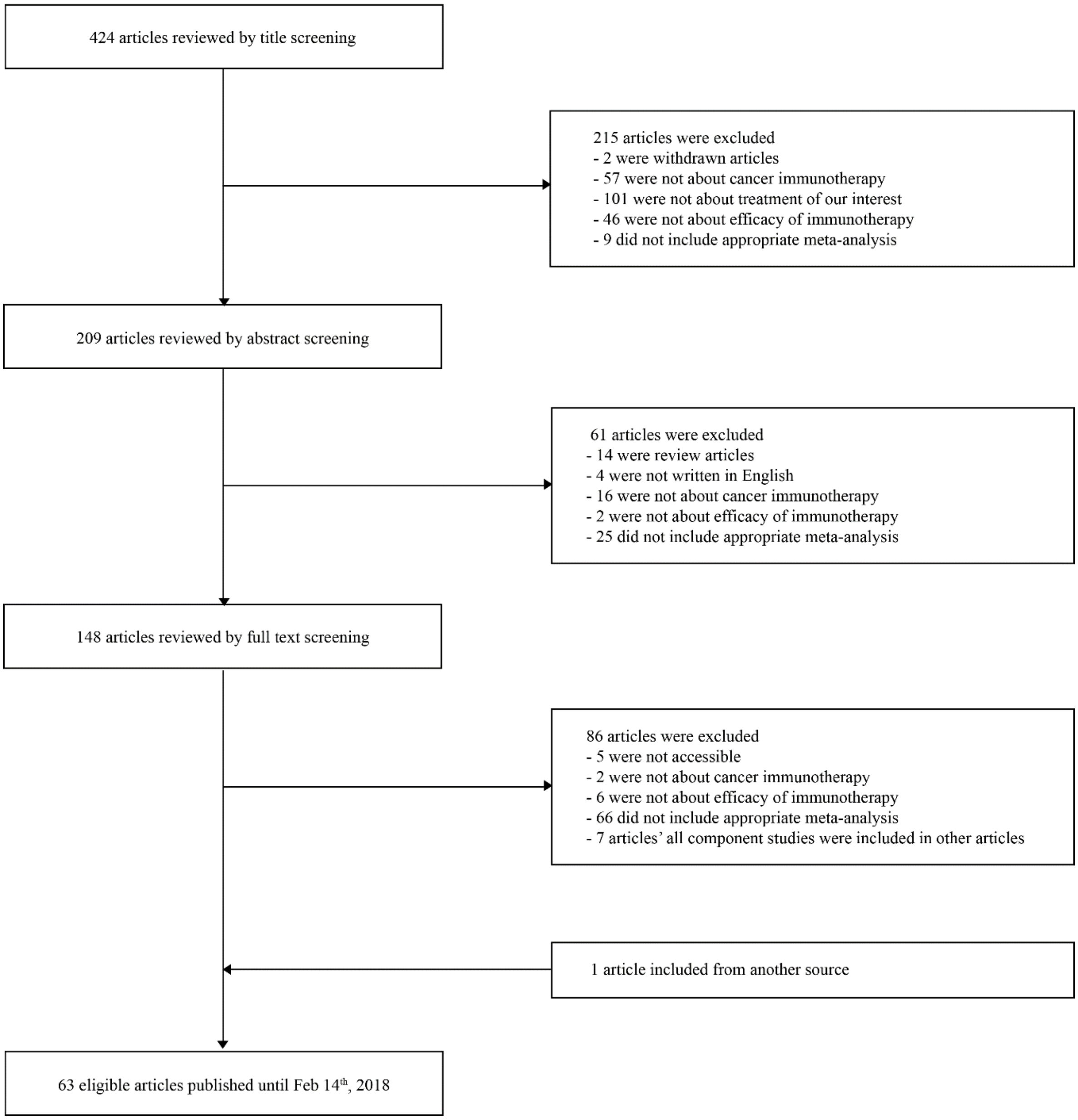

Figure S1. Flow chart of literature search.

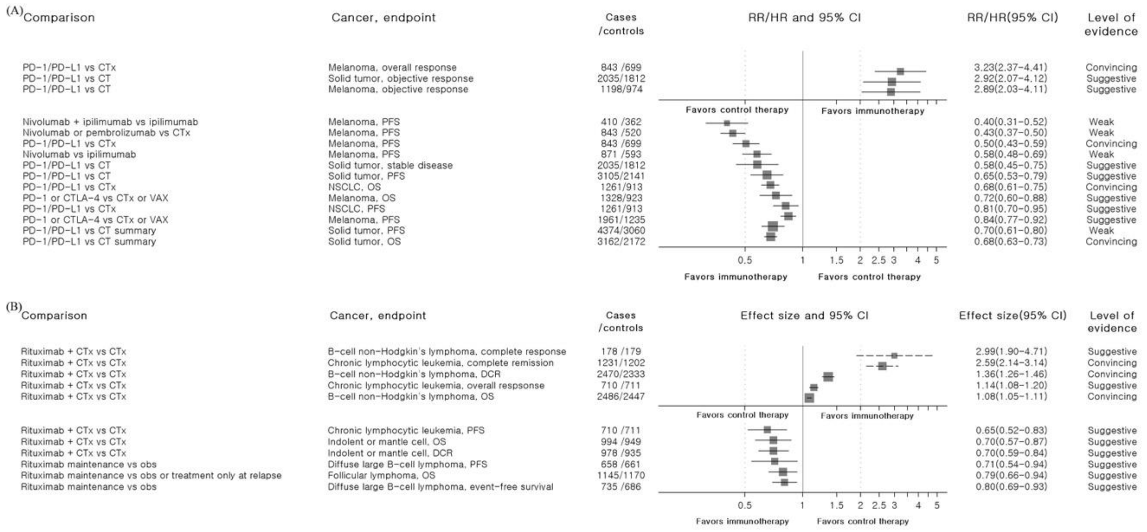

Figure S2. (**A**) Effect size and level of evidence reported in meta-analyses of cancer therapeutic anti-PD-1/PD-L1 monoclonal antibodies. (**B**) Effect size and level of evidence reported in meta-analyses of cancer therapeutic anti-CD20 monoclonal antibodies. Solid horizontal lines represent risk ratio or hazard ratio; dotted horizontal lines represent odds ratio.

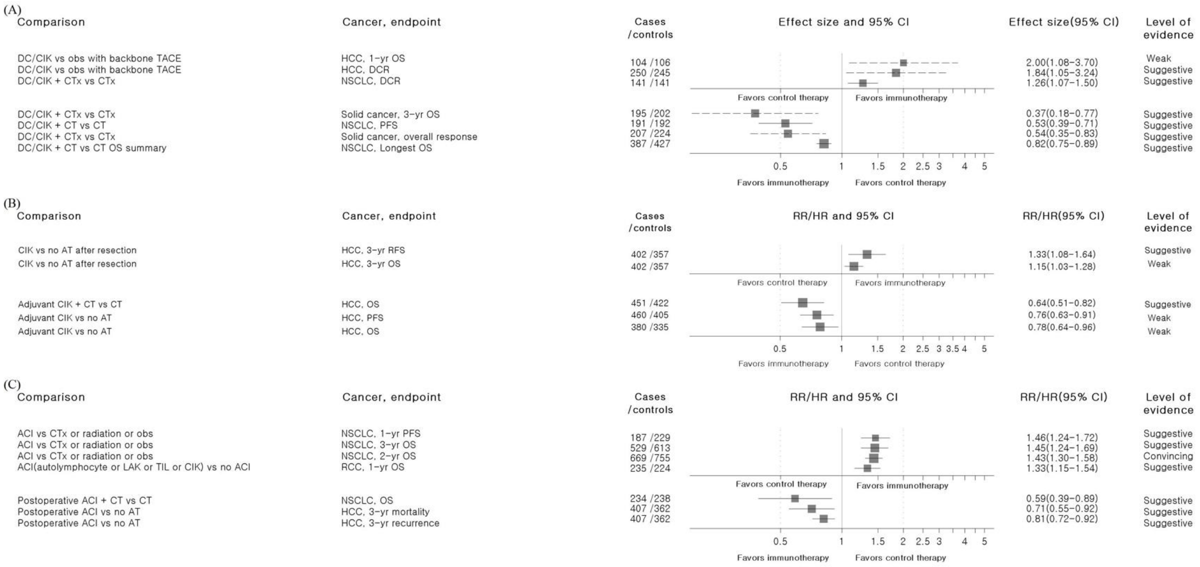

Figure S3. (**A**) Effect size and level of evidence reported in meta-analyses of cancer therapeutic DC/CIK (dendritic cells/ cytokine-induced killer cells). (**B**) Effect size and level of evidence reported in meta-analyses of cancer therapeutic CIK. (**C**) 5. Effect size and level of evidence reported in meta-analyses of adoptive cell immunotherapy for cancer. Solid horizontal lines represent risk ratio or hazard ratio; dotted horizontal lines represent odds ratio.

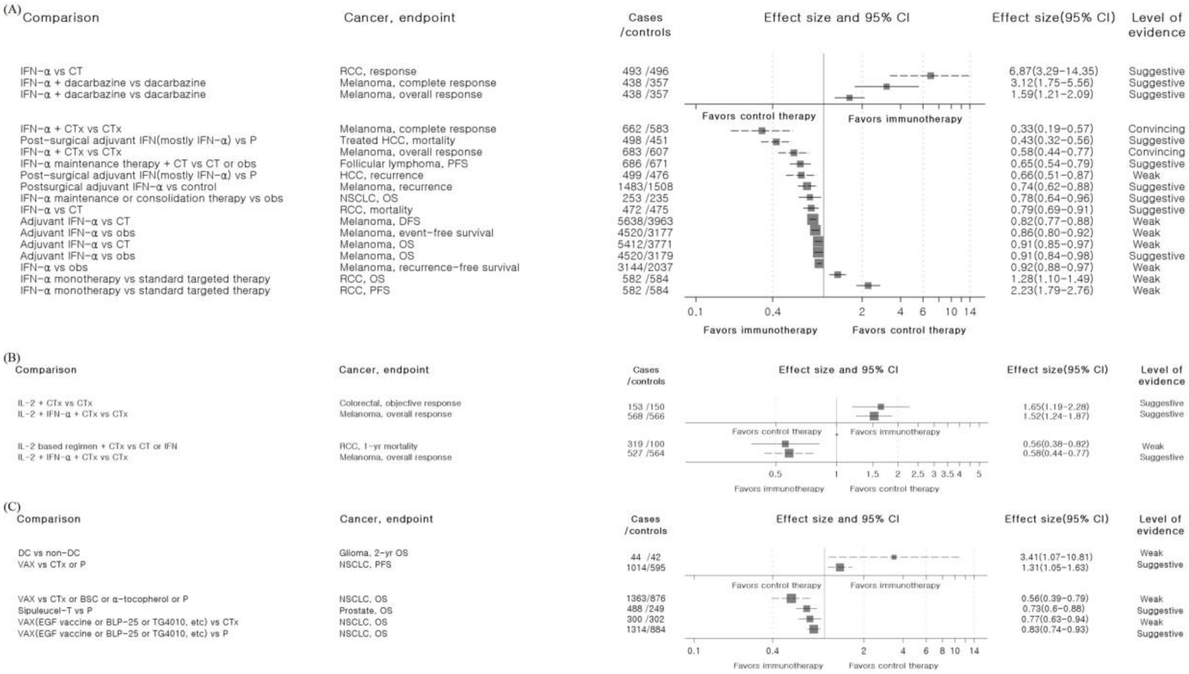

Figure S4. (**A**) Effect size and level of evidence reported in meta-analyses of cancer therapeutic interferon-α. (**B**) Effect size and level of evidence reported in meta-analyses of cancer therapeutic interleukin-2. (**C**) Effect size and level of evidence reported in meta-analyses of cancer vaccines. Solid horizontal lines represent risk ratio or hazard ratio; dotted horizontal lines represent odds ratio.

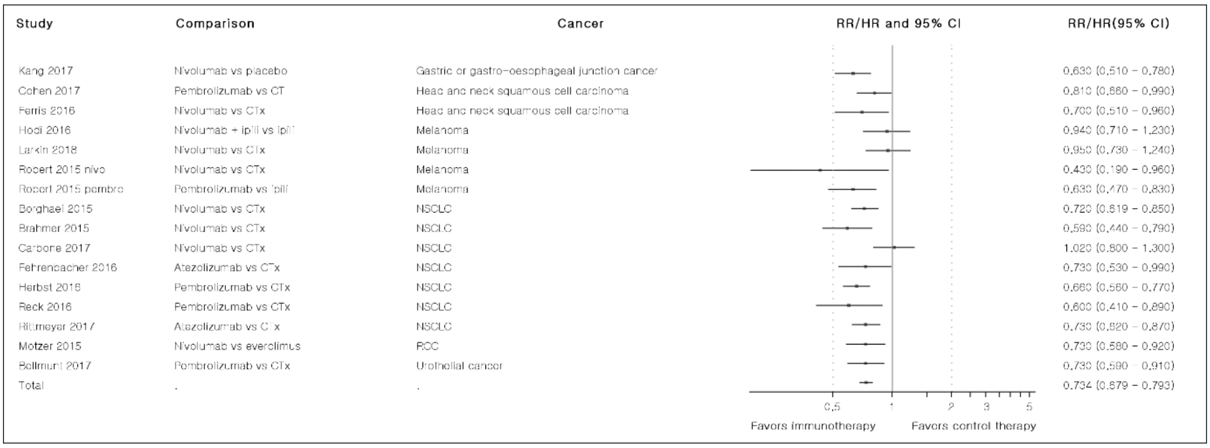

Figure S5. Anti-PD-1/PD-L1 mAb treatment on solid tumor, Overall Survival (OS).

References

1. Bellmunt J, de Wit R, Vaughn DJ, et al. Pembrolizumab as Second-Line Therapy for Advanced Urothelial Carcinoma. N Engl J Med. 2017;376(11):1015-1026. doi:10.1056/NEJMoa1613683
2. Borghaei H, Paz-Ares L, Horn L, et al. Nivolumab versus Docetaxel in Advanced Nonsquamous Non-Small-Cell Lung Cancer. N Engl J Med. 2015;373(17):1627-1639. doi:10.1056/NEJMoa1507643
3. Brahmer J, Reckamp KL, Baas P, et al. Nivolumab versus Docetaxel in Advanced Squamous-Cell Non–Small-Cell Lung Cancer. N Engl J Med. 2015;373(2):123-135. doi:10.1056/NEJMoa1504627
4. Carbone DP, Reck M, Paz-Ares L, et al. First-Line Nivolumab in Stage IV or Recurrent Non-Small-Cell Lung Cancer. N Engl J Med. 2017;376(25):2415-2426. doi:10.1056/NEJMoa1613493
5. Cohen EE, Harrington KJ, Tourneau C Le, Dinis J, Licitra L, Ahn M-J. Pembrolizumab (pembro) vs standard of care (SOC) for recurrent or metastatic head and neck squamous cell carcinoma (R/M HNSCC): Phase 3 KEYNOTE-040 trial. Nejm. 2001;345(26):1890-1900. doi:10.1200/jco.2015.33.15

6. Fehrenbacher L, Spira A, Ballinger M, et al. Atezolizumab versus docetaxel for patients with previously treated non-small-cell lung cancer (POPLAR): a multicentre, open-label, phase 2 randomised controlled trial. Lancet (London, England). 2016;387(10030):1837-1846. doi:10.1016/S0140-6736(16)00587-0
7. Ferris RL, Blumenschein G, Fayette J, et al. Nivolumab for Recurrent Squamous-Cell Carcinoma of the Head and Neck. N Engl J Med. 2016;375(19):1856-1867. doi:10.1056/NEJMoa1602252
8. Herbst RS, Baas P, Kim D-W, et al. Pembrolizumab versus docetaxel for previously treated, PD-L1-positive, advanced non-small-cell lung cancer (KEYNOTE-010): a randomised controlled trial. Lancet (London, England). 2016;387(10027):1540-1550. doi:10.1016/S0140-6736(15)01281-7
9. Hodi FS, Chesney J, Pavlick AC, et al. Combined nivolumab and ipilimumab versus ipilimumab alone in patients with advanced melanoma: 2-year overall survival outcomes in a multicentre, randomised, controlled, phase 2 trial. Lancet Oncol. 2016;17(11):1558-1568. doi:10.1016/S1470-2045(16)30366-7
10. Kang Y-K, Boku N, Satoh T, et al. Nivolumab in patients with advanced gastric or gastro-oesophageal junction cancer refractory to, or intolerant of, at least two previous chemotherapy regimens (ONO-4538-12, ATTRACTION-2): a randomised, double-blind, placebo-controlled, phase 3 trial. Lancet (London, England). 2017;390(10111):2461-2471. doi:10.1016/S0140-6736(17)31827-5
11. Larkin J, Minor D, D'Angelo S, et al. Overall Survival in Patients With Advanced Melanoma Who Received Nivolumab Versus Investigator's Choice Chemotherapy in CheckMate 037: A Randomized, Controlled, Open-Label Phase III Trial. J Clin Oncol. 2018;36(4):383-390. doi:10.1200/JCO.2016.71.8023
12. Motzer RJ, Escudier B, McDermott DF, et al. Nivolumab versus Everolimus in Advanced Renal-Cell Carcinoma. N Engl J Med. 2015;373(19):1803-1813. doi:10.1056/NEJMoa1510665
13. Reck M, Rodríguez-Abreu D, Robinson AG, et al. Pembrolizumab versus Chemotherapy for PD-L1–Positive Non–Small-Cell Lung Cancer. N Engl J Med. 2016;375(19):1823-1833. doi:10.1056/NEJMoa1606774
14. Rittmeyer A, Barlesi F, Waterkamp D, et al. Atezolizumab versus docetaxel in patients with previously treated non-small-cell lung cancer (OAK): a phase 3, open-label, multicentre randomised controlled trial. Lancet. 2017;389(10066):255-265. doi:10.1016/S0140-6736(16)32517-X
15. Robert C, Long G V., Brady B, et al. Nivolumab in Previously Untreated Melanoma without BRAF Mutation. N Engl J Med. 2015;372(4):320-330. doi:10.1056/NEJMoa1412082

Robert C, Schachter J, Long G V., et al. Pembrolizumab versus Ipilimumab in Advanced Melanoma. N Engl J Med. 2015;372(26):2521-2532. doi:10.1056/NEJMoa1503093

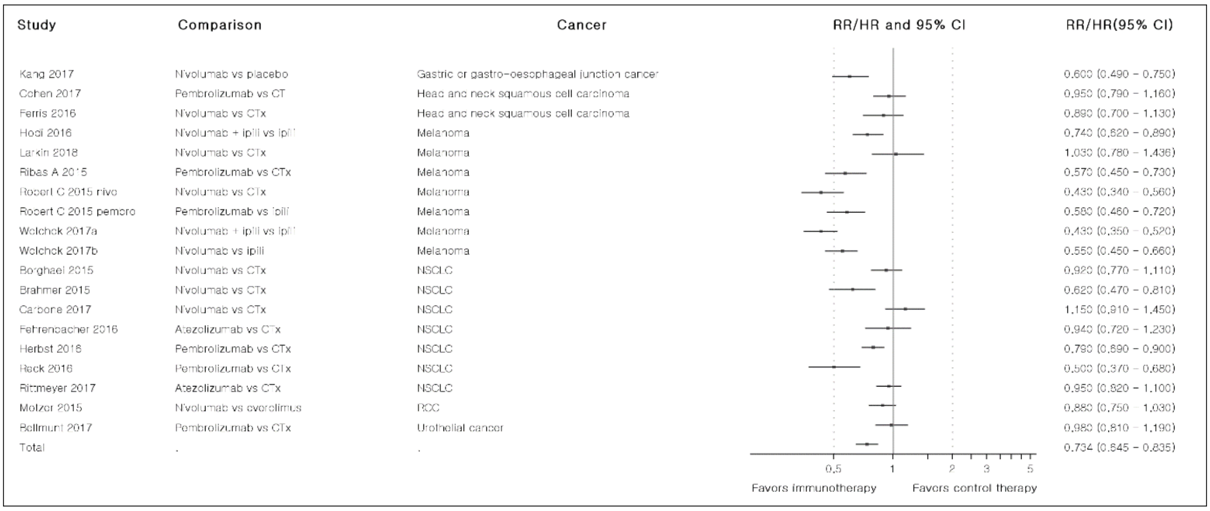

Figure S6. Anti-PD-1/PD-L1 mAb treatment on solid tumor, Progression-Free Survival (PFS)

16. Bellmunt J, de Wit R, Vaughn DJ, et al. Pembrolizumab as Second-Line Therapy for Advanced Urothelial Carcinoma. N Engl J Med. 2017;376(11):1015-1026. doi:10.1056/NEJMoa1613683
17. Borghaei H, Paz-Ares L, Horn L, et al. Nivolumab versus Docetaxel in Advanced Nonsquamous Non-Small-Cell Lung Cancer. N Engl J Med. 2015;373(17):1627-1639. doi:10.1056/NEJMoa1507643
18. Brahmer J, Reckamp KL, Baas P, et al. Nivolumab versus Docetaxel in Advanced Squamous-Cell Non–Small-Cell Lung Cancer. N Engl J Med. 2015;373(2):123-135. doi:10.1056/NEJMoa1504627
19. Carbone DP, Reck M, Paz-Ares L, et al. First-Line Nivolumab in Stage IV or Recurrent Non-Small-Cell Lung Cancer. N Engl J Med. 2017;376(25):2415-2426. doi:10.1056/NEJMoa1613493
20. Cohen EE, Harrington KJ, Tourneau C Le, Dinis J, Licitra L, Ahn M-J. Pembrolizumab (pembro) vs standard of care (SOC) for recurrent or metastatic head and neck squamous cell carcinoma (R/M HNSCC): Phase 3 KEYNOTE-040 trial. Nejm. 2001;345(26):1890-1900. doi:10.1200/jco.2015.33.15
21. Fehrenbacher L, Spira A, Ballinger M, et al. Atezolizumab versus docetaxel for patients with previously treated non-small-cell lung cancer (POPLAR): a multicentre, open-label, phase 2 randomised controlled trial. Lancet (London, England). 2016;387(10030):1837-1846. doi:10.1016/S0140-6736(16)00587-0
22. Ferris RL, Blumenschein G, Fayette J, et al. Nivolumab for Recurrent Squamous-Cell Carcinoma of the Head and Neck. N Engl J Med. 2016;375(19):1856-1867. doi:10.1056/NEJMoa1602252
23. Herbst RS, Baas P, Kim D-W, et al. Pembrolizumab versus docetaxel for previously treated, PD-L1-positive, advanced non-small-cell lung cancer (KEYNOTE-010): a randomised controlled trial. Lancet (London, England). 2016;387(10027):1540-1550. doi:10.1016/S0140-6736(15)01281-7
24. Hodi FS, Chesney J, Pavlick AC, et al. Combined nivolumab and ipilimumab versus ipilimumab alone in patients with advanced melanoma: 2-year overall survival outcomes in a multicentre, randomised, controlled, phase 2 trial. Lancet Oncol. 2016;17(11):1558-1568. doi:10.1016/S1470-2045(16)30366-7
25. Kang Y-K, Boku N, Satoh T, et al. Nivolumab in patients with advanced gastric or gastro-oesophageal junction cancer refractory to, or intolerant of, at least two previous chemotherapy regimens (ONO-4538-12, ATTRACTION-2): a randomised, double-blind, placebo-controlled, phase 3 trial. Lancet (London, England). 2017;390(10111):2461-2471. doi:10.1016/S0140-6736(17)31827-5
26. Larkin J, Minor D, D'Angelo S, et al. Overall Survival in Patients With Advanced Melanoma Who Received Nivolumab Versus Investigator's Choice Chemotherapy in CheckMate 037: A Randomized, Controlled, Open-Label Phase III Trial. J Clin Oncol. 2018;36(4):383-390. doi:10.1200/JCO.2016.71.8023
27. Motzer RJ, Escudier B, McDermott DF, et al. Nivolumab versus Everolimus in Advanced Renal-Cell Carcinoma. N Engl J Med. 2015;373(19):1803-1813. doi:10.1056/NEJMoa1510665
28. Reck M, Rodríguez-Abreu D, Robinson AG, et al. Pembrolizumab versus Chemotherapy for PD-L1–Positive Non–Small-Cell Lung Cancer. N Engl J Med. 2016;375(19):1823-1833. doi:10.1056/NEJMoa1606774
29. Ribas A, Puzanov I, Dummer R, et al. Pembrolizumab versus investigator-choice chemotherapy for ipilimumab-refractory melanoma (KEYNOTE-002): a randomised, controlled, phase 2 trial. Lancet Oncol. 2015;16(8):908-918. doi:10.1016/S1470-2045(15)00083-2

30. Rittmeyer A, Barlesi F, Waterkamp D, et al. Atezolizumab versus docetaxel in patients with previously treated non-small-cell lung cancer (OAK): a phase 3, open-label, multicentre randomised controlled trial. Lancet. 2017;389(10066):255-265. doi:10.1016/S0140-6736(16)32517-X
31. Robert C, Long G V., Brady B, et al. Nivolumab in Previously Untreated Melanoma without BRAF Mutation. N Engl J Med. 2015;372(4):320-330. doi:10.1056/NEJMoa1412082
32. Robert C, Schachter J, Long G V., et al. Pembrolizumab versus Ipilimumab in Advanced Melanoma. N Engl J Med. 2015;372(26):2521-2532. doi:10.1056/NEJMoa1503093
33. Wolchok JD, Chiarion-Sileni V, Gonzalez R, et al. Overall Survival with Combined Nivolumab and Ipilimumab in Advanced Melanoma. N Engl J Med. 2017;377(14):1345-1356. doi:10.1056/NEJMoa1709684.

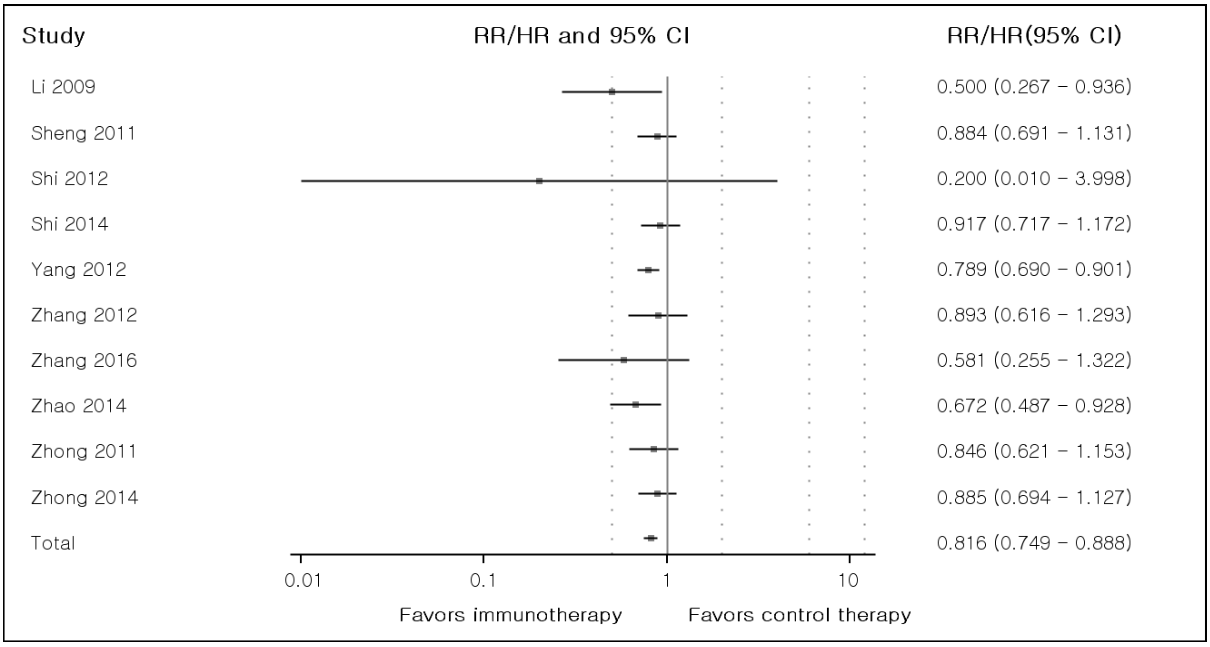

Figure S7. Treatment with DC/CIK on Non-Small Cell Lung Cancer (NSCLC), OS

34. Li H, Wang C, Yu J, et al. Dendritic cell-activated cytokine-induced killer cells enhance the anti-tumor effect of chemotherapy on non-small cell lung cancer in patients after surgery. Cytotherapy. 2009;11(8):1076-1083. doi:10.3109/14653240903121252
35. Sheng C, Bao F, Xu S. Clinical research on chemotherapy combined with dendritic cell-cytokine killer cells for non-small cell lung cancer. J Pr Oncol. 2011;26(5):503-506.
36. Shi S Bin, Ma TH, Li CH, Tang XY. Effect of maintenance therapy with dendritic cells: cytokine-induced killer cells in patients with advanced non-small cell lung cancer. Tumori. 98(3):314-319. doi:10.1700/1125.12398
37. Shi S-B, Tang X-Y, Tian J, Chang C-X, Li P, Qi J-L. Efficacy of erlotinib plus dendritic cells and cytokine-induced killer cells in maintenance therapy of advanced non-small cell lung cancer. J Immunother. 2014;37(4):250-255. doi:10.1097/CJI.0000000000000015
38. Wu C, Jiang J, Shi L, Xu N. Prospective study of chemotherapy in combination with cytokine-induced killer cells in patients suffering from advanced non-small cell lung cancer. Anticancer Res. 28(6B):3997-4002. http://www.ncbi.nlm.nih.gov/pubmed/19192663. Accessed May 29, 2018.
39. Yang L, Ren B, Li H, et al. Enhanced antitumor effects of DC-activated CIKs to chemotherapy treatment in a single cohort of advanced non-small-cell lung cancer patients. Cancer Immunol Immunother. 2013;62(1):65-73. doi:10.1007/s00262-012-1311-8
40. Zhang J, Geng J, Han Z. Clinical effects of treatment of dendritic cells combined with cytokine induced killer cells therapy in patients with advanced colon carcinoma. Acta Acad Med Xuzhou. 2011;31(7):457-459.
41. Zhang J, Mao G, Han Y, et al. The clinical effects of DC-CIK cells combined with chemotherapy in the treatment of advanced NSCLC. Chinese-German J Clin Oncol. 2012;11(2):67-71. doi:DOI 10.1007/s10330-011-0929-x

42. Zhao M, Li H, Li L, Zhang Y. Effects of a gemcitabine plus platinum regimen combined with a dendritic cell-cytokine induced killer immunotherapy on recurrence and survival rate of non-small cell lung cancer patients. Exp Ther Med. 2014;7(5):1403-1407. doi:10.3892/etm.2014.1574
43. Zhong R, Teng J, Han B. Dendritic cells combining with cytokine-induced killer cells synergize chemotherapy in patients with late-stage non-small cell lung cancer. Cancer Immunol Immun. 2011;60(10):1497-1502.

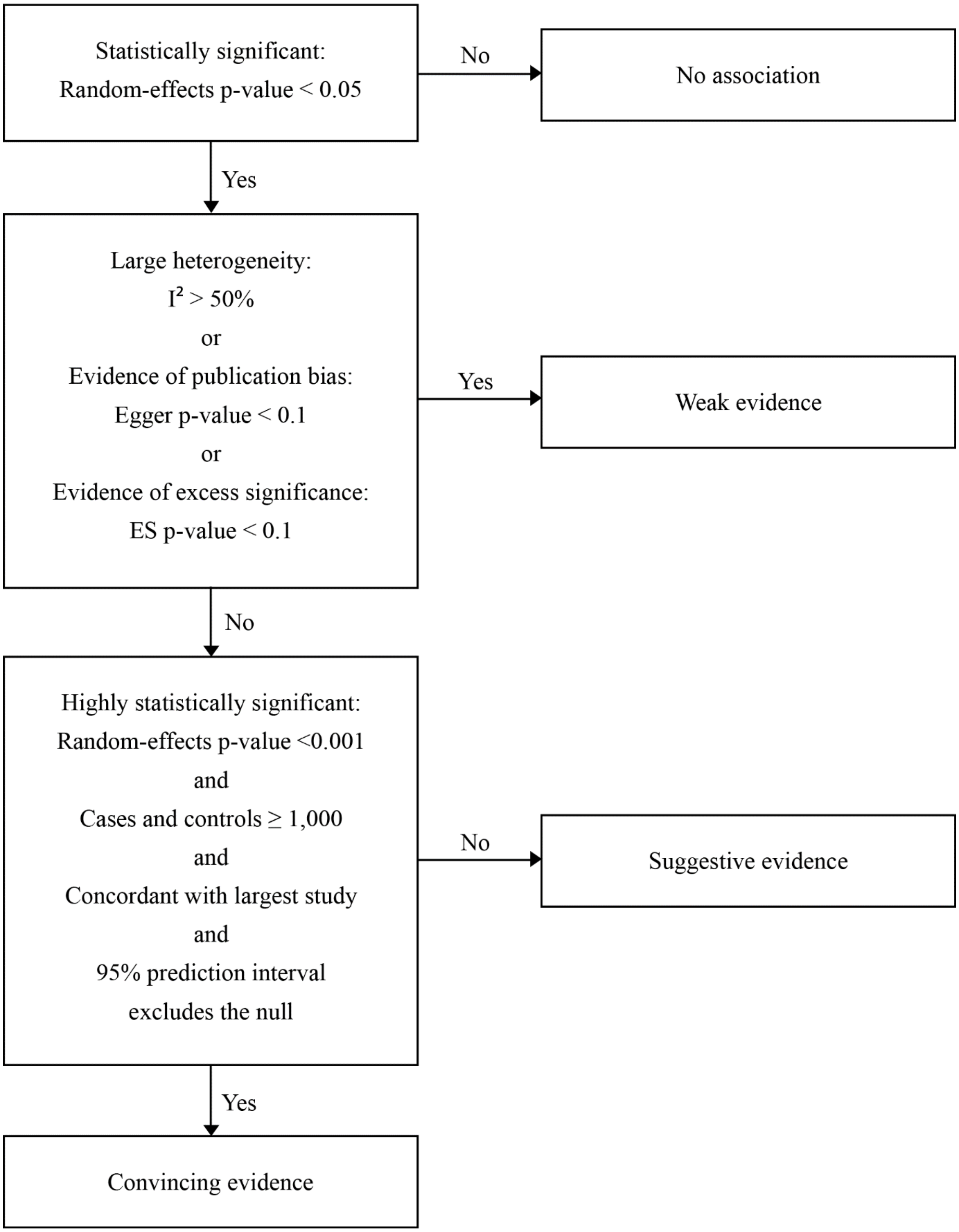

Figure S8. Level of evidence algorithm

*When a meta-analysis had no evidence of publication bias or ES but had high in-between study heterogeneity ($I^2 > 50$), we rechecked the results of its component studies to find out whether high heterogeneity was due to the differences in the direction of effects or due to the differences in the size of the associations. When the number of statistically significant component studies was the same or greater than the number of studies which were not significant or significant in the opposite direction, the comparison was classified as suggestive evidence, or convincing evidence if further criteria were met.

*When no statistically significant component study was observed in a meta-analysis, the comparison was at best classified as weak evidence, even in the absence of biases.

Table S1. Descriptive statistics of meta-analyses according to immunotherapy category.

Statistics / Immunotherapy category		Number of articles	Number of meta-analyses	Individual component studies N.	Median	IQR	Cases plus controls N.	Median	IQR†	Random effects *p-Value* <0.001	<0.05	>0.05	Heterogeneity Low or moderate	Large	Publication bias No evidence	Evidence detected	Cases plus controls > = 1000	<1000	Concordance with largest study Concordant	Not concordant	Excess significance No evidence	Evidence detected or NA	95% prediction interval Exclude the null	Included the null or NA	Level of evidence Convincing	Suggestive	Weak	No association
mAB	Anti-PD-1/PD-L1	6	29	129	3	2-6	61,006	1,542	1,141-3,196	17(59%)	5(17%)	7(24%)	14(48%)	15(52%)	19(66%)	10(34%)	24(83%)	5(17%)	21(72%)	8(28%)	23(79%)	6(21%)	4(14%)	25(86%)	3(10%)	11(38%)	8(28%)	7(24%)
	Anti-CD20	9	28	155	4	3-7	45,573	1,421	488-1,936	16(57%)	6(21%)	6(21%)	17(61%)	11(39%)	22(79%)	6(21%)	19(68%)	9(32%)	13(46%)	15(54%)	22(79%)	6(21%)	5(18%)	23(82%)	3(11%)	11(39%)	8(29%)	6(21%)
ACI	DC/CIK	6	34	125	4	2-5	9,737	282	174-371	5(15%)	20(59%)	9(26%)	33(97%)	1(3%)	22(65%)	12(35%)	0(0%)	34(100%)	11(32%)	23(68%)	31(91%)	3(9%)	5(15%)	29(85%)	0(0%)	13(38%)	12(35%)	9(26%)
	CIK	3	14	60	4	3-5	9,199	639.5	552-766	5(36%)	5(36%)	4(29%)	12(86%)	2(14%)	12(86%)	2(14%)	0(0%)	14(100%)	8(57%)	6(43%)	14(100%)	0(0%)	0(0%)	14(100%)	0(0%)	7(50%)	3(21%)	4(29%)
	Others or not specified	6	30	153	4	2.75-6	15,581	371	261-769	6(20%)	15(50%)	9(30%)	27(90%)	3(10%)	21(70%)	9(30%)	3(10%)	27(90%)	8(27%)	22(73%)	27(90%)	3(10%)	8(27%)	22(73%)	1(3%)	13(43%)	7(23%)	9(30%)
Cytokine	IFN-α	15	44	367	8	5.25-11.75	90,619	1,254	948-1,661	14(32%)	9(20%)	21(48%)	39(89%)	5(11%)	30(68%)	14(32%)	29(66%)	15(34%)	10(23%)	34(77%)	41(93%)	3(7%)	13(30%)	31(70%)	2(5%)	10(23%)	11(25%)	21(48%)
	IL-2	7	19	101	6	4-7	17,549	903	679-1,206	4(21%)	2(11%)	13(68%)	19(100%)	0(0%)	15(79%)	4(21%)	9(47%)	10(53%)	2(11%)	17(89%)	15(79%)	4(21%)	4(21%)	15(79%)	0(0%)	3(16%)	3(16%)	13(68%)
	Others or not specified	3	8	30	4	2.25-5	7,075	1,007	380-1,224	0(0%)	0(0%)	8(100%)	7(88%)	1(13%)	6(75%)	2(25%)	4(50%)	4(50%)	0(0%)	8(100%)	3(38%)	5(63%)	0(0%)	8(100%)	0(0%)	0(0%)	0(0%)	8(100%)
Vaccine	DC	2	5	14	3	2.5-3	1,694	86	67-737	1(20%)	1(20%)	3(60%)	5(100%)	0(0%)	3(60%)	2(40%)	0(0%)	5(100%)	1(20%)	4(80%)	4(80%)	1(20%)	0(0%)	5(100%)	0(0%)	1(20%)	1(20%)	3(60%)
	Others or not specified	7	14	46	2.5	2-4.25	18,324	1,071	848-1,756	1(7%)	5(36%)	8(57%)	9(64%)	5(36%)	5(36%)	9(64%)	9(64%)	5(36%)	2(14%)	12(86%)	11(79%)	3(21%)	1(7%)	13(93%)	0(0%)	2(14%)	4(29%)	8(57%)
Uncategorized immunotherapy		7	22	126	5	3-9	48,499	2,342	1,066-2,736	2(9%)	7(32%)	13(59%)	13(59%)	9(41%)	15(68%)	7(32%)	17(77%)	5(23%)	5(23%)	17(77%)	10(45%)	12(55%)	3(14%)	19(86%)	0(0%)	4(18%)	5(23%)	13(59%)
**Analyses of RCTs TOTAL**		**63**	**247**	**1,306**	**4**	**3-7**	**324,856**	**885**	**410-1,542**	**71(29%)**	**75(30%)**	**101(41%)**	**195(79%)**	**52(21%)**	**170(69%)**	**77(31%)**	**114(46%)**	**133(54%)**	**81(33%)**	**166(67%)**	**201(81%)**	**46(19%)**	**43(17%)**	**204(83%)**	**9(4%)**	**75(30%)**	**62(25%)**	**101(41%)**
Analyses of RCTs plus non-RCTs		6	35	288	-	-	34,326	-	-	-	-	-	-	-	-	-	-	-	-	-	-	-	-	-	-	-	-	-

Abbreviations: *N.*, number; IQR, interquartile range; NA, not available; mAB, monoclonal antibody; ACI, adoptive cell immunotherapy; DC/CIK, dendritic cells with cytokine-induced killer cells; CIK, cytokine-induced killer cells; IFN-*α*, interferon alpha; IL-2, interleukin-2; DC, dendritic cells; IT, immunotherapy; RCT, randomized controlled trials. * 4 results from one article showed weak evidence in favor of the control therapy. † Value is rounded to the nearest unit.

Table S2. Umbrella review summary and level of evidence reported in meta-analyses of cancer therapeutic anti-PD-1/PD-L1 monoclonal antibodies.

Author, year	Comparison	Cancer type	RCT *N.*	Intervention/control	Outcome	Metrics	M *	Reported *p-Value* *	Reported SE (95% CI) *	Reported I2(%) (*p*-Value) *	R/N/S †	F *p-Value* §	F SE (95% CI) §	R *p-Value* §	R SE (95% CI) §	C ‡	I2(%) (*p-Value*) §	95% Prediction Interval	Egger *p*-Value	Excess Significance	Level of Evidence
**Melanoma**																					
Guan et al. 2016	Anti-PD-1/PD-L1 vs CTx	Advanced melanoma	3(4)	843/699	PFS	HR	F	<0.001	0.50(0.44-0.58)	16.9(0.307)	0/1/3	<0.001	0.50(0.44-0.58)	<0.001	0.50(0.43-0.59)	Y	17.91(0.301)	(0.32-0.80)	**0.24**	*p* > 0.1	Convincing
			3(4)	843/699	Overall response	RR	F	<0.001	3.42(2.49-4.69)	0.0(0.502)	0/0/4	<0.001	3.23(2.37-4.41)	<0.001	3.23(2.37-4.41)	Y	0(0.526)	(1.64-6.39)	**0.19**	*p* > 0.1	Convincing

Author, year	Comparison	Cancer type	RCT N.	Intervention/control	Outcome	Metrics	M*	Reported *p-Value* *	Reported SE (95% CI) *	Reported I2(%) (*p*-Value) *	R/N/S †	F *p-Value* §	F SE (95% CI) §	R *p-Value* §	R SE (95% CI) §	C ‡	I2(%) (*p-Value*) §	95% Prediction Interval	Egger *p*-Value	Excess Significance	Level of Evidence
Hao et al. 2017	Nivolumab vs ipilimumab	Metastatic advanced melanoma	2	871/593	1-year OS	RR	R	NA	1.37(1.03-1.74)	85.8(0.008)	0/0/2	<0.001	1.35(1.24-1.48)	0.008	1.37(1.09-1.73)	Y	85.24(0.009)	-	-	NA	Weak
			2	871/593	PFS	HR	F	NA	0.58(0.48-0.69)	0(0.925)	0/0/2	<0.001	0.58(0.48-0.69)	<0.001	0.58(0.48-0.69)	Y	0(0.925)	-	-	*p* > 0.1	Weak
			2	871/593	Objective response	RR	F	NA	2.51(2.03-3.09)	0(0.352)	0/0/2	<0.001	2.46(2.00-3.03)	<0.001	2.46(2.00-3.03)	Y	0(0.358)	-	-	NA	Weak
	Nivolumab + ipilimumab vs ipilimumab	Metastatic advanced melanoma	2	410/362	1-year OS	RR	R	NA	1.54(0.90-2.63)	93.5(0.000)	0/1/1	<0.001	1.80(1.61-2.00)	0.122	1.53(0.89-2.64)	N	93.47 (< 0.001)	-	-	NA	No association
			2	410/362	PFS	HR	F	NA	0.40(0.31-0.52)	0(0.588)	0/0/2	<0.001	0.40(0.31-0.52)	<0.001	0.40(0.31-0.52)	Y	0(0.585)	-	-	NA	Weak
			2	410/362	Objective response	RR	F	NA	3.28(2.58-4.17)	47(0.170)	0/0/2	<0.001	3.17(2.51-4.01)	<0.001	3.56(2.11-6.00)	Y	44.72(0.179)	-	-	NA	Weak
	Nivolumab or pembrolizumab vs CTx	Metastatic advanced melanoma	3	843/520	PFS	HR	F	NA	0.43(0.37-0.50)	0(0.384)	0/1/2	<0.001	0.43(0.37-0.50)	<0.001	0.43(0.37-0.50)	Y	0(0.384)	(0.16-1.14)	**0.09**	NA	Weak
			3	843/520	Objective response	RR	F	NA	3.43(2.57-4.58)	11(0.325)	0/0/3	<0.001	3.28(2.47-4.35)	<0.001	3.30(2.45-4.45)	Y	7.06(0.341)	(0.38-28.39)	**0.23**	NA	Suggestive
Wang et al. 2017	Anti-PD-1/PD-L1 vs CT	Melanoma	6	1198/974	Objective response	RR	F	<0.00001	2.80(2.37-3.30)	71(0.004)	0/0/6	<0.001	2.67(2.27-3.15)	<0.001	2.89(2.03-4.11)	Y	70.36 (< 0.001)	(0.98-8.52)	**0.51**	*p* > 0.1	Suggestive
			6	1198/974	DCR	RR	R	0.32	1.23(0.82-1.84)	96 (< 0.00001)	3/1/2	0.015	1.10(1.02-1.19)	0.270	1.23(0.85-1.76)	N	95.09 (< 0.001)	(0.33-4.59)	**0.43**	*p* > 0.1	No association
Yun et al. 2016	Anti-PD-1 or anti-CTLA-4 vs CTx or VAX	Metastatic, unresectable, cutaneous melanoma	4	1328/923	OS	RR	R	0.001	0.72(0.59-0.88)	84(0.0004)	0/0/4	<0.001	0.76(0.71-0.82)	0.001	0.72(0.60-0.88)	Y	83.34 (< 0.001)	(0.30-1.75)	**0.25**	*p* > 0.1	Suggestive
			6	1961/1235	PFS	RR	R	0.0004	0.84(0.76-0.93)	85 (< 0.00001)	0/2/4	<0.001	0.88(0.85-0.91)	<0.001	0.84(0.77-0.92)	Y	83.96 (< 0.001)	(0.61-1.16)	**0.17**	*p* > 0.1	Suggestive
			6	1961/1235	Objective response	RR	R	0.005	0.85(0.76-0.95)	89 (< 0.00001)	0/2/4	<0.001	0.89(0.86-0.92)	0.004	0.85(0.76-0.95)	N	88.49 (< 0.001)	(0.57-1.25)	**0.31**	*p* > 0.1	Suggestive
**Non-small cell lung cancer**																					
Wang et al. 2017	Anti-PD-1/PD-L1 vs CT	NSCLC	2	427/427	Objective response	RR	F	0.001	1.73(1.24-2.40)	6(0.30)	0/0/2	0.001	1.72(1.23-2.39)	0.002	1.72(1.22-2.43)	Y	5.37(0.304)	-	-	*p* > 0.1	Weak
			2	427/427	DCR	RR	R	0.75	0.95(0.69-1.31)	77(0.04)	0/1/1	0.136	0.90(0.78-1.03)	0.745	0.95(0.69-1.31)	N	77.29(0.036)	-	-	*p* > 0.1	No association
Zhou et al. 2016	Anti-PD-1/PD-L1 vs CTx	Pretreated advanced NSCLC	3	571/570	OS	HR	F	<0.001	0.71(0.61-0.81)	1.9(0.361)	0/1/2	<0.001	0.71(0.61-0.81)	<0.001	0.71(0.61-0.82)	Y	1.89(0.361)	(0.27-1.86)	**0.72**	*p* > 0.1	Suggestive
			3	571/570	PFS	HR	R	0.134	0.83(0.65-1.06)	71.3(0.031)	0/2/1	0.016	0.86(0.75-0.97)	0.134	0.83(0.65-1.06)	N	71.27(0.031)	(0.05-13.46)	**0.44**	NA	No association
			3	571/570	Overall response	OR	F	0.015	1.50(1.08-2.07)	32.1(0.229)	0/2/1	0.018	1.48(1.07-2.05)	0.059	1.49(0.99-2.24)	N	32.77(0.226)	(0.03-64.77)	**0.92**	NA	No association
Zhuansun et al. 2017	Anti-PD-1/PD-L1 vs CTx	Pretreated advanced NSCLC	4	1261/913	OS	HR	F	<0.00001	0.67(0.61-0.75)	0(0.63)	0/1/3	<0.001	0.68(0.61-0.75)	<0.001	0.68(0.61-0.75)	Y	0(0.648)	(0.53-0.86)	**0.99**	*p* > 0.1	Convincing
			4	1261/913	PFS	HR	R	0.009	0.81(0.70-0.95)	57(0.07)	0/2/2	<0.001	0.82(0.74-0.90)	0.009	0.81(0.70-0.95)	Y	56.84(0.073)	(0.45-1.49)	**0.89**	*p* > 0.1	Suggestive
			4	1261/913	Objective response	RR	F	<0.00001	0.92(0.89-0.95)	34(0.21)	0/1/3	<0.001	0.92(0.88-0.95)	<0.001	0.92(0.88-0.96)	Y	33.48(0.211)	(0.79-1.07)	**0.58**	*p* > 0.1	Suggestive
**Solid tumor**																					

Author, year	Comparison	Cancer type	RCT *N.*	Intervention/control	Outcome	Metrics	M *	Reported *p-Value* *	Reported SE (95% CI) *	Reported I2(%) (*p*-Value) *	R/N/S †	F *p-Value* §	F SE (95% CI) §	R *p-*Value §	R SE (95% CI) §	C ‡	I2(%) (*p-Value*) §	95% Prediction Interval	Egger *p-*Value	Excess Significance	Level of Evidence
Wang et al. 2017	Anti-PD-1/PD-L1 vs CT	Melanoma or NSCLC or RCC	6	1747/1467	OS	HR	R	<0.001	0.69(0.62-0.76)	0.0(0.634)	0/0/6	<0.001	0.69(0.62-0.76)	<0.001	0.69(0.62-0.76)	Y	0(0.633)	(0.59-0.80)	**0.08**	*p* > 0.1	Weak
			10	3105/2141	PFS	HR	R	<0.001	0.65(0.53-0.79)	81.2(0.000)	0/4/6	<0.001	0.70(0.65-0.76)	<0.001	0.65(0.53-0.79)	Y	81.4 (< 0.001)	(0.33-1.26)	**0.23**	*p* > 0.1	Suggestive
			9	2035/1812	Objective response	RR	F	<0.00001	2.92(2.55-3.36)	81 (< 0.00001)	0/0/9	<0.001	2.71(2.36-3.12)	<0.001	2.92(2.07-4.12)	Y	80.46 (< 0.001)	(0.93-9.19)	**0.55**	*p* > 0.1	Suggestive
			9	2035/1812	DCR	RR	R	0.25	1.15(0.91-1.45)	94(0.00001)	4/2/3	0.011	1.08(1.02-1.14)	0.233	1.15(0.92-1.43)	N	92.98 (< 0.001)	(0.51-2.58)	**0.52**	*p* > 0.1	No association
			9	2035/1812	Stable disease rate	RR	F	<0.00001	0.60(0.55-0.67)	81 (< 0.00001)	0/4/5	<0.001	0.61(0.55-0.67)	<0.001	0.58(0.45-0.75)	Y	81.01 (< 0.001)	(0.24-1.36)	**0.59**	*p* > 0.1	Suggestive
			9	2035/1812	Progressive disease rate	RR	F	<0.00001	0.80(0.74-0.87)	95(< 0.00001)	2/2/5	<0.001	0.77(0.71-0.83)	0.131	0.76(0.53-1.09)	N	94.79 (< 0.001)	(0.20-2.82)	**0.99**	*p* > 0.1	No association
**Anti-PD-1/PD-L1 solid tumor OS**	Anti-PD-1/PD-L1 vs CT	Gastric or gastro-esophageal junction cancer or head-and-neck squamous cell carcinoma or melanoma or NSCLC or RCC or urothelial	16	4681/3582	OS	HR	-	-	-	-	0/3/13	<0.001	0.73(0.69-0.78)	<0.001	0.73(0.68-0.79)	Y	38.83(0.057)	(0.59-0.92)	**0.72**	*p* > 0.1	Convincing
**Anti-PD-1/PD-L1 solid tumor PFS**	**Anti-PD-1/PD-L1 vs CT**	**Gastric or gastro-esophageal junction cancer or head-and-neck squamous cell carcinoma or melanoma or NSCLC or RCC or urothelial**	**18 (19)**	**5672/4076**	**PFS**	**HR**	-	-	-	-	**0/9/10**	**<0.001**	**0.76(0.72-0.79)**	**<0.001**	**0.73(0.65-0.84)**	**Y**	**86.79 (< 0.001)**	**(0.41-1.30)**	0.25	***p* > 0.1**	**Suggestive**

Abbreviations; RCT, randomized controlled trial; *N.*, number; SE, standard effect; CI, confidence interval; M, model; F, fixed effect; R, random effect; NA, not available; C, concordance with largest study; Y, concordant with largest study; N, not concordant with largest study; OS, overall survival; PFS progression-free survival; DCR, disease control rate; RR, risk ratio; HR, hazard ratio; OR, odds ratio; CTx, chemotherapy; CT, conventional therapy; VAX, vaccine; NSCLC, Non-small cell lung cancer; RCC, renal cell carcinoma. * Value reported in original article of the meta-analysis. † Number of individual studies of effect size with statistically significant in reverse direction/not statistically significant/statistically significant. ‡ Concordance of fixed and random effects summary outcome with outcome of largest individual study. § Value obtained from re-analysis of original meta-analysis. All *p*-Values are two-sided.

Table S3. Umbrella review summary and level of evidence reported in meta-analyses of cancer therapeutic anti-CD20 monoclonal antibodies.

Author, year	Comparison	Cancer type	RCT *N.*	Intervention/control	Outcome	Metrics	M *	Reported *p*-Value *	Reported SE (95% CI) *	Reported I2(%) (*p*-value) *	R/N/S †	F *p-Value* §	F SE (95% CI) §	R *p-Value* §	R SE (95% CI) §	C ‡	I2(%) (*p-Value*) §	95% Prediction Interval	Egger *p-Value*	Excess Significance	Level of Evidence
**Chronic Lymphocytic Leukemia**																					
Bauer et al 2012	Rituximab + CTx vs CTx	Chronic lymphocytic leukemia	3	710/711	OS	HR	F	0.033	0.78(0.62-0.98)	22(0.28)	0/2/1	0.035	0.78(0.62-0.98)	0.090	0.79(0.61-1.04)	N	20.99(0.282)	(0.08-7.51)	**0.36**	NA	No association
			3	710/711	PFS	HR	F	<0.00001	0.64(0.55-0.74)	49% (0.14)	0/1/2	<0.001	0.64(0.55-0.74)	<0.001	0.65(0.52-0.83)	Y	49.88(0.136)	(0.06-7.00)	**0.75**	*p* > 0.1	Suggestive
			3	710/711	Overall respsonse	RR	F	<0.00001	1.16(1.09-1.23)	0(0.41)	0/1/2	<0.001	1.14(1.08-1.20)	<0.001	1.14(1.08-1.20)	Y	0(0.499)	(0.81-1.61)	**0.63**	*p* > 0.1	Suggestive

Author, year	Comparison	Cancer type	RCT *N.*	Intervention/control	Outcome	Metrics	M *	Reported *p*-Value *	Reported SE (95% CI) *	Reported I2(%) (*p*-value) *	R/N/S †	F *p-Value* §	F SE (95% CI) §	R *p-Value* §	R SE (95% CI) §	C ‡	I2(%) (*p-Value*) §	95% Prediction Interval	Egger *p-Value*	Excess Significance	Level of Evidence
			3	710/711	Complete response	RR	F	<0.00001	2.11(1.72-2.59)	0(0.59)	0/1/2	<0.001	2.11(1.72-2.59)	<0.001	2.11(1.72-2.59)	Y	0(0.592)	(0.56-8.00)	**0.60**	*p* > 0.1	Suggestive
Nunes et al. 2015	Rituximab + CTx vs CTx	Chronic lymphocytic leukemia	4	1231/1202	Complete remission	OR	F	NA	2.58(2.13-3.13)	0(0.468)	0/1/3	<0.001	2.59(2.14-3.14)	<0.001	2.59(2.14-3.14)	Y	0(0.648)	(1.70-3.96)	**0.13**	*p* > 0.1	Convincing
**Non-Hodgkin's lymphoma**																					
Gao et al. 2010	Rituximab + CTx vs CTx	B-cell non-Hodgkin's lymphoma	11	2486/2447	OS	RR	F	<0.00001	1.09(1.06-1.12)	28.3(0.18)	0/6/5	<0.001	1.08(1.05-1.10)	<0.001	1.08(1.05-1.11)	Y	20.08(0.252)	(1.02-1.14)	**0.28**	*p* > 0.1	Convincing
			11	2087/2054	Overall response	RR	F	<0.00001	1.17(1.10-1.25)	72.1 (< 0.0001)	0/4/7	<0.001	1.12(1.08-1.15)	<0.001	1.17(1.10-1.24)	N	70.52(0)	(0.96-1.42)	**0.01**	*p* > 0.1	Weak
			11	2175/2125	Complete response	RR	F	<0.00001	1.52(1.27-1.82)	84.8 (< 0.00001)	0/3/8	<0.001	1.21(1.15-1.27)	<0.001	1.50(1.27-1.78)	Y	82.04(0)	(0.89-2.53)	**0.01**	*p* > 0.1	Weak
			11	2470/2333	DCR	RR	F	<0.00001	1.36(1.26-1.46)	52.1(0.02)	0/4/7	<0.001	1.33(1.27-1.39)	<0.001	1.36(1.26-1.46)	Y	51.36(0.024)	(1.11-1.67)	**0.21**	*p* > 0.1	Convincing
Hou et al. 2011	Rituximab + CTx vs CTx	B-cell non-Hodgkin's lymphoma	7	178/179	Complete response	OR	F	<0.00001	3.02(1.94-4.72)	0(0.54)	0/5/2	<0.001	2.99(1.90-4.71)	<0.001	2.99(1.90-4.71)	N	0(0.538)	(1.65-5.43)	**0.54**	*p* > 0.1	Suggestive
Ren et al. 2015	Rituximab salvage therapy vs obs	Diffuse large B-Cell lymphoma	4	202/208	OS	HR	F	0.02	0.72(0.55-0.94)	57(0.07)	0/3/1	0.015	0.72(0.55-0.94)	0.063	0.66(0.43-1.02)	N	56.53(0.075)	(0.12-3.65)	**0.28**	NA	No association
			3	183/189	PFS	HR	F	<0.00001	0.61(0.52-0.72)	54(0.11)	0/2/1	<0.001	0.61(0.52-0.72)	0.094	0.72(0.49-1.06)	N	54.46(0.111)	(0.01-42.95)	**0.15**	NA	No association
			4	202/208	Overall remission	RR	F	0.004	1.26(1.07-1.47)	56(0.08)	0/3/1	0.009	1.23(1.05-1.43)	0.185	1.19(0.92-1.53)	N	55.07(0.083)	(0.45-3.16)	**0.71**	*p* > 0.1	No association
Schulz et al. 2007	Rituximab + CTx vs CTx	Follicular lymphoma	5	759/721	OS	HR	F	<0.001	0.63(0.51-0.79)	0(0.59)	0/3/2	0.001	0.67(0.53-0.85)	0.002	0.65(0.49-0.85)	N	16.51(0.309)	(0.35-1.20)	**0.03**	*p* > 0.1	Weak
			6	808/762	Overall response	RR	F	<0.001	1.19(1.13-1.24)	79.8(< 0.001)	0/1/5	<0.001	1.13(1.08-1.17)	<0.001	1.19(1.08-1.30)	Y	72.79(0.003)	(0.89-1.59)	**0.09**	*p* > 0.1	Weak
		Indolent or mantle cell lymphoma	7	994/949	OS	HR	F	<0.001	0.65(0.54-0.78)	0(0.62)	0/4/3	<0.001	0.71(0.59-0.85)	0.001	0.70(0.57-0.87)	N	18.86(0.286)	(0.46-1.08)	**0.82**	*p* > 0.1	Suggestive
			7	979/935	Overall response	RR	F	<0.001	1.21(1.16-1.27)	81.3(< 0.001)	0/1/6	<0.001	1.14(1.10-1.19)	<0.001	1.21(1.10-1.32)	N	74.34(0.001)	(0.91-1.61)	**0.05**	*p* > 0.1	Weak
			7	979/935	Complete response	RR	F	<0.001	2.03(1.71-2.40)	84.1(< 0.001)	0/2/5	<0.001	1.74(1.48-2.06)	<0.001	2.13(1.39-3.26)	N	82.71(0)	(0.52-8.76)	**0.09**	*p* > 0.1	Weak
			7	978/935	DCR	HR	F	<0.001	0.62(0.55-0.71)	0(0.56)	0/1/6	<0.001	0.72(0.66-0.79)	<0.001	0.70(0.59-0.84)	Y	71.85(0.002)	(0.40-1.24)	**0.51**	*p* > 0.1	Suggestive
		Mantle cell lymphoma	3	130/130	OS	HR	F	0.04	0.60(0.37-0.98)	61.6(.07)	0/2/1	0.040	0.68(0.47-0.98)	0.098	0.68(0.44-1.07)	N	31.71(0.231)	(0.01-40.91)	**0.85**	*p* > 0.1	No association
			3	130/130	Overall response	RR	F	0.009	1.22(1.05-1.42)	0(0.73)	0/2/1	0.003	1.24(1.08-1.42)	0.003	1.24(1.08-1.42)	Y	0(0.739)	(0.50-3.06)	**0.74**	*p* > 0.1	Suggestive
Vidal et al. 2009	Rituximab maintenance vs obs or treatment only at relapse	Follicular lymphoma	6	985 total	OS	HR	F	0.00028	0.60(0.45-0.79)	13(.33)	0/4/2	<0.001	0.60(0.45-0.79)	<0.001	0.60(0.44-0.81)	Y	13.07(0.331)	(0.33-1.07)	**0.30**	*p* < 0.1¶	Weak
			3	454 total	PFS	HR	F	<0.00001	0.53(0.45-1.17)	55(0.11)	0/1/2	<0.001	0.53(0.42-0.66)	<0.001	0.54(0.38-0.76)	Y	56.09(0.103)	(0.01-21.69)	**0.58**	NA	Weak
			3	589 total	Event-free survival	HR	F	<0.00001	0.46(0.37-0.57)	23(0.28)	0/0/3	<0.001	0.46(0.37-0.57)	<0.001	0.46(0.35-0.59)	N	22.82(0.274)	(0.05-4.08)	**0.97**	*p* > 0.1	Suggestive
Vidal et al. 2017	Rituximab maintenance vs obs or treatment only at relapse	Follicular lymphoma	9	1145/1170	OS	HR	F	0.007	0.79(0.66-0.94)	0(0.94)	0/8/1	0.007	0.79(0.66-0.94)	0.007	0.79(0.66-0.94)	N	0(0.469)	(0.64-0.97)	**0.91**	*p* > 0.1¶	Suggestive

**Author, year**	**Comparison**	**Cancer type**	**RCT *N.***	**Interven-tion /control**	**Outcome**	**Met-rics**	**M ***	**Reported *p*-Value ***	**Reported SE (95% CI) ***	**Reported I2(%) (*p*-value) ***	**R/N/S †**	**F *p-Value* §**	**F SE (95% CI) §**	**R *p-Value* §**	**R SE (95% CI) §**	**C ‡**	**I2(%) (*p-Value*) §**	**95% Prediction Interval**	**Egger *p-Value***	**Excess Signifi-cance**	**Level of Evidence**
Zhou et al. 2017	Rituximab maintenance vs obs	Diffuse large B-Cell lymphoma	4	735/686	OS	HR	F	NA	0.90(0.70-1.16)	0(0.947)	0/4/0	0.4	0.90(0.70-1.15)	0.400	0.90(0.70-1.15)	N	0(0.946)	(0.52-1.56)	**0.32**	NA	No association
			3	658/661	PFS	HR	R	NA	0.72(0.54-0.94)	41(0.184)	0/1/2	0.001	0.71(0.58-0.88)	0.017	0.71(0.54-0.94)	Y	42.54(0.175)	(0.05-10.68)	**0.67**	*p* > 0.1	Suggestive
			**4**	**735/686**	**Event-free survival**	**HR**	**F**	**NA**	**0.80(0.65-0.98)**	**0(0.703)**	**0/3/1**	**0.004**	**0.80(0.69-0.93)**	**0.004**	**0.80(0.69-0.93)**	**N**	**0(0.735)**	**(0.58-1.11)**	**0.78**	***p* > 0.1**	**Suggestive**

Abbreviations: RCT, randomized controlled trial; *N.*, number; SE, standard effect; CI, confidence interval; M, model; F, fixed effect; R, random effect; NA, not available; C, concordance with largest study; Y, concordant with largest study; N, not concordant with largest study; OS, overall survival; PFS progression-free survival; DCR, disease control rate; RR, risk ratio; HR, hazard ratio; OR, odds ratio; CTx, chemotherapy; obs, observation. * Value reported in original article of the meta-analysis. † Number of individual studies of effect size with statistical significance in the reverse direction/not statistically significant/statistically significant. ‡ Concordance of fixed and random effects summary outcome with outcome of largest individual study. § Value obtained from re-analysis of original meta-analysis. ES is estimated by assuming power of each study could be replaced by power of the study with most cases and controls. All *p-Value*s are two-sided.

Table S4. Umbrella review summary and level of evidence reported in meta-analyses of adoptive cell immunotherapy for cancer.

Author, year	Comparison	Cancer type	RCT N.	Interven-tion/control	Outcome	Me-trics	M *	Reported p-Value *	Reported SE (95% CI) *	Reported I2(%) (p-Value) *	R/N/S †	F p-Value §	F SE (95% CI) §	R p-Value §	R SE (95% CI) §	C ‡	I2(%) (p-Value) §	95% Prediction Interval	Egger p-Value	Excess Signif-i-cance	Level of Evidence
**Dendritic cells with cytokine-induced killer cells**																					
**Hepatocellular carcinoma**																					
Su et al. 2016	DC/CIK vs obs with backbone TACE	HCC	4	127/123	0.5-year OS	OR	F	0.23	1.61(0.74–3.49)	20(0.29)	0/4/0	0.235	1.64(0.73–3.69)	0.312	1.61(0.64–4.09)	Y	32.29(0.219)	(0.10–25.45)	**0.86**	*p* > 0.1	No association
			3*	104/106	1-year OS	OR	-	-	-	-	0/3/0	0.027	2.00(1.08–3.70)	0.027	2.00(1.08–3.70)	N	0(0.733)	(0.04–107.12)	**0.12**	*p* > 0.1	Weak
			2*	72/68	2-year OS	OR	-	-	-	-	0/2/0	0.028	2.16(1.09–4.32)	0.028	2.16(1.09–4.32)	N	0(0.685)	-	-	*p* > 0.1	Weak
			5*	250/245	Overall response	OR	-	-	-	-	0/5/0	0.046	1.47(1.01–2.16)	0.046	1.47(1.01–2.16)	N	0(0.893)	(0.79–2.73)	**0.47**	*p* > 0.1	Weak
			5*	250/245	DCR	OR	-	-	-	-	0/3/2	0.013	1.81(1.13-2.90)	0.033	1.84(1.05–3.24)	Y	17.82(0.302)	(0.35–9.60)	**0.55**	*p* > 0.1	Suggestive
			4*	132/127	Quality of life	OR	-	-	-	-	0/2/2	0.001	3.07(1.58–5.97)	0.001	3.07(1.58–5.97)	N	0(0.95)	(0.71–13.23)	**0.38**	*p* > 0.1	Suggestive
**Non-small cell lung cancer**																					
Chen et al. 2014	DC/CIK vs CT or *p* or obs	NSCLC	2	75/75	1-year OS	RR	F	0.05	1.38(1.00–1.90)	35(0.21)	0/1/1	0.083	1.32(0.96–1.82)	0.207	1.29(0.87–1.93)	N	32.7(0.223)	-	-	*p* > 0.1	No association
			2	75/75	2-year OS	RR	F	0.005	2.88(1.38–5.99)	0(0.74)	0/1/1	0.005	2.88(1.38–6.01)	0.005	2.88(1.38–6.01)	Y	0(0.745)	-	-	*p* > 0.1	Weak
			2	75/75	3-year OS	RR	F	0.003	11.67 (2.28–59.69)	28(0.24)	0/1/1	0.015	7.83 (1.49–41.19)	0.023	8.14 (1.33–49.66)	Y	13.64(0.282)	-	-	*p* > 0.1	Weak
Han et al. 2014	DC/CIK + CTx vs CTx	NSCLC	5	176/177	1-year OS	RR	F	0.02	1.06(1.01–1.11)	0(0.67)	0/5/0	0.038	1.05(1.00–1.09)	0.038	1.05(1.00–1.09)	N	0(0.725)	(0.98–1.13)	**0.16**	*p* > 0.1	Weak
			4	146/147	2–year OS	RR	F	0.21	1.05(0.97–1.12)	0(0.82)	0/4/0	0.293	1.04(0.97–1.10)	0.293	1.04(0.97–1.10)	Y	0(0.845)	(0.90–1.19)	**0.40**	*p* > 0.1	No association
			5	176/177	1-year PFS	RR	F	0.005	1.09(1.03–1.15)	0(0.92)	0/5/0	0.005	1.08(1.02–1.14)	0.005	1.08(1.02–1.14)	N	0(0.926)	(0.99–1.18)	**0.33**	*p* > 0.1	Weak
			3	117/117	2-year PFS	RR	F	0.1	1.08(0.98–1.19)	0(0.84)	0/3/0	0.086	1.08(0.99–1.17)	0.086	1.08(0.99–1.17)	Y	0(0.85)	(0.63–1.84)	**0.50**	*p* > 0.1	No association
			4	159/157	Overall response	RR	F	0.76	1.06(0.74–1.51)	0(0.92)	0/4/0	0.829	1.04(0.73–1.47)	0.829	1.04(0.73–1.47)	Y	0(0.921)	(0.48–2.24)	**0.08**	*p* > 0.1	No association
			4	159/157	Partial response	RR	F	0.22	1.23(0.88–1.71)	0(0.78)	0/4/0	0.208	1.23(0.89–1.71)	0.208	1.23(0.89–1.71)	Y	0(0.782)	(0.60–2.53)	**0.74**	*p* > 0.1	No association
			3	120/121	DCR	RR	F	0.006	1.28(1.07–1.52)	3(0.36)	0/2/1	0.009	1.25(1.06–1.47)	0.009	1.25(1.06–1.47)	N	0(0.37)	(0.43–3.62)	**0.26**	*p* > 0.1	Suggestive
Wang et al. 2015	DC/CIK + CT vs CT	NSCLC	5	222/223	OS	HR	F	<0.001	0.62(0.49–0.79)	0.0(0.795)	0/3/2	<0.001	0.62(0.49–0.79)	<0.001	0.62(0.49–0.79)	Y	0(0.796)	(0.42–0.91)	**0.99**	*p* > 0.1	Suggestive
			5	191/192	PFS	HR	F	<0.001	0.53(0.39–0.71)	0.0(0.700)	0/2/3	<0.001	0.53(0.39–0.71)	<0.001	0.53(0.39–0.71)	Y	0(0.704)	(0.32–0.86)	**0.76**	*p* > 0.1	Suggestive
			2	91/91	Objective response	RR	F	0.65	1.19(0.56–2.53)	0.0(0.544)	0/2/0	0.692	1.17(0.54–2.50)	0.692	1.17(0.54–2.50)	Y	0(0.545)	-	-	NA	No association

Author, year	Comparison	Cancer type	RCT *N.*	Intervention/control	Outcome	Metrics	M *	Reported *p*-Value *	Reported SE (95% CI) *	Reported I2(%) (*p*-Value) *	R/N/S †	F *p*-Value §	F SE (95% CI) §	R *p*-Value §	R SE (95% CI) §	C ‡	I2(%) (*p*-Value) §	95% Prediction Interval	Egger *p*-Value	Excess Significance	Level of Evidence
			3	133/133	DCR	RR	F	0.009	1.25(1.06–1.48)	0.0(0.424)	0/2/1	0.018	1.21(1.03–1.42)	0.018	1.21(1.03–1.42)	Y	0(0.468)	(0.43–3.41)	**0.06**	NA	Weak
Zheng et al. 2015	DC/CIK + CTx vs CTx	NSCLC	2	56/56	0.5-year OS	RR	F	0.01	1.16(1.04-1.31)	0(0.98)	0/1/1	0.010	1.16(1.04–1.31)	0.010	1.16(1.04–1.31)	Y	0(0.979)	–	**-**	NA	Weak
			4	204/203	1-year OS	OR	F	0.04	1.57(1.02–2.40)	0(0.71)	0/3/1	0.040	1.56(1.02–2.40)	0.040	1.56(1.02–2.40)	N	1.97(0.382)	(0.61–4.00)	**0.67**	*p* > 0.1	Suggestive
			4	196/195	2–year OS	RR	F	0.002	1.30(1.10–1.53)	0(0.46)	0/3/1	0.002	1.24(1.08–1.42)	0.002	1.24(1.08–1.42)	N	0(0.551)	(0.92–1.67)	**0.03**	*p* > 0.1	Weak
			3	135/134	3-year OS	RR	F	0.0007	1.51(1.19–1.91)	0(0.80)	0/1/2	0.001	1.49(1.18–1.88)	<0.001	1.49(1.18–1.88)	Y	0(0.806)	(0.33–6.73)	**0.13**	*p* > 0.1	Suggestive
			3	141/141	Overall response	RR	F	0.45	1.15(0.80–1.65)	0(0.83)	0/3/0	0.475	1.14(0.80–1.62)	0.475	1.14(0.80–1.62)	Y	0(0.831)	(0.11–11.34)	**0.37**	*p* > 0.1	No association
			3	141/141	DCR	RR	F	0.002	1.31(1.11–1.55)	17(0.30)	0/1/2	0.005	1.26(1.07–1.48)	0.007	1.26(1.07–1.50)	Y	10.08(0.329)	(0.36–4.47)	**0.22**	*p* > 0.1	Suggestive
**DC/CIK NSCLC OS**	DC/CIK + CT vs CT	NSCLC	10	387/427	OS(longest)	RR	-	-	-	-	0/7/3	<0.001	0.82(0.75–0.89)	<0.001	0.82(0.75–0.89)	Y	0(0.589)	(0.74–0.90)	**0.19**	*p* > 0.1	Suggestive
**Solid tumor**																					
Lan et al. 2015	DC/CIK + CTx vs CTx	NSCLC or rectal cancer or colorectal cancer or colon cancer or breast cancer or gastric cancer	2	69/69	1-year DFS	OR	F	0.01	0.16(0.04-0.67)	0(0.53)	0/2/0	0.020	0.18(0.04–0.76)	0.020	0.18(0.04–0.76)	N	0(0.543)	-	-	*p* > 0.1	Weak
			2	69/69	2-year DFS	OR	F	0.06	0.49(0.23–1.04)	65(0.09)	0/1/1	0.072	0.49(0.23–1.07)	0.249	0.46(0.12–1.72)	Y	64.52(0.093)	-	-	*p* > 0.1	No association
			2	69/69	3-year DFS	OR	F	0.003	0.32(0.16–0.68)	42(0.19)	0/1/1	0.003	0.32(0.15–0.68)	0.021	0.31(0.12–0.84)	N	42.12(0.189)	–	–	*p* > 0.1	Weak
			6	177/175	1-year OS	OR	F	<0.0001	0.22(0.11–0.44)	5(0.39)	0/5/1	<0.001	0.22(0.11–0.45)	<0.001	0.23(0.11-0.48)	N	4.54(0.388)	(0.07-0.75)	**0.69**	*p* > 0.1	Suggestive
			6	177/175	2-year OS	OR	F	<0.0001	0.28(0.14-0.53)	0(0.92)	0/5/1	<0.001	0.28(0.15-0.53)	<0.001	0.28(0.15-0.53)	N	0(0.921)	(0.11-0.70)	**0.92**	*p* > 0.1	Suggestive
			5	195/202	3-year OS	OR	F	0.009	0.41(0.25-0.70)	35(0.19)	0/4/1	0.002	0.42(0.25-0.72)	0.007	0.37(0.18-0.77)	N	34.53(0.191)	(0.06-2.51)	**0.28**	*p* > 0.1	Suggestive
			6	207/224	Overall response	OR	F	0.004	0.54(0.35-0.82)	0(0.63)	0/5/1	0.005	0.54(0.35-0.83)	0.005	0.54(0.35-0.83)	Y	0(0.634)	(0.30-0.99)	**0.36**	*p* > 0.1	Suggestive
			5	175/192	DCR	OR	F	0.001	0.46(0.28-0.74)	0(0.99)	0/4/1	0.001	0.46(0.28-0.74)	0.001	0.46(0.28-0.74)	Y	0(0.994)	(0.21-1.00)	**0.51**	*p* > 0.1	Suggestive
**Cytokine-induced killer cells**																					
**Hepatocellular carcinoma**																					
Li et al. 2016	Adjuvant CIK vs no AT	Treated HCC, Barcelona clinic liver cancer B or earlier stage	5	380/335	OS	RR	R	0.0003	0.76(0.65-0.88)	50(0.009)	0/3/2	0.005	0.82(0.71-0.94)	0.021	0.78(0.64-0.96)	Y	41.96(0.142)	(0.44-1.39)	**0.04**	*p* > 0.1	Weak
			7	460/405	PFS	RR	R	0.007	0.74(0.59-0.92)	75(0.0006)	0/5/2	<0.001	0.85(0.78-0.92)	0.004	0.76(0.63-0.91)	N	65.76(0.008)	(0.45-1.29)	**0.02**	*p* > 0.1	Weak
Wang et al. 2016	CIK vs no AT after resection	Resected HCC	4	374/329	1-year OS	RR	F	0.001	1.08(1.03-1.13)	0(0.42)	0/2/2	0.001	1.07(1.03-1.12)	<0.001	1.07(1.03-1.12)	Y	0(0.43)	(0.98-1.17)	**0.98**	*p* > 0.1	Suggestive
			3	290/286	2-year OS	RR	F	0.0002	1.14(1.06-1.23)	0(0.57)	0/2/1	<0.001	1.14(1.06-1.22)	<0.001	1.14(1.06-1.22)	Y	0(0.578)	(0.73-1.78)	**0.95**	*p* > 0.1	Suggestive

Author, year	Comparison	Cancer type	RCT *N.*	Interven-tion /control	Outcome	Me t-rics	M *	Reported *p*-Value *	Reported SE (95% CI) *	Reported I2(%) (*p*-Value) *	R/N/S †	F *p*-Value §	F SE (95% CI) §	R *p*-Value §	R SE (95% CI) §	C ‡	I2(%) (*p*-Value) §	95% Prediction Interval	Egger *p*-Value	Excess Significance	Level of Evidence
			5	402/357	3-year OS	RR	F	0.02	1.15(1.03-1.28)	0(0.81)	0/5/0	0.010	1.15(1.03-1.28)	0.010	1.15(1.03-1.28)	N	0(0.811)	(0.97-1.36)	**0.91**	*p* > 0.1	Weak
			3	290/286	4-year OS	RR	F	0.55	1.07(0.86-1.32)	0(0.90)	0/3/0	0.469	1.08(0.88-1.33)	0.469	1.08(0.88-1.33)	Y	0(0.903)	(0.28-4.14)	**0.14**	*p* > 0.1	No association
			3	260/217	5-year OS	RR	F	0.9	0.98(0.73-1.31)	0(0.92)	0/3/0	0.936	0.99(0.74-1.32)	0.936	0.99(0.74-1.32)	Y	0(0.922)	(0.15-6.39)	**0.20**	*p* > 0.1	No association
			5	419/369	1-year recurrence-free survival	RR	R	0.001	1.22(1.08-1.37)	50(0.09)	0/1/4	<0.001	1.20(1.11-1.31)	<0.001	1.22(1.09-1.36)	Y	48.01(0.103)	(0.86-1.71)	**0.15**	*p* > 0.1	Suggestive
			3	290/286	2-year recurrence-free survival	RR	F	<0.0001	1.37(1.18-1.59)	34(0.22)	0/1/2	<0.001	1.36(1.17-1.59)	0.001	1.36(1.13-1.64)	Y	34.32(0.218)	(0.24-7.66)	**0.98**	*p* > 0.1	Suggestive
			5	402/357	3-year recurrence-free survival	RR	F	0.004	1.35(1.10-1.65)	7(0.37)	0/4/1	0.005	1.33(1.09-1.63)	0.007	1.33(1.08-1.64)	Y	6.8(0.368)	(0.90-1.98)	**0.87**	*p* > 0.1	Suggestive
			3	290/286	4-year recurrence-free survival	RR	F	0.66	1.05(0.74-1.48)	0(0.66)	0/3/0	0.742	1.06(0.75-1.50)	0.742	1.06(0.75-1.50)	Y	0(0.66)	(0.11-10.04)	**0.41**	*p* > 0.1	No association
			3	260/217	5-year recurrence-free survival	RR	F	0.54	1.17(0.71-1.94)	18(0.29)	0/3/0	0.540	1.17(0.70-1.97)	0.622	1.16(0.64-2.09)	Y	18.33(0.294)	(0.01-142.53)	**0.64**	*p* > 0.1	No association
Yu et al. 2017	Adjuvant CIK + CT vs CT	HCC	7	451/422	OS	HR	R	0.000	0.64(0.51-0.82)	50.4(0.060)	0/3/4	<0.001	0.66(0.57-0.77)	<0.001	0.64(0.51-0.82)	Y	50.33(0.06)	(0.34-1.23)	**0.25**	*p* > 0.1	Suggestive
			4	263/216	Recurrence-free survival	HR	R	0.000	0.64(0.51-0.78)	0(0.781)	0/1/3	<0.001	0.63(0.51-0.78)	<0.001	0.63(0.51-0.78)	Y	0(0.781)	(0.40-1.01)	**0.23**	*p* > 0.1	Suggestive
**Other adoptive cellular immunotherapies**																					
**Hepatocellular carcinoma**																					
Xie et al. 2012	Postoperative ACI (LAK or CIK) vs no AT after curative resection	Pretreated HCC	2	96/55	3-year OS	OR	F	0.792	0.91(0.45-1.84)	0.0(0.708)	0/2/0	0.792	0.91(0.45-1.84)	0.792	0.91(0.45-1.84)	Y	0(0.724)	-	-	*p* > 0.1	No association
			3	85/78	1-year recurrence rate	OR	F	0.003	0.35(0.17-0.71)	6.7(0.342)	0/3/0	0.011	0.39(0.19-0.81)	0.013	0.39(0.18-0.82)	N	6.28(0.344)	(0.00-63.67)	**0.27**	*p* > 0.1	Weak
			3	85/78	3-year recurrence rate	OR	F	0.001	0.31(0.16-0.61)	0.0(0.648)	0/1/2	0.001	0.32(0.16-0.62)	0.001	0.32(0.16-0.62)	Y	0(0.914)	(0.00-25.05)	**0.52**	*p* > 0.1	Suggestive
Yuan et al. 2017	Postoperative ACI (CIK or LAK + IL-2 or lymphocytes) vs no AT	Pretreated HCC, not advanced	6¶	407/362	1-year mortality	RR	-	-	-	-	0/5/1	0.035	0.50(0.26-0.95)	0.032	0.43(0.20-0.93)	N	17.93(0.297)	(0.09-2.12)	**0.01**	*p* > 0.1	Weak
			6¶	407/362	2-year mortality	RR	-	-	-	-	0/6/0	0.002	0.52(0.34-0.78)	0.002	0.52(0.34-0.78)	N	0(0.685)	(0.29-0.93)	**0.59**	*p* > 0.1	Weak
			6¶	407/362	3-year mortality	RR	-	-	-	-	0/5/1	0.009	0.71(0.55-0.92)	0.009	0.71(0.55-0.92)	N	0(0.593)	(0.49-1.02)	**0.81**	*p* > 0.1	Suggestive
			2¶	160/117	5-year mortality	RR	-	-	-	-	0/2/0	0.920	0.99(0.83-1.19)	0.920	0.99(0.83-1.19)	Y	0(0.966)	-	-	*p* > 0.1	No association

Author, year	Comparison	Cancer type	RCT N.	Interven-tion /control	Outcome	Me-trics	M *	Reported p-Value *	Reported SE (95% CI) *	Reported I2(%) (p-Value) *	R/N/S †	F p-Value §	F SE (95% CI) §	R p-Value §	R SE (95% CI) §	C ‡	I2(%) (p-Value) §	95% Prediction Interval	Egger p-Value	Excess Significance	Level of Evidence
			8¶	483/432	1-year recurrence rate	RR	-	-	-	-	0/6/2	<0.001	0.54(0.42-0.71)	<0.001	0.54(0.42-0.71)	N	0(0.495)	(0.39-0.75)	**0.41**	*p* > 0.1	Suggestive
			6¶	407/362	2-year recurrence rate	RR	-	-	-	-	0/3/3	<0.001	0.62(0.51-0.75)	<0.001	0.62(0.51-0.75)	Y	0(0.426)	(0.48-0.81)	**0.80**	*p* > 0.1	Suggestive
			6¶	407/362	3-year recurrence rate	RR	-	-	-	-	0/5/1	0.001	0.81(0.72-0.92)	0.001	0.81(0.72-0.92)	N	0(0.772)	(0.68-0.97)	**0.30**	*p* > 0.1	Suggestive
			2¶	160/117	5-year recurrence rate	RR	-	-	-	-	0/2/0	0.110	0.92(0.83-1.02)	0.110	0.92(0.83-1.02)	Y	0(0.58)	-	-	*p* > 0.1	No association
**Non-small cell lung cancer**																					-
Mi et al. 2016	LAK + IL-2 + CTx vs CTx	NSCLC	2	82/88	OS	HR	R	0.04	0.54(0.30-0.97)	55(0.14)	0/1/1	0.003	0.56(0.38-0.82)	0.039	0.54(0.30-0.97)	N	54.81(0.137)	-	-	NA	Weak
	DC/CIK or CIK + CT vs CT	NSCLC	2	43/44	OS	HR	F	0.01	0.55(0.35-0.87)	0(0.84)	0/1/1	0.010	0.56(0.35-0.87)	0.010	0.56(0.35-0.87)	Y	0(0.856)	-	-	NA	Weak
	DC/CIK or CIK + CTx vs CTx	NSCLC	3	98/98	DCR	OR	F	0.006	2.84(1.35-5.97)	0(0.46)	0/2/1	0.040	2.79(1.31-5.94)	0.067	2.79(1.31-5.94)	N	0(0.419)	(0.02-377.46)	**0.31**	*p* > 0.1	No association
	Postoperative ACI (LAK + IL-2 or TIL + IL-2) + CT vs CT	NSCLC	3	138/145	OS	HR	F	0.0003	0.60(0.46-0.79)	20(0.29)	0/1/2	<0.001	0.60(0.46-0.79)	0.001	0.60(0.44-0.81)	Y	19.95(0.287)	(0.04-7.93)	**0.40**	*p* > 0.1	Suggestive
	ACI(CIK or DC/CIK or TIL) + CT vs CT	NSCLC	4	255/210	DCR	OR	F	0.004	2.02(1.24-3.29)	0(0.40)	0/3/1	0.007	1.98(1.21-3.25)	0.007	1.98(1.21-3.25)	N	3.77(0.374)	(0.67-5.86)	**0.25**	*p* > 0.1	Suggestive
	IT(CIK or DC/CIK or IL-2) + CTx vs CTx	NSCLC	3	170/156	OS	HR	F	0.04	0.77(0.60-0.99)	34(0.22)	0/2/1	0.040	0.77(0.60-0.99)	0.067	0.72(0.51-1.02)	N	32.2(0.229)	(0.03-18.03)	**0.41**	NA	No association
Zeng et al. 2016	Postoperative ACI(AKT-DC or DC/CIK or LAK + IL-2 or TIL + rIL-2) + CT vs CT	Resected NSCLC	4	234/238	OS	HR	F	0.002	0.61(0.45-0.84)	42(0.16)	0/3/1	0.002	0.62(0.45-0.84)	0.013	0.59(0.39-0.89)	N	39.65(0.174)	(0.14-2.56)	**0.16**	*p* > 0.1	Suggestive
Zhao et al. 2017	ACI(LAK + IL-2 or DC/CIK or CIK or AKT or TIL) vs CT	NSCLC, operated or non-operated	13¶	718/844	1-year OS	RR	-	-	-	-	0/8/5	<0.001	1.15(1.10-1.21)	<0.001	1.19(1.11-1.27)	Y	25.14(0.19)	(1.03-1.37)	**0.01**	*p* > 0.1	Weak
			11¶	669/755	2-year OS	RR	-	-	-	-	0/5/6	<0.001	1.43(1.30-1.58)	<0.001	1.43(1.30-1.58)	Y	0(0.562)	(1.28-1.61)	**0.24**	*p* > 0.1	Convincing
			8¶	529/613	3-year OS	RR	-	-	-	-	0/5/3	<0.001	1.45(1.24-1.69)	<0.001	1.45(1.24-1.69)	N	0(0.639)	(1.19-1.76)	**0.31**	*p* > 0.1	Suggestive
			5¶	400/485	5-year OS	RR	-	-	-	-	0/2/3	<0.001	1.71(1.39-2.11)	0.031	1.67(1.05-2.67)	N	77.03(0.002)	(0.32-8.72)	**0.87**	*p* > 0.1	Suggestive
			4¶	187/229	1-year PFS	RR	-	-	-	-	0/2/2	<0.001	1.46(1.24-1.72)	0.031	1.46(1.24-1.72)	Y	0(0.877)	(1.02-2.09)	**0.16**	*p* > 0.1	Suggestive
			2¶	137/138	2-year PFS	RR	-	-	-	-	0/1/1	<0.001	1.69(1.29-2.23)	0.027	1.79(1.07-2.99)	N	68.9(0.073)	-	-	*p* > 0.1	Weak
			4	141/182	Objective response	RR	F	0.293	1.21(0.85-1.72)	0.0(3.98)	0/3/1	0.220	1.25(0.88-1.77)	0.220	1.25(0.88-1.77)	Y	0(0.402)	(0.58-2.70)	**0.59**	*p* > 0.1	No association

Author, year	Comparison	Cancer type	RCT *N.*	Intervention/control	Outcome	Metrics	M *	Reported *p*-Value *	Reported SE (95% CI) *	Reported I2(%) (*p*-Value) *	R/N/S †	F *p*-Value §	F SE (95% CI) §	R *p*-Value §	R SE (95% CI) §	C ‡	I2(%) (*p*-Value) §	95% Prediction Interval	Egger *p*-Value	Excess Significance	Level of Evidence
			4	141/182	DCR	RR	R	0.123	1.16(0.96-1.40)	52.4(0.098)	0/2/2	0.055	1.13(1.00-1.27)	0.099	1.16(0.97-1.37)	N	43.21(0.152)	(0.62-2.16)	**0.40**	*p* > 0.1	No association
**Renal cell carcinoma**																					
Tang et al. 2013	ACI(autolymphocyte or LAK or TIL or CIK) vs no ACI	Metastatic RCC	4	235/224	1-year OS	RR	F	0.0008	1.30(1.12-1.52)	0(0.58)	0/3/1	<0.001	1.33(1.15-1.54)	<0.001	1.33(1.15-1.54)	N	0(0.603)	(0.97-1.83)	**0.205**	*p* > 0.1	Suggestive
			3	154/155	3-year OS	RR	F	<0.00001	2.76(1.85-4.14)	46(0.16)	0/1/2	<0.001	2.61(1.74-3.92)	0.005	2.63(1.33-5.19)	Y	45.26(0.161)	(0.00-2468.29)	**0.89**	*p* > 0.1	Suggestive
			2	109/110	5-year OS	RR	F	0.01	2.42(1.21-4.83)	28(0.24)	0/1/1	0.017	2.36(1.16-4.77)	0.062	2.26(0.96-5.31)	N	27.88(0.239)	-	-	*p* > 0.1	No association
			**4**	**226/228**	**Objective response**	**RR**	**F**	**0.007**	**1.65(1.15-2.38)**	**49(0.12)**	**0/3/1**	**0.005**	**1.70(1.17-2.46)**	**0.270**	**1.50(0.73-3.08)**	**N**	**49.11(0.117)**	**(0.10-21.44)**	**0.60**	***p* > 0.1**	**No association**

Abbreviations: RCT, randomized controlled trial; *N.*, number; SE, standard effect; CI, confidence interval; M, model; F, fixed effect; R, random effect; NA, not available; C, concordance with largest study; Y, concordant with largest study; N, not concordant with largest study; OS, overall survival; PFS progression-free survival; RR, risk ratio; HR, hazard ratio; OR, odds ratio; DC/CIK, dendritic cells with cytokine-induced killer cells; obs, observation; CT, conventional therapy; *p*, placebo; CTx, chemotherapy; CIK, cytokine-induced killer cells; AT, adjuvant therapy; ACI, adjuvant cell immunotherapy; LAK, lymphokine-activated killer cells; IL-2, Interleukin-2; TIL, tumor-infiltrating lymphocytes; AKT, activated killer T-cells; HCC, hepatocellular carcinoma; NSCLC, non-small cell lung cancer; RCC, renal cell cancer. * Value reported in original article of the meta-analysis. † Number of individual studies of effect size with statistical significance in the reverse direction/not statistically significant/statistically significant. ‡ Concordance of fixed and random effects summary outcome with outcome of largest individual study. § Value obtained from re-analysis of original meta-analysis. Re-analysis was performed after excluding non-RCTs from original meta-analysis. All *p-Value*s are two-sided.

Table S5. Umbrella review summary and level of evidence reported in meta-analyses of cancer therapeutic immunomodulatory cytokines.

Author, year	Comparison	Cancer type	RCT *N.*	Intervention/control	Outcome	Metrics	M *	Reported *p-Value* *	Reported SE (95% CI) *	Reported I2(%) (*p-Value*) *	R/N/S †	F *p-Value* §	F SE (95% CI) §	R *p-Value* §	R SE (95% CI) §	C ‡	I2(%) (*p-Value*) §	95% Prediction Interval	Egger *p-Value*	Excess Significance	Level of Evidence
**Interferon- α**																					
**Colorectal cancer**																					
Thirion et al. 2000	IFN-α + 5FU vs leucovorin + 5FU	Colorectal cancer	7	744/744	OS	HR	NA	0.066	1.11(0.99-1.24)	NA (0.495)	0/7/0	0.036	1.02(0.98-1.05)	0.403	1.02(0.98-1.05)	Y	0(0.494)	(0.96-1.08)	**0.38**	*p* > 0.1	No association
			7	650/655	Response rate	RR	NA	0.042	1.26(1.01-1.59)	NA (0.001)	2/5/0	0.036	1.06(1.00-1.12)	0.276	1.08(0.94-1.24)	Y	79.46(0)	(0.69-1.68)	**0.77**	*p* > 0.1	No association
	IFN-α + 5FU vs 5FU, with or without leucovorin in both arms	Colorectal cancer	12	879/887	OS	HR	NA	0.33	0.95(0.86-1.05)	NA (0.11)	0/12/0	0.203	1.02(0.99-1.05)	0.203	1.02(0.99-1.05)	Y	0(0.866)	(0.99-1.06)	**0.97**	*p* > 0.1	No association
			12	838/845	Response rate	RR	NA	0.8	1.02(0.87-1.20)	NA (0.15)	0/11/1	0.997	1.00(0.95-1.05)	0.893	1.00(0.94-1.07)	Y	34.15(0.117)	(0.85-1.18)	**0.78**	*p* > 0.1	No association
**Hepatocellular carcinoma**																					
Jiang et al. 2013		Pretreated HCC, viral hepatitis related	9	498/451	Mortality	OR	F	<0.00001	0.42(0.32-0.56)	0(0.54)	0/6/3	<0.001	0.43(0.32-0.56)	<0.001	0.43(0.32-0.56)	Y	0(0.536)	(0.31-0.59)	**0.91**	*p* > 0.1	Suggestive

**Author, year**	**Comparison**	**Cancer type**	**RCT *N.***	**Interven-tion /control**	**Outcome**	**Met-rics**	**M ***	**Reported *p-Value* ***	**Reported SE (95% CI) ***	**Reported I2(%) (*p-Value*) ***	**R/N/S †**	**F *p-Value* §**	**F SE (95% CI) §**	**R *p-Value* §**	**R SE (95% CI) §**	**C ‡**	**I2(%) (*p-Value*) §**	**95% Prediction Interval**	**Egger *p-Value***	**Excess Signifi-cance**	**Level of Evidence**
	Post-surgical adjuvant IFN(mostly IFN-α) vs *p* after surgical resection or TACE		9	499/476	Recurrence rate	OR	F	0.002	0.66(0.50-0.86)	0(0.67)	0/8/1	0.003	0.66(0.51-0.87)	0.003	0.66(0.51-0.87)	N	0(0.667)	(0.48-0.92)	**0.08**	*p* > 0.1	Weak
**Melanoma**																					
Ives et al. 2007	IFN-α + CTx vs CTx	Metastatic melanoma	8	678/585	OS	OR	NA	0.9	0.99(0.88-1.12)	NA (0.002)	0/6/2	0.991	1.00(0.72-1.40)	0.505	1.23(0.67-2.26)	Y	62.46(0.009)	(0.21-7.16)	**0.06**	*p* > 0.1	No association
			11	683/607	Overall response	OR	NA	0.0002	0.60(0.46-0.79)	NA (0.4)	0/8/3	0.001	0.58(0.44-0.77)	<0.001	0.58(0.44-0.77)	Y	0(0.459)	(0.40-0.84)	**0.49**	*p* > 0.1	Convincing
			10	662/583	Complete response	OR	NA	<0.00001	0.33(0.20-0.53)	NA (0.7)	0/8/2	<0.001	0.33(0.19-0.57)	<0.001	0.33(0.19-0.57)	Y	0(0.796)	(0.17-0.64)	**0.54**	*p* > 0.1	Convincing
			10	662/583	Partial response	OR	NA	0.2	0.81(0.59-1.11)	NA (0.9)	0/10/0	0.223	0.82(0.59-1.13)	0.223	0.82(0.59-1.13)	Y	0(0.9)	(0.56-1.20)	**0.54**	*p* > 0.1	No association
Ives et al. 2017	Adjuvant IFN-α vs obs	High-risk malignant melanoma	18	4520/3179	OS	HR	NA	0.003	NA	NA (0.8)	0/17/1	0.017	0.91(0.84-0.98)	0.017	0.91(0.84-0.98)	N	0(0.992)	(0.83-0.99)	**0.22**	*p* > 0.1	Suggestive
			18	4520/3177	Event-free survival	HR	NA	<0.00001	NA	NA (0.8)	0/18/0	<0.001	0.86(0.80-0.92)	<0.001	0.86(0.80-0.92)	N	0(0.981)	(0.79-0.93)	**0.23**	*p* > 0.1	Weak
Mocellin et al. 2013	Adjuvant IFN-α vs CT	Cutaneous melanoma	15	5412/3771	OS	HR	F	0.0029	0.91(0.85-0.97)	6(0.38)	1/10/4	0.003	0.91(0.85-0.97)	0.004	0.91(0.85-0.97)	N	4.77(0.399)	(0.82-1.00)	**0.06**	*p* > 0.1¶	Weak
			17	5638/3963	Disease-free survival	HR	F	<0.00001	0.83(0.78-0.87)	16(0.27)	0/10/7	<0.001	0.83(0.78-0.87)	<0.001	0.82(0.77-0.88)	N	12.8(0.304)	(0.73-0.93)	**0.03**	*p* > 0.1¶	Weak
Myeloma Trialists' Collaborative Group. 2011	IFN in induction therapy vs CT	Melanoma	12	1230/1239	Mortality	OR	NA	0.1	NA	NA (0.2)	0/12/0	0.199	0.89(0.74-1.06)	0.199	0.89(0.74-1.06)	Y	0(0.878)	(0.72-1.09)	**0.14**	*p* > 0.1	No association
			10	685/638	PFS	OR	NA	0.0003	NA	NA (0.07)	0/9/1	0.089	0.81(0.63-1.03)	0.138	0.80(0.59-1.08)	Y	23.61(0.226)	(0.42-1.51)	**0.33**	*p* > 0.1	No association
	IFN in maintenance therapy vs CT	Melanoma	12	767/776	Mortality	OR	NA	0.04	NA	NA (0.1)	0/11/1	0.072	0.81(0.65-1.02)	0.083	0.81(0.64-1.03)	Y	9.77(0.349)	(0.54-1.21)	**0.48**	*p* > 0.1	No association
			12	767/777	PFS	OR	NA	<0.00001	NA	NA (0.03)	0/7/5	<0.001	0.57(0.44-0.73)	<0.001	0.53(0.38-0.76)	N	40.34(0.072)	(0.21-1.34)	**0.06**	*p* > 0.1	Weak
Pirard et al. 2004	Postsurgical adjuvant IFN-α vs control	Postsurgical Melanoma, stage IV unincluded	9	1399/1438	OS	OR	F	0.1029	0.87(0.74-1.02)	NA (0.7376)	0/9/0	0.107	0.88(0.74-1.03)	0.107	0.88(0.74-1.03)	Y	0(0.733)	(0.72-1.06)	**0.33**	*p* > 0.1	No association
			10	1483/1508	Recurrence rate	OR	F	0.0001	0.74(0.64-0.86)	NA (0.2808)	0/7/3	<0.001	0.75(0.64-0.87)	<0.001	0.74(0.62-0.88)	N	17.29(0.284)	(0.53-1.03)	**0.68**	*p* > 0.1	Suggestive
Sasse et al. 2013	IFN-α + CTx vs CTx	Metastatic malignant melanoma	4	266/260	OS	OR	F	0.15	0.74(0.49-1.12)	0(0.87)	0/4/0	0.159	0.74(0.49-1.13)	0.159	0.74(0.49-1.13)	Y	0(0.872)	(0.29-1.86)	**0.17**	*p* > 0.1	No association
			7	548/460	1-year survival	RR	R	0.18	1.18(0.93-1.50)	30(0.20)	0/6/1	0.116	1.16(0.96-1.40)	0.176	1.18(0.93-1.50)	Y	29.98(0.199)	(0.68-2.03)	**0.58**	*p* > 0.1	No association
			6	518/429	2-year survival	RR	R	0.33	1.19(0.84-1.67)	0(0.63)	0/6/0	0.328	1.19(0.84-1.67)	0.328	1.19(0.84-1.67)	Y	0(0.629)	(0.73-1.92)	**0.25**	*p* > 0.1	No association
			10	705/626	Overall response	RR	R	0.036	1.32(1.02-1.71)	23(0.23)	0/8/2	0.010	1.33(1.07-1.66)	0.036	1.32(1.02-1.71)	Y	23.12(0.23)	(0.76-2.29)	**0.59**	*p* > 0.1	Suggestive
Verma et al. 2006	Adjuvant IFN (high dose, mostly IFN-α) vs VAX or obs	Resected melanoma	3	802/792	2-year mortality	RR	R	0.03	0.85(0.73-0.99)	NA (0.91)	0/3/0	0.031	0.85(0.73-0.99)	0.031	0.85(0.73-0.99)	N	0(0.914)	(0.31-2.28)	**0.09**	*p* > 0.1	Weak
Wheatley et al. 2003	IFN-α vs obs	Metastatic, high-risk melanoma	13	3075/2007	OS	HR	NA	0.1	NA	NA (0.9)	0/13/0	0.462	0.98(0.92-1.04)	0.462	0.98(0.92-1.04)	Y	0.00(0.964)	(0.91-1.05)	**0.08**	NA	No association
			14	3144/2037	Recurrence-free survival	HR	NA	<0.00001	NA	NA (0.4)	0/11/3	<0.001	0.92(0.88-0.97)	<0.001	0.92(0.88-0.97)	Y	4.86(0.398)	(0.86-0.99)	**0.07**	NA	Weak

Author, year	Comparison	Cancer type	RCT N.	Intervention/control	Outcome	Metrics	M *	Reported p-Value *	Reported SE (95% CI) *	Reported I2(%) (p-Value) *	R/N/S †	F p-Value §	F SE (95% CI) §	R p-Value §	R SE (95% CI) §	C ‡	I2(%) (p-Value) §	95% Prediction Interval	Egger p-Value	Excess Significance	Level of Evidence
Xin et al. 2016	IFN-α + dacarbazine vs dacarbazine	Cutaneous malignant melanoma	7	545/448	1-year OS	RR	F	0.28	1.10(0.92-1.31)	42(0.11)	0/6/1	0.272	1.10(0.93-1.32)	0.316	1.14(0.88-1.46)	Y	41.76(0.112)	(0.60-2.15)	**0.39**	$p > 0.1$	No association
Xin et al. 2016	IFN-α + dacarbazine vs dacarbazine	Cutaneous malignant melanoma	5	327/233	2-year OS	RR	R	0.05	1.59(0.99-2.54)	0(0.64)	0/5/0	0.700	1.55(0.96-2.50)	0.700	1.55(0.96-2.50)	Y	0(0.639)	(0.72-3.37)	**0.17**	$p > 0.1$	No association
Xin et al. 2016	IFN-α + dacarbazine vs dacarbazine	Cutaneous malignant melanoma	2	228/142	3-year OS	RR	F	0.4	0.62(0.20-1.87)	0(0.71)	0/2/0	0.393	0.62(0.20-1.87)	0.393	0.62(0.20-1.87)	Y	0(0.713)	-	-	$p > 0.1$	No association
Xin et al. 2016	IFN-α + dacarbazine vs dacarbazine	Cutaneous malignant melanoma	8	438/357	Overall response	RR	R	0.0008	1.59(1.21-2.08)	0(0.72)	0/6/2	0.001	1.59(1.21-2.09)	<0.001	1.59(1.21-2.09)	N	0(0.717)	(1.13-2.24)	**0.40**	$p > 0.1$	Suggestive
Xin et al. 2016	IFN-α + dacarbazine vs dacarbazine	Cutaneous malignant melanoma	8	438/357	Complete response	RR	R	<0.0001	3.30(1.89-5.76)	0(0.70)	0/6/2	<0.001	3.12(1.75-5.56)	<0.001	3.12(1.75-5.56)	N	0(0.703)	(1.46-6.66)	**0.52**	$p > 0.1$	Suggestive
Xin et al. 2016	IFN-α + dacarbazine vs dacarbazine	Cutaneous malignant melanoma	8	438/357	Partial response	RR	F	0.76	1.05(0.74-1.50)	0(1.00)	0/8/0	0.765	1.05(0.74-1.50)	0.765	1.05(0.74-1.50)	Y	0(0.997)	(0.68-1.63)	**0.71**	$p > 0.1$	No association
**Follicular lymphoma**																					
Baldo et al. 2010	IFN-α maintenance therapy + CT vs CT or obs	Follicular lymphoma	8	794/769	OS	HR	R	0.16	0.82(0.63-1.08)	52(0.04)	0/6/2	0.008	0.79(0.66-0.94)	0.158	0.82(0.63-1.08)	N	52.58(0.039)	(0.39-1.73)	**0.51**	NA	No association
Baldo et al. 2010	IFN-α maintenance therapy + CT vs CT or obs	Follicular lymphoma	6	686/671	PFS	HR	F	<0.00001	0.66(0.57-0.77)	67.4(0.08)	0/3/3	<0.001	0.66(0.57-0.77)	<0.001	0.65(0.54-0.79)	N	30.84(0.204)	(0.42-1.02)	**0.14**	$p > 0.1$	Suggestive
Baldo et al. 2010	IFN-α maintenance therapy + CT vs CT or obs	Follicular lymphoma	3	273/266	Response rate	RR	R	0.19	1.12(0.95-1.33)	75(0.02)	0/2/1	0.073	1.07(0.99-1.14)	0.159	1.12(0.96-1.31)	Y	70.35(0.034)	(0.19-6.53)	**0.41**	$p > 0.1$	No association
**Non-small cell lung cancer**																					
Rossi et al. 2010	IFN-α maintenance or consolidation therapy vs *p* or obs	NSCLC	4	253/235	OS	HR	F	0.02	0.78(0.64-0.96)	0(0.54)	0/3/1	0.016	0.78(0.64-0.96)	0.016	0.78(0.64-0.96)	Y	0(0.538)	(0.50-1.22)	**0.51**	$p > 0.1$	Suggestive
Rossi et al. 2010	IFN-α maintenance or consolidation therapy vs *p* or obs	NSCLC	2	78/80	PFS	HR	F	0.32	0.84(0.59-1.19)	36.1(0.21)	0/2/0	0.319	0.84(0.59-1.19)	0.355	0.78(0.46-1.32)	Y	36.36(0.21)	-	-	$p > 0.1$	No association
**Renal cell carcinoma**																					
Canil et al. 2010	IFN-α vs CT	Inoperable RCC, metastatic or advanced	6	472/475	Mortality	HR	R	0.001	0.79(0.69-0.91)	1.6(0.41)	0/4/2	0.001	0.79(0.69-0.91)	0.001	0.79(0.69-0.91)	Y	4.35(0.389)	(0.64-0.99)	**0.65**	$p > 0.1$	Suggestive
Canil et al. 2010	IFN-α vs CT	Inoperable RCC, metastatic or advanced	7	493/496	Response	OR	R	<0.00001	6.87 (3.29-14.35)	0(0.64)	0/3/4	<0.001	6.87 (3.29-14.35)	<0.001	6.87 (3.29-14.35)	Y	0(0.651)	(2.61-18.05)	**0.47**	$p > 0.1$	Suggestive
Unverzagt et al. 2017	IFN-α monotherapy vs standard targeted therapy	Metastatic RCC	2	582/584	OS	HR	R	0.001	1.28(1.11-1.49) (Control therapy favored)	0% (0.46)	1/1/0	0.001	1.28(1.10-1.49) (Control therapy favored)	0.001	1.28(1.10-1.49) (Control therapy favored)	N	0(0.453)	-	-	$p > 0.1$	Weak (Control therapy favored)
Unverzagt et al. 2017	IFN-α monotherapy vs standard targeted therapy	Metastatic RCC	2	582/584	1-year mortality	RR	R	0.00033	1.30(1.13-1.51) (Control therapy favored)	0% (0.68)	1/1/0	<0.001	1.30(1.13-1.51) (Control therapy favored)	<0.001	1.30(1.13-1.51) (Control therapy favored)	N	0(0.698)	-	-	$p > 0.1$	Weak (Control therapy favored)
Unverzagt et al. 2017	IFN-α monotherapy vs standard targeted therapy	Metastatic RCC	2	582/584	PFS	HR	R	<0.00001	2.23(1.79-2.77) (Control therapy favored)	0% (0.38)	2/0/0	<0.001	2.23(1.79-2.76) (Control therapy favored)	<0.001	2.23(1.79-2.76) (Control therapy favored)	N	0(0.376)	-	-	$p > 0.1$	Weak (Control therapy favored)
Unverzagt et al. 2017	IFN-α monotherapy vs standard targeted therapy	Metastatic RCC	2	480/527	Tumor remission	RR	R	0.011	0.30(0.12-0.75) (Control therapy favored)	73% (0.05)	1/1/0	<0.001	0.25(0.16-0.37) (Control therapy favored)	0.010	0.30(0.12-0.75) (Control therapy favored)	N	73.27(0.053)	-	-	$p > 0.1$	Weak (Control therapy favored)
**Interleukin-2**																					
**Colorectal cancer**																					

Author, year	Comparison	Cancer type	RCT N.	Interven-tion /control	Outcome	Metrics	M *	Reported *p-Value* *	Reported SE (95% CI) *	Reported I2(%) (*p-Value*) *	R/N/S †	F *p-Value* §	F SE (95% CI) §	R *p-Value* §	R SE (95% CI) §	C ‡	I2(%) (*p-Value*) §	95% Prediction Interval	Egger *p-Value*	Excess Significance	Level of Evidence
Roviello et al. 2016	IL-2 + CTx vs CTx	Colorectal cancer	4	153/150	Objective response	RR	F	0.005	1.60(1.15-2.22)	0(0.70)	0/3/1	0.003	1.65(1.19-2.28)	0.003	1.65(1.19-2.28)	N	0(0.709)	(0.81-3.36)	**0.60**	*p* > 0.1	Suggestive
**Acute myeloid leukemia**																					
Buyse et al. 2011	IL-2 remission maintenance monotherapy vs obs	Acute myeloid leukemia	6	725/730	OS	HR	Mixed	0.458	1.06(0.91-1.24)	NA (0.524)	0/6/0	0.843	1.01(0.95-1.06)	0.843	1.01(0.95-1.06)	Y	0(0.821)	(0.93-1.09)	**0.39**	*p* > 0.1	No association
		Acute myeloid leukemia in first complete remission	6	725/730	Leukemia-free survival	HR	Mixed	0.524	0.96(0.84-1.10)	NA (0.741)	0/6/0	0.624	0.99(0.95-1.03)	0.624	0.99(0.95-1.03)	Y	0(0.613)	(0.94-1.05)	**0.48**	*p* > 0.1	No association
Mao et al. 2015	IL-2 remission maintenance monotherapy vs obs	Acute myeloid leukemia in first complete remission	5	672/683	OS	HR	R	0.35	1.05(0.95-1.16)	0(0.53)	0/5/0	0.347	1.05(0.95-1.16)	0.347	1.05(0.95-1.16)	Y	0(0.53)	(0.89-1.24)	**0.39**	NA	No association
			6	712/714	Disease-free survival	HR	R	0.37	0.95(0.86-1.06)	0(0.78)	0/6/0	0.347	0.95(0.86-1.05)	0.347	0.95(0.86-1.05)	Y	0(0.783)	(0.82-1.10)	**0.66**	NA	No association
**Melanoma**																					
Hamm et al. 2008	IL-2 + IFN-α + CTx vs CTx	Metastatic malignant melanoma	5	364/365	OS	HR	R	0.64	0.95(0.78-1.17)	27.3(0.24)	0/5/0	0.475	0.94(0.80-1.11)	0.642	0.95(0.78-1.16)	Y	26.11(0.247)	(0.59-1.55)	**0.09**	NA	No association
			6	568/566	Overall response	RR	R	<0.0001	1.52(1.24-1.87)	0(0.69)	0/5/1	<0.001	1.52(1.24-1.87)	<0.001	1.52(1.24-1.87)	N	0(0.691)	(1.14-2.04)	**0.19**	*p* > 0.1	Suggestive
			3	250/253	Disease progression rate	HR	R	0.06	0.80(0.63-1.01)	35.7(0.21)	0/2/1	0.013	0.79(0.65-0.95)	0.058	0.80(0.63-1.01)	N	36.81(0.205)	(0.09-7.42)	**0.23**	NA	No association
Ives et al. 2007	IL-2 + IFN-α + CTx vs CTx	Metastatic melanoma	7	607/599	OS	OR	NA	0.9	0.99(0.87-1.13)	NA (0.2)	0/7/0	0.576	1.08(0.82-1.44)	0.729	1.07(0.74-1.55)	N	35.66(0.156)	(0.46-2.50)	**0.96**	*p* > 0.1	No association
			7	527/564	Overall response	OR	NA	0.0001	0.58(0.44-0.77)	NA (0.5)	0/6/1	<0.001	0.58(0.44-0.77)	<0.001	0.58(0.44-0.77)	N	33(0.176)	(0.40-0.84)	**0.17**	*p* > 0.1	Suggestive
			7	527/564	Complete response	OR	NA	0.8	0.93(0.52-1.65)	NA (0.5)	0/7/0	0.880	0.95(0.52-1.75)	0.880	0.95(0.52-1.75)	N	0(0.517)	(0.43-2.12)	**0.24**	*p* > 0.1	No association
			7	527/564	Partial response	OR	NA	0.00007	0.55(0.41-0.74)	NA (0.6)	0/4/3	<0.001	0.55(0.41-0.74)	<0.001	0.55(0.41-0.74)	Y	12.54(0.334)	(0.37-0.82)	**0.08**	*p* > 0.1	Weak
Sasse et al. 2013	IL-2 + IFN-α + CTx vs CTx	Metastatic malignant melanoma	4	417/412	OS	peto OR	F	0.76	0.96(0.74-1.24)	32(0.22)	0/4/0	0.718	1.06(0.76-1.49)	0.547	1.17(0.70-1.94)	Y	37.21(0.189)	(0.19-7.29)	**0.44**	*p* > 0.1	No association
			6	400/395	1-year survival	RR	R	0.8	0.97(0.79-1.20)	42(0.13)	2/4/0	0.920	0.99(0.85-1.16)	0.801	0.97(0.79-1.20)	Y	41.86(0.126)	(0.56-1.68)	**0.18**	*p* > 0.1	No association
			5	365/365	2-year survival	RR	R	0.86	0.97(0.65-1.43)	32(0.21)	0/5/0	0.978	1.00(0.74-1.36)	0.864	0.97(0.65-1.43)	Y	31.76(0.21)	(0.35-2.65)	**0.23**	*p* > 0.1	No association
			7	536/367	Overall response	RR	F	0.00023	1.46(1.19-1.79)	0.0(0.59)	0/6/1	<0.001	1.46(1.19-1.79)	<0.001	1.46(1.19-1.79)	N	0(0.592)	(1.12-1.90)	**0.03**	*p* > 0.1	Weak
**Renal cell carcinoma**																					
Hotte et al. 2007	IL-2 based regimen + CTx vs CT or IFN	Unresectable or metastatic RCC	2	319/100	1-year mortality	RR	R	0.003	0.56(0.38-0.82)	21.0(0.26)	0/0/2	0.001	0.57(0.42-0.79)	0.003	0.56(0.38-0.82)	Y	19.15(0.266)	-	-	*p* > 0.1	Weak
	IL-2 based regimen without CTx vs CT or IFN	Unresectable or metastatic RCC	4	417/262	1-year mortality	RR	R	0.06	1.20(1.00-1.44)	0(0.81)	0/4/0	0.056	1.20(1.00-1.44)	0.056	1.20(1.00-1.44)	Y	0(0.818)	(0.80-1.79)	**0.57**	*p* > 0.1	No association
**Solid tumor**																					
Roviello et al. 2017	IL-2 + CTx vs CTx	NSCLC or head-and-neck cancer	4	181/174	Objective response	RR	F	0.24	1.24(0.87-1.77)	0(0.45)	0/4/0	0.460	1.12(0.83-1.52)	0.460	1.12(0.83-1.52)	Y	0(0.524)	(0.58-2.17)	**0.12**	*p* > 0.1	No association
**Other interferons or cytokines not specified**																					
**Small-cell lung cancer**																					

Author, year	Comparison	Cancer type	RCT N.	Interven-tion /control	Outcome	Me-t-rics	M *	Reported p-Value *	Reported SE (95% CI) *	Reported I2(%) (p-Value) *	R/N/S †	F p-Value §	F SE (95% CI) §	R p-Value §	R SE (95% CI) §	C ‡	I2(%) (p-Value) §	95% Prediction Interval	Egger p-Value	Excess Signif-i-cance	Level of Evidence
Rossi et al. 2010	IFN-γ maintenance or consolidation therapy vs *p* or obs	Small-cell lung cancer	2	116/111	OS	HR	F	0.54	1.09(0.82-1.46)	0(0.60)	0/2/0	0.538	1.09(0.82-1.46)	0.538	1.09(0.82-1.46)	N	0(0.61)	-	-	*p* > 0.1	No association
			2	116/111	PFS	HR	F	0.96	1.01(0.75-1.34)	0(0.45)	0/2/0	0.945	1.01(0.76-1.35)	0.945	1.01(0.76-1.35)	Y	0(0.453)	-	-	*p* > 0.1	No association
**Renal cell carcinoma**																					-
Scherr et al. 2011	Adjuvant IL-2 or interferon vs *p* or obs	Locally advanced, operated RCC	3	420/420	OS	HR	F	0.23	1.18(0.90-1.56)	0(0.48)	0/3/0	0.133	1.16(0.96-1.41)	0.133	1.16(0.96-1.41)	Y	0(0.56)	(0.33-4.14)	**0.21**	*p* > 0.1	No association
			3	420/420	PFS	HR	F	0.48	1.13(0.80-1.60)	40(0.19)	0/3/0	0.359	1.13(0.87-1.47)	0.491	1.13(0.80-1.59)	Y	40.52(0.186)	(0.04-31.18)	**0.96**	NA	No association
Massari et al. 2013	Adjuvant IL-2 or interferon vs *p* or obs	Resected RCC	5	1174 total	2-year OS	RR	R	0.059	1.24(0.99-1.55)	NA (0.55)	0/5/0	0.059	1.24(0.99-1.55)	0.059	1.24(0.99-1.55)	Y	0(0.555)	(0.86-1.78)	**0.40**	NA	No association
			5	1174 total	2-year relapse-free survival	RR	R	0.522	1.05(0.90-1.22)	NA (0.46)	0/5/0	0.521	1.05(0.90-1.22)	0.521	1.05(0.90-1.22)	Y	0(0.467)	(0.82-1.34)	**0.56**	NA	No association
			5	1241 total	5-year OS	RR	R	0.415	1.09(0.88-1.35)	NA (0.09)	1/4/0	0.351	1.07(0.93-1.22)	0.416	1.09(0.88-1.35)	Y	50.33(0.09)	(0.58-2.07)	**0.52**	NA	No association
			**5**	**1352 total**	**5-year relapse-free survival**	**RR**	**R**	**0.428**	**1.06(0.92-1.21)**	**NA (0.29)**	**0/5/0**	**0.427**	**1.05(0.93-1.18)**	**0.426**	**1.06(0.92-1.20)**	**Y**	**18.36(0.298)**	**(0.78-1.42)**	**0.39**	**NA**	**No association**

Abbreviations: RCT, randomized controlled trial; *N.*, number; SE, standard effect; CI, confidence interval; M, model; F, fixed effect; R, random effect; NA, not available; C, concordance with largest study; Y, concordant with largest study; N, not concordant with largest study; OS, overall survival; PFS progression-free survival; RR, risk ratio; HR, hazard ratio; OR, odds ratio; IFN-*α*, interferon alpha; 5FU, fluorouracil; IFN, interferon; *p*, placebo; CTx, chemotherapy; CT, conventional therapy; obs, observation; VAX, cancer vaccine; IL-2, interleukin; IFN-*γ*, interferon gamma; AT, adjuvant therapy; HCC, hepatocellular carcinoma; NSCLC, non-small cell lung cancer; RCC, renal cell carcinoma. * Value reported in original article of the meta-analysis. † Number of individual studies of effect size with statistical significance in the reverse direction/not statistically significant/statistically significant. ‡ Concordance of fixed and random effects summary outcome with outcome of largest individual study. § Value obtained from re-analysis of original meta-analysis. ES is estimated by assuming power of each study could be replaced by power of the study with most cases and controls. All *p-Value*s are two-sided.

Table S6. Umbrella review summary and level of evidence reported in meta-analyses of cancer therapeutic vaccines.

Author, year	Comparison	Cancer type	RCT N.	Interven-tion /control	Outcome	Me-t-rics	M *	Reported p-Value *	Reported SE (95% CI) *	Reported I2(%) (p-Value) *	R/N/S †	F p-Value §	F SE (95% CI) §	R p-Value §	R SE (95% CI) §	C ‡	I2(%) (p-Value) §	95% Prediction Interval	Egger p-Value	Excess Signif-i-cance	Level of Evidence
**Sipuleucel-T**																					
**Glioma**																					
Cao et al. 2014	DC vs non-DC	High-grade glioma	2¶	43/41	1-year OS	OR	-	-	-	-	0/3/0	0.096	2.43(0.85-6.93)	0.096	2.43(0.85-6.93)	Y	0(0.848)	(0.00-2157.51)	**0.27**	*p* > 0.1	No association
			3¶	25/25	1.5-year OS	OR	-	-	-	-	0/2/0	0.067	3.24(0.92-11.41)	0.067	3.24(0.92-11.41)	Y	0(0.692)	-	-	*p* > 0.1	No association
			3¶	44/42	2-year OS	OR	-	-	-	-	0/3/0	0.038	3.41(1.07-10.81)	0.038	3.41(1.07-10.81)	N	0(0.974)	-	-	*p* > 0.1	Weak

Author, year	Comparison	Cancer type	RCT *N.*	Intervention/control	Outcome	Metrics	M *	Reported *p-Value* *	Reported SE (95% CI) *	Reported I2(%) (*p-Value*) *	R/N/S †	F *p-Value* §	F SE (95% CI) §	R *p-Value* §	R SE (95% CI) §	C ‡	I2(%) (*p-Value*) §	95% Prediction Interval	Egger *p-Value*	Excess Significance	Level of Evidence
**Prostate cancer**																					
Kawalec et al. 2012	Sipuleucel-T vs *p*	Castration-resistant prostate cancer	3	488/249	OS	HR	F	0.001	0.73(0.61-0.88)	0(0.49)	0/1/2	0.001	0.73(0.60-0.88)	<0.001	0.73(0.6-0.88)	Y	0(0.465)	(0.22-2.44)	**0.71**	*p* > 0.1	Suggestive
			3	488/249	Time to progression	HR	F	0.17	0.89(0.75-1.05)	6(0.35)	0/3/0	0.165	0.89(0.75-1.05)	0.170	0.88(0.74-1.05)	Y	3.94(0.353)	(0.26-3.02)	**0.57**	*p* > 0.1	No association
**Other cancer vaccines**																					
**Non-small cell lung cancer**																					
Ding et al. 2014	VAX(Tecemotide or EGF vaccine or SRL172 or TG4010 or MAGE-A3 or L-BLP25) vs CTx or obs or α-tocopherol	NSCLC	6	1363/876	OS	OR	F	0.0002	0.83(0.76-0.91)	0(0.67)	0/4/2	0.001	0.56(0.39-0.79)	0.001	0.56(0.39-0.79)	Y	64.95(0.014)	(0.34-0.92)	**0.99**	*p* > 0.1	Weak
Wang et al. 2015	VAX vs CTx or *p* or obs	NSCLC	7	1332/954	1-year OS	OR	F	0.0004	1.65(1.25-2.18)	31(0.19)	0/5/2	<0.001	1.51(1.24-1.83)	<0.001	1.65(1.25-2.18)	N	56.05(0.034)	(0.87-3.13)	**0.06**	*p* > 0.1	Weak
			3	1004/582	2-year OS	OR	F	0.03	1.64(1.04-2.59)	48(0.14)	0/2/1	0.004	1.40(1.11-1.76)	0.033	1.64(1.04-2.59)	N	52.43(0.122)	(0.02-175.81)	**0.06**	*p* > 0.1	Weak
			2	917/493	3-year OS	OR	R	0.19	1.69(0.78-3.65)	80(0.03)	0/1/1	0.020	1.36(1.05-1.76)	0.186	1.69(0.78-3.65)	N	74.32(0.048)	-	-	*p* > 0.1	No association
			4	446/403	Objective response	OR	F	0.05	1.37(0.99-1.90)	15(0.32)	0/3/1	0.041	1.35(1.01-1.79)	0.054	1.37(0.99-1.90)	N	26.32(0.254)	(0.55-3.41)	**0.21**	*p* > 0.1	No association
Yu et al. 2017	VAX vs CTx or *p*	Advanced NSCLC	3	1014/595	PFS	OR	R	0.01	1.31(1.05-1.63)	0(0.78)	0/1/2	0.015	1.31(1.05-1.63)	0.015	1.31(1.05-1.63)	Y	48.77(0.142)	(0.32-5.33)	**0.62**	*p* > 0.1	Suggestive
Zhou et al. 2016	VAX(EGF vaccine or BLP-25 or TG4010, etc) vs CTx	Advanced NSCLC	4	300/302	OS	HR	R	NA	0.76(0.60-0.92)	0.0(0.821)	0/4/0	0.012	0.77(0.63-0.94)	0.012	0.77(0.63-0.94)	N	0(0.796)	(0.50-1.20)	**0.55**	*p* > 0.1	Weak
	VAX(EGF vaccine or BLP-25 or TG4010, etc) vs *p*	Advanced NSCLC	5	1314/884	OS	HR	R	NA	0.81(0.71-0.91)	25.6(0.251)	0/4/1	0.001	0.83(0.75-0.92)	0.002	0.83(0.74-0.93)	N	16.86(0.307)	(0.64-1.07)	**0.20**	*p* > 0.1	Suggestive
**Renal cell carcinoma**																					
Massari et al. 2013	Adjuvant VAX vs no AT	Resected RCC	2	673 total	5-year relapse-free survival	RR	R	0.765	0.91(0.50-1.66)	NA (0.055)	0/1/1	0.077	0.80(0.63-1.02)	0.765	0.91(0.50-1.66)	N	75.99(0.041)	-	-	NA	No association
Scherr et al. 2011	Adjuvant VAX vs *p* or obs	Locally advanced, operated RCC	2	421/427	OS	HR	F	0.89	1.02(0.75-1.39)	0(0.42)	0/2/0	0.765	1.04(0.80-1.35)	0.765	1.04(0.80-1.35)	Y	0(0.452)	-	-	*p* > 0.1	No association
			2	421/427	PFS	HR	F	0.68	0.95(0.76-1.19)	0(0.35)	0/2/0	0.667	0.95(0.76-1.19)	0.667	0.95(0.76-1.19)	Y	0(0.347)	-	-	NA	No association
Unverzagt et al. 2017	VAX + CT vs CT	Metastatic RCC	2	569/502	OS	HR	R	0.14	1.14(0.96-1.37)	22(0.26)	0/2/0	0.137	1.15(0.96-1.37)	0.174	1.16(0.94-1.43)	Y	21.92(0.258)	-	-	NA	No association
			2	546/488	1-year mortality	RR	R	0.34	1.10(0.91-1.32)	0.0(0.43)	0/0/2	0.339	1.10(0.91-1.32)	0.339	1.10(0.91-1.32)	N	0(0.428)	-	-	*p* > 0.1	No association
			**2**	**569/502**	**Tumour remission**	**RR**	**R**	**0.45**	**0.93(.0.76-1.13)**	**0.0(0.78)**	**0/2/0**	**0.451**	**0.93(0.76-1.13)**	**0.451**	**0.93(0.76-1.13)**	**Y**	**0(0.787)**	-	-	***p* > 0.1**	**No association**

Abbreviations: RCT, randomized controlled trial; *N.*, number; SE, standard effect; CI, confidence interval; M, model; F, fixed effect; R, random effect; NA, not available; C, concordance with largest study; Y, concordant with largest study; N, not concordant with largest study; OS, overall survival; PFS progression-free survival; RR, risk ratio; HR, hazard ratio; OR, odds ratio; DC, dendritic cell based vaccine; P, placebo; VAX, cancer vaccine; CTx, chemotherapy; obs, observation; P, placebo; AT, adjuvant therapy; CT, conventional therapy; NSCLC, non-small cell lung cancer; RCC, renal cell carcinoma. * Value reported in original article of the meta-analysis. † Number of individual studies of effect size with statistical significance in the reverse direction/not statistically significant/statistically significant.

‡ Concordance of fixed and random effects summary outcome with outcome of largest individual study. § Value obtained from re-analysis of original meta-analysis. Re-analysis was performed after excluding non-RCTs from original meta-analysis. All *p-Value*s are two-sided.

Table S7. Umbrella review summary and level of evidence reported in meta-analyses of uncategorized immunotherapy.

Author, year	Comparison	Cancer type	RCT *N.*	Interven-tion /control	Outcome	Me-trics	M *	Reported *p-Value* *	Reported SE(95% CI) *	Reported I2(%) (*p-Value*) *	R/N/S †	F *p-Value* §	F SE (95% CI) §	R *p-Value* §	R SE (95% CI) §	C ‡	I2(%) (*p-Value*) §	95% Prediction Interval	Egger *p-Value*	Excess Signifi-cance	Level of Evidence
**Hepatocellular carcinoma**																					
Zhu et al. 2015	Adjuvant immunotherapy vs no AT with backbone curative resection	HCC	2	160/117	1-year OS	HR	R	NA	1.51(0.47-4.90)	NA	0/2/0	0.186	1.04(0.98-1.10)	0.186	1.04(0.98-1.10)	Y	0(0.688)	-	-	*p* > 0.1	No association
			2	160/117	5-year OS	HR	R	NA	1.05(0.64-1.73)	NA	0/2/0	0.849	1.03(0.75-1.42)	0.849	1.03(0.75-1.42)	Y	0(0.895)	-	-	*p* > 0.1	No association
			2	160/117	Overall recurrence	OR	R	NA	0..30(0.15-0.63)	NA	0/0/2	<0.001	0.31(0.18-0.53)	0.001	0.30(0.15-0.63)	Y	81.57(0.02)	-	-	*p* > 0.1	Weak
**Melanoma**																					
Wu et al. 2017	Additional mAB or VAX vs no additional therapy, with backbone therapy in both arms	Metastatic melanoma	3	1423 total	OS	HR	R	NA	0.89(0.74-1.05)	51.2(0.129)	0/2/1	0.054	0.89(0.80-1.00)	0.222	0.90(0.75-1.07)	Y	54.47(0.111)	(0.14-5.64)	**0.98**	NA	No association
			4	2368 total	PFS	HR	R	NA	0.88(0.63-1.13)	93.2(0.000)	0/3/1	0.003	0.90(0.83-0.96)	0.280	0.86(0.66-1.13)	N	92.27(< 0.001)	(0.24-3.11)	**0.56**	NA	No association
			4	2368 total	Overall response	RR	R	NA	1.21(0.66-2.23)	85.6(0.000)	0/3/1	<0.001	1.66(1.35-2.03)	0.525	1.22(0.66-2.24)	N	85.38(< 0.001)	(0.08-19.49)	**0.48**	NA	No association
**Non-small cell lung cancer**																					
Dammeijer et al. 2016	Immunotherapy (L-BLP25 or DC or CIK or DC/CIK or IL-2 or etc) without CTx vs CT or *p*	NSCLC	9	5033 total	OS	HR	R	0.01	0.81(0.70-0.94)	43.9(NA)	0/7/2	<0.001	0.84(0.76-0.92)	0.005	0.81(0.70-0.94)	N	42.53(0.084)	(0.56-1.18)	**0.15**	NA	Weak
			10	5164 total	PFS	HR	R	0.006	0.83(0.72-0.95)	57.7(NA)	0/6/4	0.002	0.89(0.82-0.96)	0.006	0.83(0.72-0.95)	N	57.02(0.013)	(0.56-1.21)	**0.01**	NA	Weak
Wang et al. 2012	Immunotherapy (IL-2 or CIK or DC/CIK or GM-CSF or trastuzumab or cetuximab or VAX) vs CT	Advanced NSCLC	11	1519/1514	OS	HR	F	0.0007	0.95(0.92-0.98)	0(0.45)	0/10/1	0.002	0.96(0.93-0.98)	0.002	0.96(0.93-0.98)	N	0(0.503)	(0.93-0.99)	**0.01**	*p* < 0.1	Weak
			7	1159/1157	PFS	HR	F	0.0004	1.08(1.03-1.12)	32(0.19)	0/6/1	0.005	1.06(1.02-1.10)	0.058	1.05(1.00-1.10)	N	24.14(0.245)	(0.95-1.16)	**0.09**	*p* > 0.1	No association
			10	1346/1341	Overall response	HR	F	0.003	1.19(1.06-1.34)	2(0.42)	0/8/2	0.006	1.17(1.05-1.31)	0.007	1.17(1.04-1.32)	Y	1.08(0.428)	(1.01-1.35)	**0.17**	*p* > 0.1	Suggestive
			9	1303/1298	Complete response	HR	F	0.97	1.00(0.77-1.31)	0(0.74)	0/9/0	0.851	1.02(0.79-1.33)	0.851	1.02(0.79-1.33)	Y	0(0.739)	(0.73-1.44)	**0.27**	*p* > 0.1	No association
			8	1215/1215	Partial response	HR	F	0.002	1.23(1.08-1.40)	1(0.42)	0/6/2	0.003	1.22(1.07-1.39)	0.004	1.22(1.06-1.39)	Y	0.48(0.425)	(1.03-1.44)	**0.22**	*p* > 0.1	Suggestive
Yu et al. 2017	mAB (anti-CTLA-4 or anti-PD-1/PD-L1 or VEGF-A) vs CTx or *p*	Advanced NSCLC	9	2229/2215	PFS	OR	R	0.0001	2.05(1.42-2.94)	76 (< 0.0001)	0/3/6	<0.001	2.04(1.71-2.42)	<0.001	2.05(1.42-2.94)	N	90.38(< 0.001)	(0.61-6.84)	**0.94**	*p* > 0.1	Suggestive
Zhou et al. 2016	Immunomodulator talactoferrin vs *p*	Advanced NSCLC	2	544/298	OS	HR	R	NA	0.88(0.52-1.24)	74.0(0.050)	0/2/0	0.841	0.98(0.83-1.16)	0.553	0.88(0.58-1.34)	Y	66.92(0.082)	-	-	NA	No association
	Immunomodulator (IL-2 or SRL172 or talactoferrin) + CTx vs CTx	Advanced NSCLC	3	392/376	OS	HR	R	NA	1.01(0.97-1.05)	0.0(0.582)	0/3/0	0.587	1.01(0.97-1.05)	0.587	1.01(0.97-1.05)	Y	0(0.569)	(0.78-1.30)	**0.97**	NA	No association
	Ipilimumab or nivolumab or atezolizumab vs CTx	Advanced NSCLC	3	571/570	OS	HR	R	NA	0.69(0.59-0.80)	7.6(0.339)	0/1/2	<0.001	0.71(0.61-0.81)	<0.001	0.71(0.61-0.82)	Y	1.89(0.361)	(0.27-1.86)	**0.72**	*p* > 0.1	Suggestive

Author, year	Comparison	Cancer type	RCT *N.*	Interven-tion /control	Outcome	Met-rics	M *	Reported *p-Value* *	Reported SE(95% CI) *	Reported I2(%) (*p-Value*) *	R/N/S †	F *p-Value* §	F SE (95% CI) §	R *p-Value* §	R SE (95% CI) §	C ‡	I2(%) (*p-Value*) §	95% Prediction Interval	Egger *p-Value*	Excess Signifi-cance	Level of Evidence
**Renal cell carcinoma**																					
Bai et al. 2017	Postsurgical AIT (IL-2 + IFN-α or IFN-α2b or IFN-α, etc) vs no AT	Locally advanced RCC	5	941/902	OS	HR	F	0.345	1.08(0.92-1.28)	0.0(0.682)	0/5/0	0.345	1.08(0.92-1.28)	0.345	1.08(0.92-1.28)	Y	0(0.682)	(0.83-1.42)	**0.32**	NA	No association
			5	941/902	DFS	HR	F	0.477	1.05(0.91-1.21)	6.6(0.369)	0/5/0	0.477	1.05(0.91-1.21)	0.459	1.06(0.91-1.23)	Y	6.6(0.369)	(0.79-1.41)	**0.40**	NA	No association
**Solid tumor**																					
Wu et al. 2017	Additional mAB or VAX vs no additional therapy with backbone therapy	Lung cancer or melanoma	6¶	1936 total	OS	HR	-	-	-	-	0/5/1	0.030	0.90(0.81-0.99)	0.043	0.90(0.81-1.00)	Y	8.22(0.364)	(0.74-1.08)	**0.75**	NA	Weak
			5¶	2547 total	PFS	HR	-	-	-	-	0/3/2	<0.001	0.87(0.82-0.93)	0.122	0.84(0.67-1.05)	N	90.6(0)	(0.35-1.98)	**0.38**	NA	No association
			**7¶**	**2881 total**	**Overall response**	**RR**	-	-	-	-	**0/5/2**	**<0.001**	**1.64(1.38-1.96)**	**0.076**	**1.46(0.96-2.22)**	**N**	**78.05(0)**	**(0.38-5.67)**	**0.37**	**NA**	**No association**

Abbreviations: RCT, randomized controlled trial; *N*., number; SE, standard effect; CI, confidence interval; M, model; F, fixed effect; R, random effect; NA, not available; C, concordance with largest study; Y, concordant with largest study; N, not concordant with largest study; OS, overall survival; PFS progression-free survival; RR, risk ratio; HR, hazard ratio; OR, odds ratio; AT, adjuvant therapy; mAB, monoclonal antibodies; VAX, cancer vaccine; IL-2, interleukin-2; CIK, cytokine-induced killer cells; DC/CIK, dendritic cells with cytokine-induced killer cells; GM-CSF, Granulocyte-macrophage colony-stimulating factor; DC, dendritic cell; *p*, placebo; IFN, interferon; HCC, hepatocellular carcinoma; NSCLC, non-small cell lung cancer. * Value reported in original article of the meta-analysis. † Number of individual studies of effect size with statistically significant in reverse direction/not statistically significant/statistically significant. ‡ Concordance of fixed and random effects summary outcome with outcome of largest individual study. § Value obtained from re-analysis of original meta-analysis. Re-analysis was performed after excluding non-RCTs from original meta-analysis. All *p-Value*s are two-sided.

Table S8. Comparison between re-analyses of RCTs and re-analyses of RCTs plus non-RCT(s).

Author, year	Comparison	Cancer type	*N.* of studies	Types of studies	Interven-tion /control	Outcome	Met-rics	M *	Reported *p-Value* *	Reported SE (95% CI) *	Reported I2(%) (*p-Value*) *	R/N/S †	F *p-Value* §	F SE (95% CI) §	R *p-Value* §	R SE (95% CI) §	C‡	I2(%) (*p-Value*) §	95% Prediction Interval	Egger *p-Value*	Excess Signifi-cance	Level of Evidence
Yuan et al. 2017	Postoperative ACI (CIK or LAK + IL-2 or lymphocytes) vs no AT	Pretreated HCC, not advanced	6	RCTs	407/362	1-year mortality	RR	-	-	-	-	0/5/1	0.035	0.50(0.26-0.95)	0.032	0.43(0.20-0.93)	N	17.93(0.297)	(0.09-2.12)	**0.01**	*p* > 0.1	Weak
			8	RCTs and retrospective studies	1003/971			F	<0.0001	0.64(0.52-0.79)	5(0.39)	0/6/2	<0.001	0.67(0.55-0.83)	0.001	0.67(0.53-0.85)	Y	1.96(0.414)	(0.48-0.93)	**0.07**	*p* > 0.1	Weak
			6	RCTs	407/362	2-year mortality	RR	-	-	-	-	0/6/0	0.002	0.52(0.34-0.78)	0.002	0.52(0.34-0.78)	N	0(0.685)	(0.29-0.93)	**0.59**	*p* > 0.1	Weak
			8	RCTs and retrospective studies	1003/971			F	<0.00001	0.72(0.63-0.83)	0(0.48)	0/7/1	<0.001	0.74(0.65-0.85)	<0.001	0.74(0.65-0.85)	Y	0(0.49)	(0.62-0.87)	**0.04**	*p* > 0.1	Weak
			6	RCTs	407/362	3-year mortality	RR	-	-	-	-	0/5/1	0.009	0.71(0.55-0.92)	0.009	0.71(0.55-0.92)	N	0(0.593)	(0.49-1.02)	**0.81**	*p* > 0.1	Suggestive
			8	RCTs and retrospective studies	1003/971			F	<0.00001	0.73(0.65-0.81)	0(0.74)	0/5/3	<0.001	0.73(0.65-0.81)	<0.001	0.73(0.65-0.81)	Y	0(0.741)	(0.63-0.84)	**0.56**	*p* > 0.1	Convincing
			2	RCTs	160/117	5-year mortality	RR	-	-	-	-	0/2/0	0.920	0.99(0.83-1.19)	0.920	0.99(0.83-1.19)	Y	0(0.966)	-	-	*p* > 0.1	No association
			4	RCTs and retrospective studies	756/726			F	0.0005	0.86(0.79-0.94)	23(0.27)	0/2/2	0.001	0.86(0.79-0.94)	0.011	0.87(0.78-0.97)	Y	23.42(0.271)	(0.62-1.22)	**0.63**	*p* > 0.1	Suggestive

Author, year	Comparison	Cancer type	*N.* of studies	Types of studies	Interven-tion /control	Outcome	Me-trics	M *	Reported *p-Value* *	Reported SE (95% CI) *	Reported I2(%) (*p-Value*) *	R/N/S †	F *p-Value* §	F SE (95% CI) §	R *p-Value* §	R SE (95% CI) §	C‡	I2(%) (*p-Value*) §	95% Prediction Interval	Egger *p-Value*	Excess Significance	Level of Evidence
Zhao et al. 2017	ACI(LAK + IL-2 or DC/CIK or CIK or AKT or TIL) vs CT	NSCLC, operated or non-operated	8	RCTs	483/432	1-year recurrence rate	RR	-	-	-	-	0/6/2	<0.001	0.54(0.42-0.71)	<0.001	0.54(0.42-0.71)	N	0(0.495)	(0.39-0.75)	**0.41**	*p* > 0.1	Suggestive
			10	RCTs and retrospective studies	1079/1041			R	<0.00001	0.77(0.69-0.86)	56(0.02)	0/7/3	<0.001	0.80(0.72-0.89)	<0.001	0.64(0.50-0.82)	N	54.65(0.019)	(0.33-1.23)	**0.01**	*p* > 0.1	Weak
			6	RCTs	407/362	2-year recurrence rate	RR	-	-	-	-	0/3/3	<0.001	0.62(0.51-0.75)	<0.001	0.62(0.51-0.75)	Y	0(0.426)	(0.48-0.81)	**0.80**	*p* > 0.1	Suggestive
			8	RCTs and retrospective studies	1003/971			R	<0.00001	0.79(0.73-0.86)	60(0.01)	0/3/5	<0.001	0.80(0.74-0.87)	<0.001	0.70(0.59-0.84)	Y	59.7(0.015)	(0.44-1.13)	**0.03**	*p* > 0.1	Weak
			6	RCTs	407/362	3-year recurrence rate	RR	-	-	-	-	0/5/1	0.001	0.81(0.72-0.92)	0.001	0.81(0.72-0.92)	N	0(0.772)	(0.68-0.97)	**0.30**	*p* > 0.1	Suggestive
			8	RCTs and retrospective studies	1003/971			F	<0.00001	0.85(0.79-0.91)	0(0.50)	0/5/3	0.001	0.84(0.79-0.90)	<0.001	0.84(0.79-0.90)	Y	0(0.507)	(0.78-0.91)	**0.17**	*p* > 0.1	Convincing
			2	RCTs	160/117	5-year recurrence rate	RR	-	-	-	-	0/2/0	0.110	0.92(0.83-1.02)	0.110	0.92(0.83-1.02)	Y	0(0.58)	-	-	*p* > 0.1	No association
			4	RCTs and retrospective studies	756/726			F	0.0004	0.90(0.85-0.95)	0(0.69)	0/2/2	<0.001	0.89(0.85-0.95)	<0.001	0.89(0.85-0.95)	Y	0(0.699)	(0.79-1.01)	**0.99**	*p* > 0.1	Suggestive
			13	RCTs	718/844	1-year OS	RR	-	-	-	-	0/8/5	<0.001	1.15(1.10-1.21)	<0.001	1.19(1.11-1.27)	Y	25.14(0.19)	(1.03-1.37)	**0.01**	*p* > 0.1	Weak
			15	RCTs and observation studies/prospective cohort	779/907			R	0.001	1.16(1.06–1.26)	62.9(0.001)	0/9/6	<0.001	1.14(1.09-1.19)	<0.001	1.17(1.10-1.24)	Y	32.35(0.11)	(1.00-1.36)	**0.02**	*p* > 0.1	Weak
			11	RCTs	669/755	2-year OS	RR	-	-	-	-	0/5/6	<0.001	1.43(1.30-1.58)	<0.001	1.43(1.30-1.58)	Y	0(0.562)	(1.28-1.61)	**0.24**	*p* > 0.1	Convincing
			13	RCTs and observation studies/prospective cohort	730/818			R	<0.001	1.38(1.24–1.55)	35.4(0.099)	0/6/7	<0.001	1.33(1.23-1.45)	<0.001	1.35(1.20-1.51)	Y	33.88(0.111)	(1.02-1.78)	**0.24**	*p* > 0.1	Convincing
			8	RCTs	529/613	3-year OS	RR	-	-	-	-	0/5/3	<0.001	1.45(1.24-1.69)	<0.001	1.45(1.24-1.69)	N	0(0.639)	(1.19-1.76)	**0.31**	*p* > 0.1	Suggestive
			10	RCTs and observation studies/prospective cohort	590/676			F	<0.001	1.42(1.24–1.61)	11.4(0.337)	0/6/4	<0.001	1.32(1.18-1.47)	<0.001	1.32(1.17-1.49)	N	7.37(0.374)	(1.09-1.60)	**0.24**	*p* > 0.1	Suggestive
			5	RCTs	400/485	5-year OS	RR	-	-	-	-	0/2/3	<0.001	1.71(1.39-2.11)	0.031	1.67(1.05-2.67)	N	77.03(0.002)	(0.32-8.72)	**0.87**	*p* > 0.1	Suggestive
			6	RCTs and observation studies/prospective cohort	419/506			R	0.032	1.56(1.04–2.33)	75.5(0.01)	0/3/3	<0.001	1.59(1.31-1.92)	0.032	1.56(1.04-2.33)	N	75.45 (< 0.001)	(0.41-5.85)	**0.84**	*p* > 0.1	Suggestive
			4	RCTs	187/229	1-year PFS	RR	-	-	-	-	0/2/2	<0.001	1.46(1.24-1.72)	0.031	1.46(1.24-1.72)	Y	0(0.877)	(1.02-2.09)	**0.16**	*p* > 0.1	Suggestive
			6	RCTs and observation studies/prospective cohort	271/248			F	<0.001	1.40(1.23–1.59)	10.7(0.345)	0/2/3	<0.001	1.32(1.18-1.47)	<0.001	1.32(1.18-1.47)	Y	0(0.493)	(1.10-1.58)	**0.17**	*p* > 0.1	Suggestive
			2	RCTs	137/138	2-year PFS	RR	-	-	-	-	0/1/1	<0.001	1.69(1.29-2.23)	0.027	1.79(1.07-2.99)	N	68.9(0.073)	-	-	*p* > 0.1	Weak
			3	RCTs and observation studies/prospective cohort	179/180			R	0.029	1.53(1.05–2.23)	70.7(0.033)	0/2/1	<0.001	1.42(1.17-1.73)	0.024	1.52(1.06-2.20)	N	68.64(0.041)	(0.02-95.78)	**0.07**	*p* > 0.1	Weak
Su et al. 2016	DC/CIK vs obs with backbone TACE	HCC	3	RCTs	104/106	1-year OS	OR	-	-	-	-	0/3/0	0.027	2.00(1.08-3.70)	0.027	2.00(1.08-3.70)	N	0(0.733)	(0.04-107.12)	**0.12**	*p* > 0.1	Weak
			4	RCTs and controlled clinical trial	145/150			F	0.02	2.00(1.12-3.56)	0(0.89)	0/4/0	0.019	2.00(1.12-3.56)	0.019	2.00(1.12-3.56)	N	0(0.493)	(0.56-7.12)	**0.64**	*p* > 0.1	Weak
			2	RCTs	72/68	2-year OS	OR	-	-	-	-	0/2/0	0.028	2.16(1.09-4.32)	0.028	2.16(1.09-4.32)	N	0(0.685)	-	-	*p* > 0.1	Weak
			3	RCTs and controlled clinical trial	113/112			F	0.04	1.77(1.02-3.07)	0(0.59)	0/3/0	0.042	1.77(1.02-3.08)	0.042	1.77(1.02-3.08)	N	1.95(0.361)	(0.05-63.38)	**0.42**	*p* > 0.1	Weak
			5	RCTs	250/245	Overall response	OR	-	-	-	-	0/5/0	0.046	1.47(1.01-2.16)	0.046	1.47(1.01-2.16)	N	0(0.893)	(0.79-2.73)	**0.47**	*p* > 0.1	Weak
			6	RCTs and controlled clinical trial	291/289			F	0.03	1.51(1.05-2.16)	0(0.94)	0/6/0	0.027	1.50(1.05-2.16)	0.027	1.50(1.05-2.16)	N	0(0.819)	(0.90-2.51)	**0.32**	*p* > 0.1	Weak

Author, year	Comparison	Cancer type	*N.* of studies	Types of studies	Interven-tion /control	Outcome	Me t-rics	M *	Reported *p-Value* *	Reported SE (95% CI) *	Reported I2(%) (*p-Value*) *	R/N/S †	F *p-Value* §	F SE (95% CI) §	R *p-Value* §	R SE (95% CI) §	C‡	I2(%) (*p-Value*) §	95% Prediction Interval	Egger *p-Value*	Excess Signifi-cance	Level of Evidence
Cao et al. 2014	DC vs non-DC	High-grade glioma	5	RCTs	250/245	DCR	OR	-	-	-	-	0/3/2	0.013	1.81(1.13-2.90)	0.033	1.84(1.05-3.24)	Y	17.82(0.302)	(0.35-9.60)	**0.55**	*p* > 0.1	Suggestive
			6	RCTs and controlled clinical trial	291/289			F	0.01	1.81(1.15-2.83)	0(0.45)	0/4/2	0.013	1.79(1.13-2.82)	0.013	1.79(1.13-2.82)	Y	19.05(0.293)	(0.85-3.75)	**0.61**	*p* > 0.1	Suggestive
			4	RCTs	132/127	Quality of life	OR	-	-	-	-	0/2/2	0.001	3.07(1.58-5.97)	0.001	3.07(1.58-5.97)	N	0(0.95)	(0.71-13.23)	**0.38**	*p* > 0.1	Suggestive
			5	RCTs and controlled clinical trial	173/171			F	<0.0001	3.30(1.82-5.98)	0(0.97)	0/2/3	<0.001	3.26(1.79-5.93)	<0.001	3.26(1.79-5.93)	Y	0(0.453)	(1.24-8.61)	**0.33**	*p* > 0.1	Suggestive
			2	RCTs	43/41	1-year OS	OR	-	-	-	-	0/3/0	0.096	2.43(0.85-6.93)	0.096	2.43(0.85-6.93)	Y	0(0.848)	(0.00-2157.51)	**0.27**	*p* > 0.1	No association
			7	RCTs and non-RCTs including historical cohorts	98/256			R	0.0006	2.89(1.58-5.27)	45(0.09)	0/6/1	0.012	2.33(1.21-4.50)	0.023	2.96(1.16-7.55)	N	26.77(0.224)	(0.27-32.68)	**0.02**	*p* > 0.1	Weak
			3	RCTs	25/25	1.5-year OS	OR	-	-	-	-	0/2/0	0.067	3.24 (0.92-11.41)	0.067	3.24 (0.92-11.41)	Y	0(0.692)	-	-	*p* > 0.1	No association
			6	RCTs and non-RCTs including historical cohorts	80/240			R	<0.00001	5.13(2.80-9.41)	10(0.35)	0/2/4	<0.001	4.92(2.63-9.22)	<0.001	5.00(2.57-9.73)	Y	43.46(0.115)	(1.53-16.32)	**0.15**	*p* > 0.1	Suggestive
			3	RCTs	44/42	2-year OS	OR	-	-	-	-	0/3/0	0.038	3.41 (1.07-10.81)	0.038	3.41 (1.07-10.81)	N	0(0.974)	-	-	*p* > 0.1	Weak
			7	RCTs and non-RCTs including historical cohorts	98/256			R	<0.00001	4.69(2.48-8.85)	0(0.50)	0/4/3	<0.001	5.01(2.62-9.59)	<0.001	5.01(2.62-9.59)	Y	0(0.58)	(2.00-12.57)	**0.42**	*p* > 0.1	Suggestive
Wu et al. 2017	Additional mAB or VAX vs no additional therapy, with backbone therapy in both arms	Lung cancer or melanoma	6	RCTs	NA	OS	HR	-	-	-	-	0/5/1	0.030	0.90(0.81-0.99)	0.043	0.90(0.81-1.00)	Y	8.22(0.364)	(0.74-1.08)	**0.75**	NA	Weak
			7	RCTs and open-label study	NA			R	<0.001	0.86(0.78-0.95)	1.2(0.415)	0/6/1	0.010	0.88(0.80-0.97)	0.014	0.88(0.80-0.97)	N	6.25(0.38)	(0.75-1.03)	**0.57**	NA	Weak
			5	RCTs	NA	PFS	HR	-	-	-	-	0/3/2	<0.001	0.87(0.82-0.93)	0.122	0.84(0.67-1.05)	N	90.6(0)	(0.35-1.98)	**0.38**	NA	No association
			6	RCTs and open-label study	NA			F	NA	0.93(0.72-1.14)	92.9(0.000)	1/3/2	0.101	0.95(0.89-1.01)	0.473	0.91(0.72-1.17)	Y	93.54 (< 0.001)	(0.37-2.23)	**0.47**	NA	No association
			7	RCTs	NA	Overall response	RR	-	-	-	-	0/5/2	<0.001	1.64(1.38-1.96)	0.076	1.46(0.96-2.22)	N	78.05(0)	(0.38-5.67)	**0.37**	NA	No association
			8	RCTs and open-label study	NA			R	0.034	1.51(1.03-2.20)	74.9(0.000)	0/6/2	<0.001	1.66(1.40-1.97)	0.033	1.51(1.03-2.20)	Y	74.64 (< 0.001)	(0.45-5.06)	**0.42**	NA	Weak

Abbreviations: RCT, randomized controlled trial; *N.*, number; SE, standard effect; CI, confidence interval; M, model; F, fixed effect; R, random effect; NA, not available; C, concordance with largest study; Y, concordant with largest study; N, not concordant with largest study; OS, overall survival; PFS progression-free survival; RR, risk ratio; HR, hazard ratio; OR, odds ratio; ACI, adoptive cell immunotherapy; CIK, cytokine-induced killer cells; LAK, lymphokine-activated killer cells; IL-2, Interleukin-2; AT, adjuvant therapy; DC/CIK, dendritic cells with cytokine-induced killer cells; AKT, activated killer T-cells; TIL, tumor-infiltrating lymphocytes; CT, conventional therapy; DC, dendritic cell; mAB, monoclonal antibody; VAX, cancer vaccine; HCC, hepatocellular carcinoma, NSCLC, non-small cell lung cancer. † Number of individual studies of effect size with statistical significance in the reverse direction/not statistically significant/statistically significant. ‡ Concordance of fixed and random effects summary outcome with outcome of largest individual study. § Value obtained from re-analysis of original meta-analysis. All *p-Value*s are two-sided.

Table S9. PRISMA (Preferred Reporting Items for Systematic Reviews and Meta-Analyses) Checklist.

**Section/topic**	#	**Checklist item**	**Reported on page #**

**TITLE**			
Title	1	Identify the report as a systematic review, meta-analysis, or both.	1
**ABSTRACT**			
Structured summary	2	Provide a structured summary including, as applicable: background; objectives; data sources; study eligibility criteria, participants, and interventions; study appraisal and synthesis methods; results; limitations; conclusions and implications of key findings; systematic review registration number.	1
**INTRODUCTION**			
Rationale	3	Describe the rationale for the review in the context of what is already known.	1-2
Objectives	4	Provide an explicit statement of questions being addressed with reference to participants, interventions, comparisons, outcomes, and study design (PICOS).	1-2
**METHODS**			
Protocol and registration	5	Indicate if a review protocol exists, if and where it can be accessed (e.g., Web address), and, if available, provide registration information including registration number.	14
Eligibility criteria	6	Specify study characteristics (e.g., PICOS, length of follow-up) and report characteristics (e.g., years considered, language, publication status) used as criteria for eligibility, giving rationale.	14
Information sources	7	Describe all information sources (e.g., databases with dates of coverage, contact with study authors to identify additional studies) in the search and date last searched.	14
Search	8	Present full electronic search strategy for at least one database, including any limits used, such that it could be repeated.	14
Study selection	9	State the process for selecting studies (i.e., screening, eligibility, included in systematic review, and, if applicable, included in the meta-analysis).	14, figure S1
Data collection process	10	Describe method of data extraction from reports (e.g., piloted forms, independently, in duplicate) and any processes for obtaining and confirming data from investigators.	14-15
Data items	11	List and define all variables for which data were sought (e.g., PICOS, funding sources) and any assumptions and simplifications made.	14-15
Risk of bias in individual studies	12	Describe methods used for assessing risk of bias of individual studies (including specification of whether this was done at the study or outcome level), and how this information is to be used in any data synthesis.	15
Summary measures	13	State the principal summary measures (e.g., risk ratio, difference in means).	15
Synthesis of results	14	Describe the methods of handling data and combining results of studies, if done, including measures of consistency (e.g., I^2) for each meta-analysis.	15-16, figure S8

**Section/topic**	**#**	**Checklist item**	**Reported on page #**
Risk of bias across studies	15	Specify any assessment of risk of bias that may affect the cumulative evidence (e.g., publication bias, selective reporting within studies).	15
Additional analyses	16	Describe methods of additional analyses (e.g., sensitivity or subgroup analyses, meta-regression), if done, indicating which were pre-specified.	15
		**RESULTS**	
Study selection	17	Give numbers of studies screened, assessed for eligibility, and included in the review, with reasons for exclusions at each stage, ideally with a flow diagram.	2-3
Study characteristics	18	For each study, present characteristics for which data were extracted (e.g., study size, PICOS, follow-up period) and provide the citations.	Table S2-S8, reference appendix
Risk of bias within studies	19	Present data on risk of bias of each study and, if available, any outcome level assessment (see item 12).	Table S10
Results of individual studies	20	For all outcomes considered (benefits or harms), present, for each study: (a) simple summary data for each intervention group (b) effect estimates and confidence intervals, ideally with a forest plot.	2-12, Table 1-3
Synthesis of results	21	Present results of each meta-analysis done, including confidence intervals and measures of consistency.	Table 1-3, Table S2-S8, Fig S2-S7
Risk of bias across studies	22	Present results of any assessment of risk of bias across studies (see Item 15).	2-12, Table 1-3, Table S2-S8
Additional analysis	23	Give results of additional analyses, if done (e.g., sensitivity or subgroup analyses, meta-regression [see Item 16]).	Table S8
		**DISCUSSION**	
Summary of evidence	24	Summarize the main findings including the strength of evidence for each main outcome; consider their relevance to key groups (e.g., healthcare providers, users, and policy makers).	12-13
Limitations	25	Discuss limitations at study and outcome level (e.g., risk of bias), and at review-level (e.g., incomplete retrieval of identified research, reporting bias).	13-14
Conclusions	26	Provide a general interpretation of the results in the context of other evidence, and implications for future research.	16
		**FUNDING**	
Funding	27	Describe sources of funding for the systematic review and other support (e.g., supply of data); role of funders for the	17 (None)

systematic review.

From: Moher D, Liberati A, Tetzlaff J, Altman DG, The PRISMA Group (2009). Preferred Reporting Items for Systematic Reviews and Meta-Analyses: The PRISMA Statement. PLoS Med 6(7): e1000097. doi:10.1371/journal.pmed1000097. For more information, visit: **www.prisma-statement.org**.

Table S10. Quality of the individual studies in the eligible meta-analyses using the Cochrane risk of bias tool.

Author, year	Random sequence generation (selection bias)	Allocation concealment (selection bias)	Blinding of patients and personnel (performance bias)	Blinding of outcome assessment (detection bias)	Incomplete outcome data (attrition bias)	Selective reporting bias (reporting bias)	Other bias
**Bai et al. 2018**							
Aitchison 2014	+	?	?	+	+	+	?
Chamie 2016	+	+	?	+	+	?	+
Galligioni 1996	?	+	?	+	?	+	?
Hass 2016	+	+	+	?	?	+	+
Hinotsu 2013	+	?	?	+	+	+	?
Jocham 2004	+	+	?	+	-	?	?
Margulis 2009	+	?	?	+	+	+	?
Messing 2003	+	?	?	+	+	+	?
Passalacqua 2014	+	+	?	+	+	?	+
Pizzocaro 2001	?	?	?	+	+	+	?
Ravaud 2017	+	+	+	+	+	+	+
Wood 2008	+	?	?	+	+	?	+
**Bauer et al. 2012**							
CALBG 9712	?	?	+	?	+	?	?
CLL2007FMP	?	?	?	?	?	-	-
GCLLSG CLL 8	+	+	+	?	+	+	?
Gribben 2005	?	?	?	?	?	-	-
NCRI-CLL 201	?	?	+	?	+	+	?
REACH	?	?	+	?	+	+	?
Wierda 2011	?	?	?	?	+	-	?
**Chen et al. 2014**							
Ma 2012	+	+	?	+	+	?	+
Shi 2012	+	+	?	+	+	+	+
Yang 2013	?	?	-	+	+	?	+
Zhan 2012	+	+	+	?	+	+	+
Zheng 2012	+	+	?	+	+	?	+
Zhong 2011	?	?	-	+	+	?	+
**Dammeijer et al. 2016**							
Alfonso 2014	-	+	+	?	?	+	+
Butts 2005	+	+	+	-	+	+	-
Butts 2014	?	+	?	-	+	+	+
Giaccone 2015	+	?	+	+	+	+	+
Jin 2014	+	+	?	?	?	?	?
Khranovska 2013	?	?	?	?	?	?	?
Kimura 2015	+	?	-	-	+	+	+
Li 2012	-	?	?	?	+	+	+
Neninger vinegaras 2008	+	+	?	?	+	+	+
Nokihara 2015	?	?	+	+	?	+	+

Quoix 2011	+	-	-	-	+	+	+
Quoix 2012	?	?	+	+	?	+	+
Shi 2012	?	?	?	?	+	+	+
Shi 2014	-	?	?	?	?	+	+
Vansteenkiste 2013	?	?	+	-	+	+	+
Vansteenkiste 2014	?	?	+	?	?	+	?
Wu 2008	?	?	?	?	+	+	+
Zhao 2014	?	?	?	?	?	+	+
**Hao et al. 2017**							
Checkmate037	+	?	-	-	+	+	?
Checkmate066	?	?	+	+	+	+	?
Checkmate067	?	?	+	+	+	+	?
Checkmate069	?	?	+	+	+	+	?
KeyNote002	+	+	+	+	+	+	?
KeyNote006	?	?	-	-	+	+	?
**Jiang et al. 2013**							
Ishikawa 2012	+	?	?	?	+	+	+
Kubo 2002	+	?	+	+	+	+	+
Li 2009	+	?	?	?	+	+	+
Li 2010	+	+	?	?	+	+	+
Lin 2004	?	?	?	?	+	+	+
Lo 2007	+	+	?	?	+	+	+
Mazzaferro 2006	+	?	?	?	+	+	+
Paio 2005	+	?	+	+	+	+	+
Shiratori 2003	+	+	+	+	+	+	+
Sun 2006	+	+	+	+	+	+	+
**Lan et al. 2015**							
Gao 2014	?	?	+	+	+	?	+
Li 2009	+	?	+	+	+	?	?
Li 2012	+	?	+	+	+	?	+
Ni 2013	?	-	+	+	+	?	?
Ren 2013	+	?	+	+	+	?	+
Sheng 2011	?	?	+	+	+	?	+
Yuan 2011	+	-	+	+	+	?	+
Yuan 2011	+	-	+	+	+	?	+
Yuan 2013	?	-	+	+	+	?	?
Zhang 2011	?	?	+	+	+	?	+
Zhong 2011	?	?	+	+	+	?	+
Zhu 2011	+	?	+	+	+	?	+
**Li et al. 2016**							
Dong 2009	+	+	-	?	+	+	+
Lee 2015	+	+	-	+	+	+	+
Pan 2010	+	+	-	?	+	+	+
Takayama 2000	+	+	-	+	+	+	+
Weng 2008	+	+	-	?	+	+	+
Xu 2013	+	?	-	?	+	+	+

Yu 2014	+	+	-	+	+	+	+
Zhao 2008	+	+	-	?	+	+	+
**Mao et al. 2015**							
Baer 2008	?	?	+	-	+	+	+
Blaise 2000	?	?	+	-	+	?	+
Faber 1997	?	?	+	-	?	?	?
Kolitz 2014	?	?	+	-	+	+	+
Lange 2011	?	+	+	-	+	+	+
Liu 2011	+	?	?	?	?	+	?
Pautas 2010	?	?	+	-	+	+	+
Petit 2014	?	?	+	-	+	+	+
Willemze 2011	?	+	+	-	+	+	+
**Mocellin et al. 2013**							
Agarwala 2011	?	?	+	+	?	?	+
Cameron 2001	?	?	+	+	+	+	+
Cascinelli 2001	?	+	+	+	+	+	+
Creagan 1995	?	?	+	+	+	+	+
Eggermont 2005	+	+	+	+	+	+	+
Eggermont 2008	+	+	+	+	+	+	+
Garbe 2008	+	+	+	+	+	+	+
Grob 1998	+	+	+	+	+	+	+
Hancock 2004	+	+	+	+	+	+	+
Hansson 2011	+	+	+	+	+	+	+
Kirkwood 1996	+	?	+	+	+	+	+
Kirkwood 2000	+	?	+	+	+	+	+
Kirkwood 2001	?	+	+	+	+	+	+
Kirkwood 2001	?	+	+	+	+	+	+
Kleeberg 2004	?	+	+	+	+	+	+
McMasters 2008	?	?	+	+	?	?	+
Pehamberger 1998	?	?	+	+	+	+	+
Rusciani 1997	-	-	+	+	?	-	+
**Sasse et al. 2005**							
Atkins 2003	?	?	-	?	+	-	+
Atzpodien 2002	+	+	-	?	+	+	+
Bajetta 1994	+	+	-	?	+	+	+
Danson 2003	+	+	-	?	+	+	+
Del Vecchio 2003	?	?	-	?	+	+	+
Eton 2002	?	?	-	?	+	-	+
Falkson 1991	?	?	-	?	+	-	+
Falkson 1998	+	+	-	?	+	+	+
Gorbonova 2000	?	?	-	?	+	-	+
Johnston 1998	+	+	-	?	+	+	+
Kirkwood 1990	+	?	-	?	+	-	+
Middleton 2000	+	+	-	?	+	+	+
Ridolfi 2002	+	+	-	?	+	+	+
Rosenberg 1999	+	+	-	?	+	+	+
Spieth 2003	?	?	-	?	+	-	+

Thomson 1993	+	+	-	?	+	-	+
Vorobiof 1994	+	+	-	?	+	+	+
Young 2001	+	+	-	?	+	+	+
**Unverzagt et al. 2017**							
Amato 2010	?	?	+	+	+	-	-
Escudier 2007	+	+	+	+	+	+	-
Hudes 2007	?	?	+	+	+	+	+
Motzer 2007	+	?	+	+	+	+	-
Motzer 2015	+	+	+	+	+	+	-
Negrier 2011	+	+	+	+	+	-	-
Rini 2010	+	?	+	+	+	+	-
Rini 2015	?	+	+	+	+	+	+
**Wang et al. 2015**							
Li 2009	+	+	+	+	+	+	+
Shi 2012	+	+	+	+	+	+	+
Shi 2014	+	+	+	+	+	+	+
Yang 2013	+	+	+	+	+	+	+
Zhao 2014	+	+	+	+	+	+	+
Zhong 2011	+	+	+	+	+	+	+
**Wang et al. 2015**							
Alfonso 2014	+	+	+	+	+	+	+
Butts 2005	+	+	?	?	+	+	+
Butts 2011	+	+	?	?	+	+	+
Butts 2014	+	+	+	+	+	+	+
Manegold 2008	+	+	?	?	+	+	+
Mitchell 2013	+	+	+	+	+	+	+
Nemunaitis 2004	+	?	?	?	+	+	+
Nemunaitis 2006	+	?	?	?	+	+	+
O'Brien 2004	+	+	?	?	+	+	+
Quiox 2011	+	+	?	?	+	+	+
Vinageras 2008	+	+	?	?	+	+	+
**Wu et al. 2017**							
Antonia 2016	-	?	-	-	+	?	?
Hodi 2010	+	+	+	+	+	+	+
Hodi 2014	+	+	-	+	+	+	?
Hodi 2016	+	+	+	+	+	+	?
Larkin 2015	+	+	+	+	?	+	+
Lynch 2011	+	?	+	?	+	?	+
Reck 2013	+	?	+	?	+	?	?
Robert 2011	+	?	+	?	+	+	+
**Xin et al. 2016**							
Antoni 2013	+	+	?	?	+	+	+
Caria 1991	+	+	?	?	+	+	+
Caria 1995	+	+	?	?	+	+	+
Caria 1998	+	+	?	?	+	+	+
Emilio 1994	+	+	?	?	+	+	+
Galvez 1991	?	?	?	?	?	?	?
Thomson 1993	?	?	?	?	?	?	?

Young 2001	+	+	?	?	+	+	+
ZRUDOLF 1996	?	+	?	?	+	+	+
**Yu et al. 2017**							
Deng 2013	+	?	?	?	?	?	?
Dong 2009	+	?	-	?	+	+	+
Guo 2014	-	-	-	?	+	+	-
Hao 2006	-	-	-	?	+	+	-
Hao 2010	-	-	-	?	+	+	-
He 2012	+	?	?	?	?	?	?
Huang 2007	+	?	?	?	?	+	?
Huang 2013	-	-	-	?	+	+	-
Lee 2015	+	+	-	+	+	+	+
Pan 2013	-	-	-	?	+	+	-
Tong 2013	-	-	-	?	?	+	-
Wang 2012	-	-	-	?	+	+	-
Weng 2009	+	?	?	?	+	+	+
Yu 2009	-	-	-	?	+	+	-
Yu 2014	+	+	-	+	+	+	+
Yue 2007	-	-	-	?	?	+	-
Zhang 2006	+	?	?	?	?	+	?
**Yun et al. 2016**							
Hodi 2010	+	+	+	+	+	+	?
Ribas 2013	+	+	?	?	+	+	?
Ribas 2015	+	+	+	+	+	+	?
Robert 2011	+	+	+	+	+	+	?
Robert 2015	+	+	+	+	+	+	?
Weber 2015	+	+	+	+	+	+	?
**Zeng et al. 2016**							
Kimura 1997	+	+	?	?	+	+	+
Kimura 2015	+	+	?	?	+	+	+
Ratto 1996	+	+	?	+	+	+	+
Zhao 2014	+	?	?	?	+	+	+
**Zheng et al. 2015**							
Li 2009	+	+	+	-	+	+	+
Shi 2012	+	-	+	+	+	+	+
Yang 2013	+	-	+	-	-	+	+
Zhang 2012	+	+	+	-	-	-	+
Zhao 2014	+	-	+	+	+	+	+
Zhong 2011	-	-	+	+	-	+	+
**Zhou et al. 2017**							
Gisselbrecht 2012	+	-	-	-	+	+	+
Habermann 2006	+	-	-	-	+	+	-
Haioun 2009	+	-	-	-	+	+	-
Harig 2015	+	-	-	-	+	-	-
Jaeger 2015	+	-	-	-	+	+	-
**Zhu et al. 2015**							
Chen 2012	+	?	?	?	+	+	+

Chung 2013	+	+	?	?	+	+	+
Dong 2008	?	+	+	+	+	+	+
Edward 1998	+	+	?	?	+	+	+
Hasegawa 2006	?	?	-	-	+	+	+
Lau 1996	+	?	?	?	+	+	+
Lo 2007	+	-	-	-	+	+	?
Mazzaferro 2006	+	-	+	+	+	+	+
Nishiguchi 2005	?	?	?	?	+	+	+
Ono 1997	+	?	?	?	+	?	+
Sun 2006	?	+	+	+	+	+	+
Tadatoshi 2000	+	+	?	?	+	+	+
Xia 2010	+	?	?	?	+	+	?
Yamamoto 1996	?	?	?	?	+	+	+
**Zhuansun et al. 2017**							
Borghaei 2015	+	+	+	+	+	+	+
Brahmer 2015	+	+	+	+	+	+	+
Fehrenbacher 2016	+	+	+	+	+	+	+
Herbst 2016	+	+	+	+	+	+	-

Reference list of the eligible articles

44. Bai Y, Li S, Jia Z, Ding Y, Gu C, Yang J. Adjuvant therapy for locally advanced renal cell carcinoma: A meta-analysis and systematic review. Urol Oncol. 2018;36(2):79.e1-79.e10, doi:10.1016/j.urolonc.2017.10.001.
45. Baldo P, Rupolo M, Compagnoni A, et al. Interferon-alpha for maintenance of follicular lymphoma. Cochrane database Syst Rev. 2010;(1):CD004629, doi:10.1002/14651858.CD004629.pub2.
46. Bauer K, Rancea M, Roloff V, et al. Rituximab, ofatumumab and other monoclonal anti-CD20 antibodies for chronic lymphocytic leukaemia. Cochrane database Syst Rev. 2012;11:CD008079, doi:10.1002/14651858.CD008079.pub2.
47. Buyse M, Squifflet P, Lange BJ, et al. Individual patient data meta-analysis of randomized trials evaluating IL-2 monotherapy as remission maintenance therapy in acute myeloid leukemia. Blood. 2011;117(26):7007-7013, doi:10.1182/blood-2011-02-337725.
48. Canil C, Hotte S, Mayhew L a, Waldron TS, Winquist E. Interferon-alfa in the treatment of patients with inoperable locally advanced or metastatic renal cell carcinoma: A systematic review. Can Urol Assoc J. 2010;4(3):201-208. http://www.pubmedcentral.nih.gov/articlerender.fcgi?artid = 2874597&tool = pmcentrez&rendertype = abstract.
49. Cao J-X, Zhang X-Y, Liu J-L, et al. Clinical efficacy of tumor antigen-pulsed DC treatment for high-grade glioma patients: Evidence from a meta-analysis. *PLoS ONE*. 2014;9(9):e107173, doi:10.1371/journal.pone.0107173.
50. Chen R, Deng X, Wu H, et al. Combined immunotherapy with dendritic cells and cytokine-induced killer cells for malignant tumors: A systematic review and meta-analysis. Int Immunopharmacol. 2014;22(2):451-464, doi:10.1016/j.intimp.2014.07.019.
51. Dammeijer F, Lievense LA, Veerman GDM, et al. Efficacy of Tumor Vaccines and Cellular Immunotherapies in Non-Small-Cell Lung Cancer: A Systematic Review and Meta-Analysis. J Clin Oncol. 2016;34(26):3204-3212, doi:10.1200/JCO.2015.66.3955.
52. Ding M, Yang J. Therapeutic vaccination for non-small-cell lung cancer: A meta-analysis. Med Oncol. 2014;31(4):928, doi:10.1007/s12032-014-0928-1.
53. Gao G, Liang X, Jiang J, et al. A systematic review and meta-analysis of immunochemotherapy with rituximab for B-cell non-Hodgkin's lymphoma. Acta Oncol. 2010;49(1):3-12, doi:10.3109/02841860903150502.
54. Guan X, Wang H, Ma F, Qian H, Yi Z, Xu B. The Efficacy and Safety of Programmed Cell Death 1 and Programmed Cell Death 1 Ligand Inhibitors for Advanced Melanoma: A Meta-Analysis of Clinical Trials

Following the PRISMA Guidelines. Medicine (Baltimore). 2016;95(11):e3134, doi:10.1097/MD.0000000000003134.
55. Hamm C, Verma S, Petrella T, Bak K, Charette M, Melanoma Disease Site Group of Cancer Care Ontario's Program in Evidence-based Care. Biochemotherapy for the treatment of metastatic malignant melanoma: A systematic review. Cancer Treat Rev. 2008;34(2):145-156, doi:10.1016/j.ctrv.2007.10.003.
56. Han R, Liu X, Pan P, Jia Y, Yu J. Effectiveness and safety of chemotherapy combined with dendritic cells co-cultured with cytokine-induced killer cells in the treatment of advanced non-small-cell lung cancer: A systematic review and meta-analysis. *PLoS ONE*. 2014;9(9):e108958, doi:10.1371/journal.pone.0108958.
57. Hao C, Tian J, Liu H, Li F, Niu H, Zhu B. Efficacy and safety of anti-PD-1 and anti-PD-1 combined with anti-CTLA-4 immunotherapy to advanced melanoma: A systematic review and meta-analysis of randomized controlled trials. Medicine (Baltimore). 2017;96(26):e7325, doi:10.1097/MD.0000000000007325.
58. Hotte S, Waldron T, Canil C, Winquist E. Interleukin-2 in the treatment of unresectable or metastatic renal cell cancer: A systematic review and practice guideline. Can Urol Assoc J. 2007;1(1):27-38. http://www.pubmedcentral.nih.gov/articlerender.fcgi?artid = 2422922&tool = pmcentrez&rendertype = abstract.
59. Hou S, Yang W. A meta-analysis on Rituximab combined CHOP chemotherapy for non-Hodgkin lymphoma in China. Saudi Med J. 2011;32(7):675-678. http://www.ncbi.nlm.nih.gov/pubmed/21748202.
60. Ives NJ, Stowe RL, Lorigan P, Wheatley K. Chemotherapy compared with biochemotherapy for the treatment of metastatic melanoma: A meta-analysis of 18 trials involving 2,621 patients. J Clin Oncol. 2007;25(34):5426-5434, doi:10.1200/JCO.2007.12.0253.
61. Ives NJ, Suciu S, Eggermont AMM, et al. Adjuvant interferon-α for the treatment of high-risk melanoma: An individual patient data meta-analysis. Eur J Cancer. 2017;82:171-183, doi:10.1016/j.ejca.2017.06.006.
62. Jiang S, Liu Y, Wang L, Duan C, Liu M. A meta-analysis and systematic review: Adjuvant interferon therapy for patients with viral hepatitis-related hepatocellular carcinoma. World J Surg Oncol. 2013;11(1):240, doi:10.1186/1477-7819-11-240.
63. Kawalec P, Paszulewicz A, Holko P, Pilc A. Sipuleucel-T immunotherapy for castration-resistant prostate cancer. A systematic review and meta-analysis. Arch Med Sci. 2012;8(5):767-775, doi:10.5114/aoms.2012.31610.
64. Lan X-P, Chen Y-G, Wang Z, et al. Immunotherapy of DC-CIK cells enhances the efficacy of chemotherapy for solid cancer: A meta-analysis of randomized controlled trials in Chinese patients. J Zhejiang Univ Sci B. 2015;16(9):743-756, doi:10.1631/jzus.B1500003.
65. Li Y-C, Zhao L, Wu J-P, Qu C-X, Song Q-K, Wang R-B. Cytokine-induced killer cell infusion combined with conventional treatments produced better prognosis for hepatocellular carcinoma patients with barcelona clinic liver cancer B or earlier stage: A systematic review and meta-analysis. Cytotherapy. 2016;18(12):1525-1531, doi: 10.1016/j.jcyt.2016.09.002.
66. Mao C, Fu X-H, Yuan J-Q, et al. Interleukin-2 as maintenance therapy for children and adults with acute myeloid leukaemia in first complete remission. Cochrane database Syst Rev. 2015;(11):CD010248, doi: 10.1002/14651858.CD010248.pub2.
67. Massari F, Bria E, Maines F, et al. Adjuvant treatment for resected renal cell carcinoma: Are all strategies equally negative? Potential implications for trial design with targeted agents. Clin Genitourin Cancer. 2013;11(4):471-476, doi: 10.1016/j.clgc.2013.04.018.
68. Mi D, Ren W, Yang K. Adoptive immunotherapy with interleukin-2 & induced killer cells in non-small cell lung cancer: A systematic review & meta-analysis. Indian J Med Res. 2016;143(Supplement):S1-S10, doi:10.4103/0971-5916.191738.
69. Mocellin S, Lens MB, Pasquali S, Pilati P, Chiarion Sileni V. Interferon alpha for the adjuvant treatment of cutaneous melanoma. Cochrane database Syst Rev. 2013;6(6):CD008955, doi: 10.1002/14651858.CD008955.pub2.
70. Myeloma Trialists' Collaborative Group. Interferon as therapy for multiple myeloma: An individual patient data overview of 24 randomized trials and 4012 patients. Br J Haematol. 2001;113(4):1020-1034. http://ovidsp.ovid.com/ovidweb.cgi?T = JS&PAGE = reference&D = med4&NEWS = N&AN = 11442498.
71. Nunes AA, da Silva AS, Souza KM, Koury C de NS, de Mello LM. Rituximab, fludarabine, and cyclophosphamide versus fludarabine and cyclophosphamide for treatment of chronic lymphocytic leukemia: A systematic review with meta-analysis. Crit Rev Oncol Hematol. 2015;94(3):261-269, doi: 10.1016/j.critrevonc.2015.02.013.

72. Pirard D, Heenen M, Melot C, Vereecken P. Interferon alpha as adjuvant postsurgical treatment of melanoma: A meta-analysis. Dermatology. 2004;208(1):43-48, doi:10.1159/000075045.
73. Ren Y-R, Jin Y-D, Zhang Z-H, Li L, Wu P. Rituximab treatment strategy for patients with diffuse large B-cell lymphoma after first-line therapy: A systematic review and meta-analysis. Chin Med J (Engl). 2015;128(3):378-383, doi:10.4103/0366-6999.150111.
74. Rossi A, Garassino MC, Cinquini M, et al. Maintenance or consolidation therapy in small-cell lung cancer: A systematic review and meta-analysis. Lung Cancer. 2010;70(2):119-128, doi: 10.1016/j.lungcan.2010.02.001.
75. Roviello G, Zanotti L, Correale P, et al. Is still there a role for IL-2 for solid tumors other than melanoma or renal cancer? Immunotherapy. 2017;9(1):25-32, doi:10.2217/imt-2016-0107.
76. Sasse AD, Sasse EC, Clark LGO, Ulloa L, Clark OAC. Chemoimmunotherapy versus chemotherapy for metastatic malignant melanoma. Cochrane database Syst Rev. 2007;(1):CD005413, doi: 10.1002/14651858.CD005413.pub2.
77. Scherr AJO, Lima JPSN, Sasse EC, Lima CSP, Sasse AD. Adjuvant therapy for locally advanced renal cell cancer: A systematic review with meta-analysis. BMC Cancer. 2011;11(1):115, doi:10.1186/1471-2407-11-115.
78. Schulz H, Bohlius JF, Trelle S, et al. Immunochemotherapy with rituximab and overall survival in patients with indolent or mantle cell lymphoma: A systematic review and meta-analysis. J Natl Cancer Inst. 2007;99(9):706-714, doi:10.1093/jnci/djk152.
79. Su Y, Yang Y, Ma Y, et al. The Efficacy and Safety of Dendritic Cells Co-Cultured with Cytokine-Induced Killer Cell Therapy in Combination with TACE-Predominant Minimally-Invasive Treatment for Hepatocellular Carcinoma: A Meta-Analysis. Clin Lab. 2016;62(4):599-608, doi:10.7754/Clin.Lab.2015.150804.
80. Tang X, Liu T, Zang X, et al. Adoptive cellular immunotherapy in metastatic renal cell carcinoma: A systematic review and meta-analysis. *PLoS ONE*. 2013;8(5):e62847, doi:10.1371/journal.pone.0062847.
81. Thirion P, Piedbois P, Buyse M, et al. Alpha-interferon does not increase the efficacy of 5-fluorouracil in advanced colorectal cancer. Br J Cancer. 2001;84(5):611-620, doi:10.1054/bjoc.2000.1669.
82. Unverzagt S, Moldenhauer I, Nothacker M, et al. Immunotherapy for metastatic renal cell carcinoma. Cochrane database Syst Rev. 2017;5(5):CD011673, doi: 10.1002/14651858.CD011673.pub2.
83. Verma S, Quirt I, McCready D, Bak K, Charette M, Iscoe N. Systematic review of systemic adjuvant therapy for patients at high risk for recurrent melanoma. Cancer. 2006;106(7):1431-1442, doi:10.1002/cncr.21760.
84. Vidal L, Gafter-Gvili A, Leibovici L, et al. Rituximab maintenance for the treatment of patients with follicular lymphoma: Systematic review and meta-analysis of randomized trials. J Natl Cancer Inst. 2009;101(4):248-255, doi:10.1093/jnci/djn478.
85. Vidal L, Gafter-Gvili A, Salles G, et al. Rituximab maintenance improves overall survival of patients with follicular lymphoma-Individual patient data meta-analysis. Eur J Cancer. 2017;76(March):216-225, doi: 10.1016/j.ejca.2017.01.021.
86. Wang H, Liu A, Bo W, et al. Adjuvant immunotherapy with autologous cytokine-induced killer cells for hepatocellular carcinoma patients after curative resection, a systematic review and meta-analysis. Dig Liver Dis. 2016;48(11):1275-1282, doi: 10.1016/j.dld.2016.07.010.
87. Wang J, Zou Z-H, Xia H-L, He J-X, Zhong N-S, Tao A-L. Strengths and weaknesses of immunotherapy for advanced non-small-cell lung cancer: A meta-analysis of 12 randomized controlled trials. *PLoS ONE*. 2012;7(3):e32695, doi:10.1371/journal.pone.0032695.
88. Wang M, Cao J-X, Liu Y-S, et al. Evaluation of tumour vaccine immunotherapy for the treatment of advanced non-small cell lung cancer: A systematic meta-analysis. BMJ Open. 2015;5(4):e006321, doi:10.1136/bmjopen-2014-006321.
89. Wang S, Wang Z. Efficacy and safety of dendritic cells co-cultured with cytokine-induced killer cells immunotherapy for non-small-cell lung cancer. Int Immunopharmacol. 2015;28(1):22-28, doi: 10.1016/j.intimp.2015.05.021.
90. Wang X, Bao Z, Zhang X, et al. Effectiveness and safety of PD-1/PD-L1 inhibitors in the treatment of solid tumors: A systematic review and meta-analysis. Oncotarget. 2017;8(35):59901-59914, doi:10.18632/oncotarget.18316.
91. Wheatley K, Ives N, Hancock B, Gore M, Eggermont A, Suciu S. Does adjuvant interferon-alpha for high-risk melanoma provide a worthwhile benefit? A meta-analysis of the randomised trials. Cancer Treat Rev. 2003;29(4):241-252, doi:10.1016/S0305-7372(03)00074-4.

92. Wu Y, Shi H, Jiang M, et al. The clinical value of combination of immune checkpoint inhibitors in cancer patients: A meta-analysis of efficacy and safety. Int J cancer. 2017;141(12):2562-2570, doi:10.1002/ijc.31012.
93. Xie F, Zhang X, Li H, et al. Adoptive immunotherapy in postoperative hepatocellular carcinoma: A systemic review. *PLoS ONE*. 2012;7(8):e42879, doi:10.1371/journal.pone.0042879.
94. Xin Y, Huang Q, Zhang P, et al. Meta-Analysis of the Safety and Efficacy of Interferon Combined With Dacarbazine Versus Dacarbazine Alone in Cutaneous Malignant Melanoma. Medicine (Baltimore). 2016;95(16):e3406, doi:10.1097/MD.0000000000003406.
95. Yu D-P, Cheng X, Liu Z-D, Xu S-F. Comparative beneficiary effects of immunotherapy against chemotherapy in patients with advanced NSCLC: Meta-analysis and systematic review. Oncol Lett. 2017;14(2):1568-1580, doi:10.3892/ol.2017.6274.
96. Yu R, Yang B, Chi X, et al. Efficacy of cytokine-induced killer cell infusion as an adjuvant immunotherapy for hepatocellular carcinoma: A systematic review and meta-analysis. Drug Des Devel Ther. 2017;11:851-864, doi:10.2147/DDDT.S124399.
97. Yuan B-H, Li R-H, Yuan W, et al. Harms and benefits of adoptive immunotherapy for postoperative hepatocellular carcinoma: An updated review. Oncotarget. 2017;8(11):18537-18549, doi:10.18632/oncotarget.14507.
98. Yun S, Vincelette ND, Green MR, Wahner Hendrickson AE, Abraham I. Targeting immune checkpoints in unresectable metastatic cutaneous melanoma: A systematic review and meta-analysis of anti-CTLA-4 and anti-PD-1 agents trials. Cancer Med. 2016;5(7):1481-1491, doi:10.1002/cam4.732.
99. Zeng Y, Ruan W, He J, et al. Adoptive Immunotherapy in Postoperative Non-Small-Cell Lung Cancer: A Systematic Review and Meta-Analysis. *PLoS ONE*. 2016;11(9):e0162630, doi:10.1371/journal.pone.0162630.
100. Zhao B, Zhang W, Yu D, Xu J, Wei Y. Adoptive immunotherapy shows encouraging benefit on non-small cell lung cancer: A systematic review and meta-analysis. Oncotarget. 2017;8(68):113105-113119, doi:10.18632/oncotarget.19373.
101. Zheng C, Yu G, Wang H, et al. Meta-analysis of chemotherapy and dendritic cells with cytokine-induced killer cells in the treatment of non-small-cell lung cancer. Int J Clin Exp Med. 2015;8(8):14527-14537. http://www.ncbi.nlm.nih.gov/pubmed/26550444.
102. Zhou G-W, Xiong Y, Chen S, Xia F, Li Q, Hu J. Anti-PD-1/PD-L1 antibody therapy for pretreated advanced nonsmall-cell lung cancer: A meta-analysis of randomized clinical trials. Medicine (Baltimore). 2016;95(35):e4611, doi:10.1097/MD.0000000000004611.
103. Zhou L, Wang X-L, Deng Q-L, Du Y-Q, Zhao N-Q. The efficacy and safety of immunotherapy in patients with advanced NSCLC: A systematic review and meta-analysis. Sci Rep. 2016;6(1):32020, doi:10.1038/srep32020.
104. Zhou X, Ma T, Zhang Y, Zhou N, Li J. Rituximab maintenance therapy for patients with diffuse large B-cell lymphoma: A meta-analysis. *PLoS ONE*. 2017;12(3):e0174648, doi:10.1371/journal.pone.0174648.
105. Zhu G-Q, Shi K-Q, Yu H-J, et al. Optimal adjuvant therapy for resected hepatocellular carcinoma: A systematic review with network meta-analysis. Oncotarget. 2015;6(20):18151-18161, doi:10.18632/oncotarget.4098.
106. Zhuansun Y, Huang F, Du Y, Lin L, Chen R, Li J. Anti-PD-1/PD-L1 antibody versus conventional chemotherapy for previously-treated, advanced non-small-cell lung cancer: A meta-analysis of randomized controlled trials. J Thorac Dis. 2017;9(3):655-665, doi:10.21037/jtd.2017.03.104.

© 2019 by the authors. Licensee MDPI, Basel, Switzerland. This article is an open access article distributed under the terms and conditions of the Creative Commons Attribution (CC BY) license (http://creativecommons.org/licenses/by/4.0/).
